# CCR2^+^ monocytes infiltrate atrophic lesions in age-related macular disease and mediate photoreceptor degeneration in experimental subretinal inflammation in *Cx3cr1* deficient mice

**DOI:** 10.1002/emmm.201302692

**Published:** 2013-10-21

**Authors:** Florian Sennlaub, Constance Auvynet, Bertrand Calippe, Sophie Lavalette, Lucie Poupel, Shulong J Hu, Elisa Dominguez, Serge Camelo, Olivier Levy, Elodie Guyon, Noah Saederup, Israel F Charo, Nico Van Rooijen, Emeline Nandrot, Jean-Louis Bourges, Francine Behar-Cohen, José-Alain Sahel, Xavier Guillonneau, William Raoul, Christophe Combadiere

**Affiliations:** 1 Inserm, U 968Paris, France; 2 UPMC Univ Paris 06, UMR_S 968, Institut de la VisionParis, France; 3 Centre Hospitalier National d'Ophtalmologie des Quinze-Vingts, INSERM-DHOS CIC 503Paris, France; 4 Hôtel Dieu, Service d'Ophtalmologie, Centre de Recherche OphtalmologiqueParis, France; 5 Inserm UMR_S 945, Laboratoire Immunité et InfectionParis, France; 6 Université Pierre et Marie Curie-Paris6, UPMC Univ Paris 06, UMR_S 945Paris, France; 7 Gladstone Institute of Cardiovascular Disease, San FranciscoCA, USA; 8 Cardiovascular Research Institute, Department of Medicine, University of California San FranciscoSan Francisco, CA, USA; 9 Department of Molecular Cell Biology, Free University Medical CenterAmsterdam, The Netherlands; 10 Inserm, UMR_S 872, Centre de Recherche des CordeliersParis, France; 11 Université Paris Descartes, UMR_S 872, Centre de Recherche des CordeliersParis, France; 12 Université Pierre et Marie Curie-Paris6, UPMC Univ Paris 06, UMR_S 872Paris, France; 13 AP-HP, Groupe Hospitalier Pitié-Salpétrière, Service d'ImmunologieParis, France

**Keywords:** age-related macular disease, chemokines, monocyte, neurodegeneration, neuroinflammation

## Abstract

Atrophic age-related macular degeneration (AMD) is associated with the subretinal accumulation of mononuclear phagocytes (MPs). Their role in promoting or inhibiting retinal degeneration is unknown. We here show that atrophic AMD is associated with increased intraocular CCL2 levels and subretinal CCR2^+^ inflammatory monocyte infiltration in patients. Using age- and light-induced subretinal inflammation and photoreceptor degeneration in *Cx3cr1* knockout mice, we show that subretinal *Cx3cr1* deficient MPs overexpress CCL2 and that both the genetic deletion of CCL2 or CCR2 and the pharmacological inhibition of CCR2 prevent inflammatory monocyte recruitment, MP accumulation and photoreceptor degeneration *in vivo*. Our study shows that contrary to CCR2 and CCL2, CX3CR1 is constitutively expressed in the retina where it represses the expression of CCL2 and the recruitment of neurotoxic inflammatory CCR2^+^ monocytes. CCL2/CCR2 inhibition might represent a powerful tool for controlling inflammation and neurodegeneration in AMD.

## INTRODUCTION

Age-related macular degeneration (AMD) is the leading cause of irreversible blindness in the industrialized world (Klein et al, 2004). There are two clinical forms of late onset AMD: the fast-developing exudative form (wet AMD) defined by choroidal neovascularization (CNV) and the slow-developing atrophic form characterized by a progressing lesion of the retinal pigment epithelium (RPE) and photoreceptors known as geographic atrophy (GA) (Klein et al, 2007; van Leeuwen et al, 2003). Markers of inflammation, such as plasma levels of activated complement factor 3 (C3a) (Machalinska et al, 2009) and C-reactive protein (Hong et al, 2011), have been shown to increase in wet AMD patients. Both forms of AMD are associated with a polymorphism of Complement factor H (CfH) (Edwards et al, 2005; Haines et al, 2005; Klein et al, 2005; Maller et al, 2006).

In GA patients, cells that are positive for microglial cell (MC)/macrophage (Mφ) markers accumulate in the subretinal space (Gupta et al, 2003; Penfold et al, 1997). MCs and macrophages (Mφ) are part of the mononuclear phagocyte (MP) system that also includes blood monocytes (Mo), tissue macrophages and dendritic cells (DCs) (Chow et al, 2011; Ransohoff & Cardona, 2010; Wynn et al, 2013). Distinct types of MPs, exert different functions, but are difficult to distinguish immunohistochemically, as they constitutively express or induce similar markers (Gautier et al, 2012; Ransohoff & Cardona, 2010). While MCs are recognized to have neuroprotective functions (Tremblay et al, 2011), the sustained presence of inflammatory Mos (iMos) can be detrimental in neurodegenerative conditions such as multiple sclerosis and stroke (Conductier et al, 2010; Ransohoff, 2009). The nature of the MPs that accumulate in the subretinal space in GA patients is not known, as non-specific markers were used at the time of their observation (Gupta et al, 2003; Penfold et al, 1997). Their possible role in photoreceptor rescue or degeneration is unknown.

Molecular markers that differentiate iMos and inflammatory Mφs (iMφs) from MCs include CCR2 and CX3CR1: MCs do not express CCR2 (Mizutani et al, 2011), but do express high levels of CX3CR1 (Geissmann et al, 2003; Guo et al, 2012; Saederup et al, 2010). Furthermore, CCR2 cannot be induced in MCs (Saederup et al, 2010). CCL2 signalling through CCR2 and CX3CL1 signalling through CX3CR1 are key factors in Mo recruitment to a tissue lesion (Combadiere et al, 2003; Combadiere et al, 2008). The genetic deletion of *Ccl2* and *Ccr2* is associated with reduced MP accumulation in various tissue lesions, including the central nervous system (Conductier et al, 2010; Fife et al, 2000; Huang et al, 2001; Izikson et al, 2000; Ransohoff, 2009). CX3CL1 is an atypical chemokine. It is expressed as a transmembrane protein that mediates integrin-like intracellular adhesion and can be cleaved by proteases into a soluble form that has chemotactic properties (Bazan et al, 1997). The genetic deletion of *Cx3cr1* is associated with reduced MP accumulation in peripheral tissues (Combadiere et al, 2003), but MP accumulation and neuronal apoptosis are increased in the central nervous systems of *Cx3cr1*-deficient mice (Cardona et al, 2006; Ransohoff, 2009).

MPs are scarcely present in the photoreceptor cell layer and subretinal space of young, pigmented, adult wildtype mice but accumulate with age (Xu et al, 2008). In the eye, CX3CL1 is constitutively expressed as a transmembrane protein in retinal neurons (Silverman et al, 2003) and seems to provide a tonic inhibitory signal to CX3CR1 bearing retinal MCs that keeps these cells in a quiescent surveillance mode under physiological conditions (Combadiere et al, 2007; Ransohoff, 2009). *Cx3cr1* deficiency in mice leads to a strong increase of subretinal MP accumulation with age and after a light-challenge; the accumulation of *Cx3cr1*-deficient MPs is also associated with photoreceptor degeneration (Combadiere et al, 2007; Ma et al, 2009; Raoul et al, 2008a). Although these features do not mimic all the aspects of AMD (Drusen formation and RPE atrophy) they do model subretinal inflammation and associated photoreceptor degeneration, two hallmarks of AMD (Gupta et al, 2003). *Cx3cr1* deletion also increases intraretinal and subretinal MP accumulation in diabetes (Kezic et al, 2013) and intraretinal MP accumulation and retinal degeneration in a paraquat-induced retinopathy model (Chen et al, 2013).

CCL2 expression in the retina is physiologically low but is induced in situations of stress such as light-injury or retinal detachment (Chen et al, 2012; Nakazawa et al, 2007; Yamada et al, 2007). There is controversy concerning the long-term effects of *CCL2/CCR2* deficiency on retinal homeostasis. The spontaneous development of drusen (as in sub-RPE extracellular lipid accumulations), neovascularization and degeneration observed in aged *Ccl2*^*−/−*^ and *Ccr2*^*−/−*^ mice (Ambati et al, 2003), has not been reproduced in other laboratories (Chen et al, 2011; Luhmann et al, 2009). Furthermore, the early onset AMD-like phenotype (drusen-like white spots, photoreceptor and RPE atrophy before the age of 6 months) described in a *Ccl2*^*−/−*^*Cx3cr1*^*−/−*^ mouse line in numerous publications (Tuo et al, 2007) has been shown to be due to contamination with the retinal degeneration 8 (rd8) mutation (Luhmann et al, 2012; Mattapallil et al, 2012). Recently, rd8 free *Ccl2*^*−/−*^*Cx3cr1*^*−/−*^ mice have been shown to display a mild inner retinal phenotype, but no AMD-like phenotype (Vessey et al, 2012).

Clinical and experimental data suggest that elevated CCL2 expression (and not its deficiency) contributes to wet AMD pathogenesis. Increased urinary and intraocular CCL2 levels have been found in patients with wet AMD (Guymer et al, 2011; Jonas et al, 2010; Newman et al, 2012), CCL2 is induced in murine CNV (Yamada et al, 2007), and CNV is reduced in *Ccr2*^*−/−*^ and *Ccl2*^*−/−*^ mice (Luhmann et al, 2009; Tsutsumi et al, 2003). To date, little data is available concerning eventual CCL2 variations in GA. *Ccl2* mRNA expression has recently been shown to increase in all forms of AMD (Newman et al, 2012) and the CCL2/CCR2 axis is implicated in pathological inflammation and photoreceptor degeneration in chronic photo-oxidative stress (Suzuki et al, 2012), in carboxyethylpyrrole-immunization-induced retinopathy (Cruz-Guilloty et al, 2013), and in a model of retinitis pigmentosa (Guo et al, 2012).

We show that the inflamed retina in atrophic AMD produces CCL2 and that potentially neurotoxic CCR2^+^ monocytes infiltrate the diseased retina. Similarly, CCL2 levels are increased in mice with subretinal MP accumulation such as aged and photo-injured *Cx3cr1*^*−/−*^ mice. Using genetic and pharmacological approaches, we show that CCL2 attracts CCR2^+^ monocytes to the eye and participates in subretinal MP accumulation and photoreceptor degeneration in circumstances such as *Cx3cr1* deficiency and possibly AMD. CCL2/CCR2 inhibition might represent a potent therapeutic target for controlling inflammation in atrophic and wet AMD.

## RESULTS

### Intraocular CCL2 levels and CCR2^+^ inflammatory infiltrating monocytes are increased in atrophic AMD

**Figure 1:**
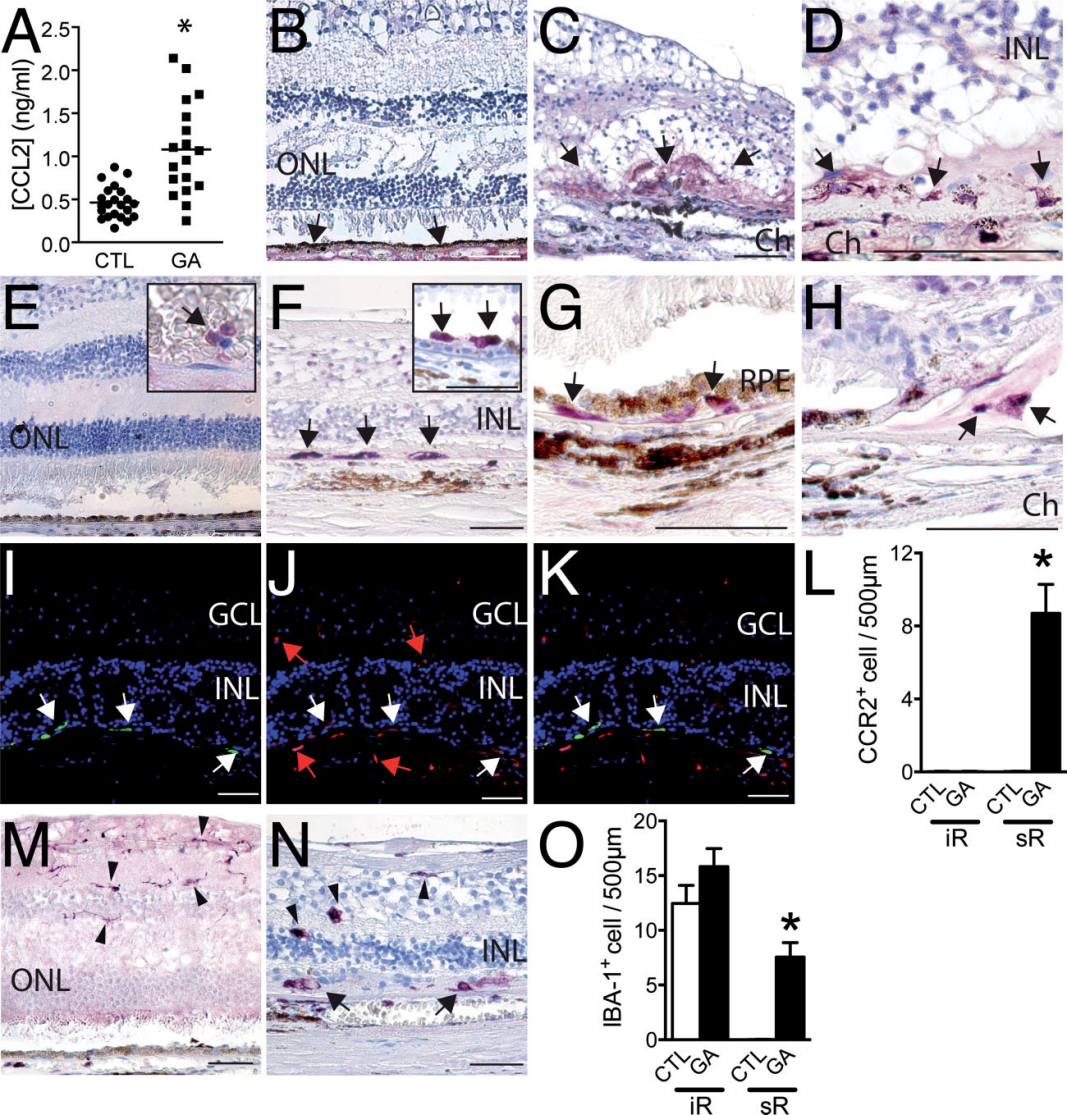
Intraocular CCL2 levels and CCR2^+^ inflammatory monocytes are increased in atrophic AMD **A.** CCL2 ELISA of aqueous humours of geographic atrophy (GA) patients and control subjects (*n* = 18 GA patients *n* = 22 control patients, student *t*-test *p* < 0.0001; Mann–Whitney test *p* < 0.0001).**B–D.** CCL2 immunohistochemistry (red staining) on macular sections of (B) control donor tissues, (C and D) within the GA lesion.**E–H.** CCR2 immunohistochemistry (red staining) on macular sections of (E) control donor tissues, inset: major retinal vessel containing erythrocytes and leucocytes, (F) within the GA lesion, inset: adjacent to GA lesion (G) laminar deposit (H) soft drusen.**I–L.** (I) CCR2 (green staining), (J) CD18 (red staining), (K) merge double labelling of a GA lesion. (L) Quantification of intraretinal and subretinal CCR2/CD18 positive cells expressed as CCR2^+^ cells/500 mm of the atrophic lesion (*n* = 10 GA donor maculae from 7 patients: age, mean (SD): 84 (8.8) and *n* = 5 control maculae from 5 patients: age, mean (SD): 83 (8.8), *students *t*-test *p* = 0.001).**M, N.** IBA-1 immunohistochemistry (red staining) on macular sections of (M) control donor tissues, (N) within the GA lesion.**O.** (O) Quantification of intraretinal and subretinal IBA-1 positive cells expressed as IBA-1^+^ cells/500 mm of the atrophic lesion (subjects same as above, *students *t*-test *p* = 0.005). B–L and N and O: representative images from 5 healthy donors, 7 donors (10 eyes) with GA and 3 donors with age related maculopathy (4 eyes), controls omitting the primary antibody showed no staining. All values are represented as mean ± SEM. CTL: control; GA: geographic atrophy; ONL: outer nuclear layer; INL: inner nuclear layer; Ch: choroid; iR: inner retina; sR: subretinal; Scale bar B–J = 50 μm.

Intraocular CCL2 levels are increased in patients with wet AMD (Jonas et al, 2010) and *Ccl2* mRNA induction is associated with all forms of AMD (Newman et al, 2012). We measured the CCL2 protein by ELISA in the aqueous humour of 18 patients that showed characteristic geographic atrophic (GA) lesions upon funduscopical examination and 22 age-matched control patients with no signs of AMD undergoing cataract surgery (see Supporting Information). CCL2 levels were significantly increased in AMD patients with GA (Fig 1A), while CX3CL1 levels were around 10 times lower and comparable in both groups (controls: 0.08 ng/ml ±0.004 SEM; GA: 0.085 ng/ml ±0.003 SEM). Next, we performed immunohistochemical analysis to analyse CCL2 expression in macular sections of donor tissues with a history of AMD and characteristic GA lesions upon postmortem fundoscopy and age-matched controls obtained from the Minnesota Lions Eye bank. Control eyes showed CCL2 staining in vessels (Fig 1B red staining, arrows), but not in the inner or outer retina. In contrast, the outer retina adjacent and within the atrophic lesion stained positive for CCL2 in all lesions analysed (Fig 1C, red staining, arrows). CCL2 positive ramified subretinal cells can be regularly observed in GA sections (Fig 1D, arrows). CCL2 staining was not observed in RPE cells or in a Müller-cell-like distribution. Distant from the atrophic lesion, CCL2 staining was observed in vessels only, similar to control sections (data not shown).

CCL2 recruits CCR2^+^ inflammatory monocytes to the inflamed tissue (Geissmann et al, 2010). To detect CCR2^+^ cells, we stained central retinal sections (containing multilayered ganglion cells) from control eyes (Fig 1E) and GA eyes (Fig 1F). First we tested the CCR2 immunohistochemistry protocol (using citrate buffer heat antigen retrieval as in paraffin section experiments) on human blood smear preparations. Human blood smear control CCR2 immunohistochemistry stained about 5% of the leukocyte population in healthy blood donors (Supporting Information data), corresponding approximately to the CX3CR1^low^CCR2^+^ inflammatory monocyte population detected by flow cytometry (Geissmann et al, 2003). Furthermore, the CCR2^+^ cells displayed morphological features of monocytes (size, nuclear shape) and lymphocytes and neutrophils did not stain positive for CCR2 (Supporting Information data). In paraffin sections of control eyes, small round CCR2^+^ cells could occasionally be observed intraluminally in major retinal vessels (Fig 1E inset) but not in the retinal tissue. Immunohistochemistry omitting the primary antibody did not reveal any staining (Supporting Information data).

In GA, small lesions of RPE and photoreceptor degeneration first appear para-foveally and then slowly expand over the posterior pole, destroying the photoreceptor layer and central vision (Sarks, 1976). We analysed central sections from GA donor maculae with visible central lesions. Late stage GA donor eyes with lesions involving the whole of the posterior pole were not included in this study. Numerous CCR2^+^ cells were observed in the subretinal space adjacent to the Bruch's membrane in the atrophic area of GA eyes (Fig 1F, arrows). CCR2^+^ cells were regularly observed on the apical side of RPE cells adjacent to the GA lesion (Fig 1F, inset, arrows; for further examples see Supporting Information figures). CCR2^+^ cells were also present in laminar deposits (Fig 1G, arrows) and soft drusen (Fig 1H, arrows; for further examples see Supporting Information figures) in all of the four examined eyes with early AMD. Double labelling of a GA lesion with leucocyte marker CD18 showed that all subretinal CCR2^+^ cells (Fig 1I green staining, arrows) also express CD18 (Fig 1J, arrows, K merge). Human monocytes and natural killer T cells can both express CCR2 and CD18 (Mantovani et al, 2004). The cells observed in the sections displayed morphological features of macrophages (nuclear shape and cytoplasm/nuclear ratio) rather than natural killer T lymphocytes, most likely identifying them as CCR2^+^ MPs (inflammatory macrophages or inflammatory DCs). Note that CD18^+^CCR2^neg^ (Fig 1J, red arrows) can be observed in the retina and choroid, where resident macrophages, MCs and DCs are located (for close views of double labelling see Supporting Information figures). Quantification of the number of CCR2^+^ MPs in the inner retina and the subretinal space in 5 control maculae from 5 patients and the atrophic lesion of 10 GA donor maculae from 7 patients (expressed as CCR2^+^ cells/500 mm of the atrophic lesion) show a significant reproducible infiltration of CCR2^+^ MPs in all the atrophic lesions of eyes with GA we examined (Fig 1L). To quantify the distribution of MPs in GA lesions more generally, we stained all donor eyes for pan-MP marker IBA-1. IBA-1^+^ cells were highly ramified and located in the inner retina in sections from control eyes – the photoreceptor cell layer was exempt of IBA-1^+^ MPs (Fig 1M). In GA lesions, inner retinal IBA-1^+^ cells were more rounded and less ramified and additional IBA-1^+^ MPs were found in the subretinal space (Fig 1N) as previously described (Combadiere et al, 2007; Gupta et al, 2003). Quantification of the number of IBA-1^+^cells in the inner retina revealed similar numbers in the inner retina for both groups, but a significant increase of subretinal IBA-1^+^cells in the 10 GA donor maculae examined (expressed as Iba^+^ cells/500 mm of the atrophic lesion; Fig 1O).

Taken together, we show for the first time that CCL2 is increased in eyes with GA and that a significant quantity of the subretinal MPs previously described to accumulate in GA (Combadiere et al, 2007; Gupta et al, 2003) are CCR2 positive iMos.

### Subretinal *Cx3cr1*^*−/−*^ MPs over-express CCL2 in age and light-induced inflammation

Deletion of *Cx3cr1* leads to an age-dependent increase of subretinal MPs on a pigmented C57BL/6 (Chinnery et al, 2011; Combadiere et al, 2007) and BALB/C albino background (Chinnery et al, 2011; Combadiere et al, 2007); in *Cx3cr1*^*−/−*^ knockout (Combadiere et al, 2007) and *Cx3cr1*^*GFP/GFP*^ knockin mice (Chinnery et al, 2011; Combadiere et al, 2007). However, *Cx3cr1* deletion in mice does not mimic all aspects of AMD. Indeed, we did not observe drusen formation and RPE atrophy, but these mice do model subretinal inflammation and associated photoreceptor degeneration, two hallmarks of AMD (Gupta et al, 2003).

**Figure 2:**
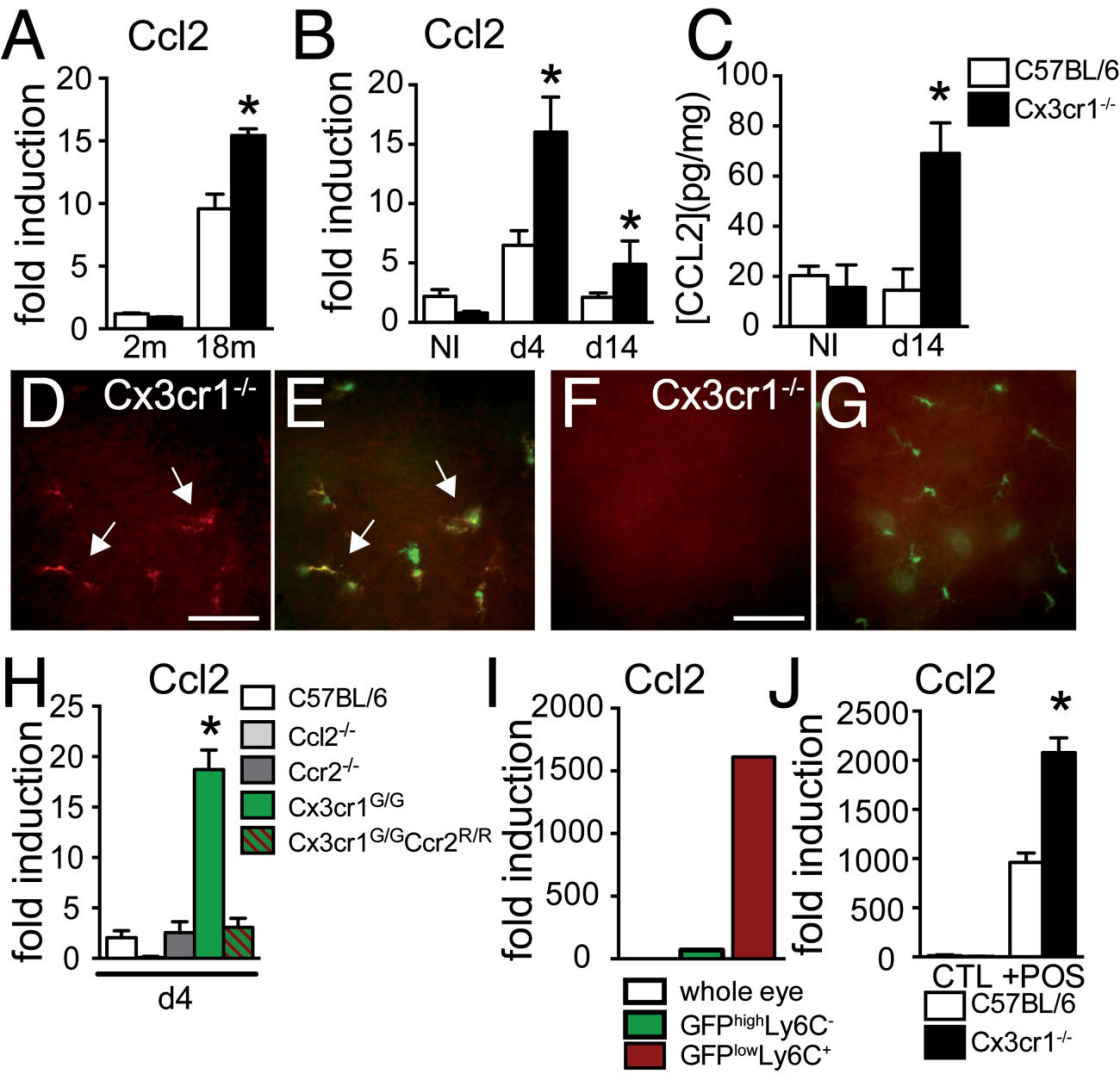
CCL2 expression in age and light-challenged *Cx3cr1^−/−^* mice **A.** Quantitative RT-PCR of *Ccl2* mRNA normalized with *β-actin* mRNA of 2- and 18-month-old C57BL/6 and *Cx3cr1*^*−/−*^ mouse retina (*n* = 4 per group, *two-way Anova, Bonferroni *p* < 0.001).**B.** Quantitative RT-PCR of *Ccl2* mRNA normalized with *β-actin* mRNA of non-injured (NI) and at day 4 and day 14 of the light-challenge model of 2- to 3-month-old C57BL/6 and *Cx3cr1*^*−/−*^ mice (*n* = 5–6 per group, *two-way Anova, Bonferroni *p* < 0,05).**C.** CCL2 ELISA protein quantification of retinal protein extracts from non-injured (NI) and at day 14 (d14) of the light-challenge model of 2- to 3-month-old C57BL/6 and *Cx3cr1*^*−/−*^ mice (expressed as pg/mg total retinal protein; *n* = 4 per group, *two-way Anova, Bonferroni *p* < 0.001).**D–G.** Immunohistochemistry CCL2 (red; arrows), and IBA-1 (green; arrows colocalization yellow) of the subretinal side (D and E) and vitreal aspect (F and G) of a retinal flatmount from a *Cx3cr1*^*−/−*^ mouse at day 14 (d14) of the light-challenge model (representative of 3 independent experiments, immunostaining omitting the primary antibody or performed on *Ccl2*^*−/−*^ mice served as negative controls).**H.** Quantitative RT-PCR of *Ccl2* mRNA normalized with *β-actin* mRNA of 2- to 3-month-old C57BL/6, *Ccl2*^*−/−*^, *Ccr2*^*−/−*^, *Cx3cr1*^*GFP/GFP*^ and *Cx3cr1*^*GFP/GFP*^*Ccr2*^*RFP/RFP*^ mice at day 4 of the light-challenge model (d4) (*n* = 5–6 per group, *two-way Anova, Bonferroni *p* < 0.01).**I.** Quantitative RT-PCR of *Ccl2* mRNA normalized with *S26* of whole-eye-lysats (set as 1) and FACS-sorted GFP^high^Ly6C^neg^ and GFP^low^Ly6C^high^ cells pooled from 8 eyes of PBS perfused *Cx3cr1*^*GFP/GFP*^ mice after 4 days of light challenge.**J.** (J) Quantitative RT-PCR of *Ccl2* mRNA normalized with *S26* mRNA of *Cx3cr1*^*+/+*^ and *Cx3cr1*^*−/−*^ monocyte derived Mφs cultivated for 18 h with or without photoreceptor outer segments (POSs, *n* = 4 per group, two-way Anova Bonferroni *p < 0.001). All values are represented as mean ± SEM. Scale bar D–G = 50 μm; CTL: control; +POS: +photoreceptor outer segments.

We first analysed whether the MP accumulation in *Cx3cr1*^*−/−*^ mice is associated with elevated CCL2 expression. *Ccl2* mRNA expression shows significantly increased expression in 18-month-old *Cx3cr1*^*−/−*^ mice with subretinal MP accumulation compared to age-matched wildtype mice (Fig 2A). This difference is not observed in 2- to 3-month-old *Cx3cr1*^*+/+*^ and *Cx3cr1*^*−/−*^ mice that do not accumulate subretinal MPs. Similarly, in light-challenged 2-month-old *Cx3cr1*^−/−^ that accumulate subretinal MPs (see below), *Ccl2* mRNA are increased at 4 and 14 days (Fig 2B) and CCL2 protein expression is increased at 14 days (Fig 2C) when compared to light-challenged wildtype mice (with little subretinal inflammation) or non-illuminated *Cx3cr1*^*−/−*^ mice. It should be noted that the intensity of the light-challenge model used herein was developed to induce subretinal inflammation and subsequent photoreceptor degeneration in the *Cx3cr1*^*−/−*^ mice but not in *Cx3cr1*^*+/+*^ mice (see below). The light intensity is not strong enough to directly induce photoreceptor apoptosis in pigmented wildtype animals, contrary to classically used light-injury models.

To identify the main CCL2 expressing cells, we performed immunohistochemical analysis of CCL2 (red staining Fig 2D–G) on retinal flatmounts at 14 days in light-challenged *Cx3cr1*^*−/−*^ mice. Robust CCL2 staining in *Cx3cr1*^*−/−*^ mice was observed in IBA-1 positive MPs (Fig 2D CCL2 red staining, 2E IBA-1 green double staining) of the subretinal space. IBA-1^+^ MCs of the inner retina (Fig 1G IBA-1 green staining) did not stain positive for CCL2 (Fig 2F CCL2 red). Similar staining was observed in aged *Cx3cr1*^*−/−*^ mice but never in light-challenged or aged in *Cx3cr1*^*GFP/GFP*^*Ccl2*^*−/−*^ control mice.

To study whether subretinal MPs induce or participate in *Ccl2* mRNA production in retinal inflammation we also compared *Ccl2* mRNA expression 4 days after light-challenge in the eyes of MP accumulating *Cx3cr1*^*GFP/GFP*^ mice and the eyes of *Cx3cr1*^*GFP/GFP*^*Ccr2*^*RFP/RFP*^ mice in which the accumulation is inhibited (see below). The significant *Ccl2* mRNA induction observed in *Cx3cr1*^*GFP/GFP*^ mice (as in *Cx3cr1*^*−/−*^ mice Fig 2B) was completely prevented in *Cx3cr1*^*GFP/GFP*^*Ccr2*^*RFP/RFP*^ mice and comparable to wild type and *Ccr2*^*−/−*^ mice (Fig 2H). Finally, we analysed *Ccl2* mRNA on whole-eye-lysats and MPs sorted by fluorescent activated cell sorted (FACS) of PBS perfused *Cx3cr1*^*GFP/GFP*^ mice after 4 days of light challenge. iMos express high levels of CCR2 and Ly6C and low levels of CX3CR1, while MCs and resident macrophages express low levels of CCR2 and Ly6C and high levels of CX3CR1 (Ransohoff & Cardona, 2010; Wynn et al, 2013). *Ccl2* mRNA was greatly enriched in sorted GFP^low^Ly6C^high^ cells (pooled from 8 eyes) when compared to whole-eye-lysats, confirming that *Cx3cr1*-deficient Ly6C^high^CCR2^+^ MPs strongly express CCL2 in the model (Fig 2I). The expression in sorted GFP^high^Ly6C^neg^ corresponding to resident Mφs and MCs was found to be much lower.

We have previously shown that subretinal MPs engulf photoreceptor outer segments (POSs) (Combadiere et al, 2007) known to be rich in phosphatidylserine (Miljanich et al, 1981). Phagocytosis of substrates rich in phosphatidylserine, such as apoptotic bodies and POS, has been shown to only induce small amounts of pro-inflammatory mediators and to promote the resolution of inflammation in wild type macrophages (Huynh et al, 2002). To evaluate whether CX3CR1 influences the level of CCL2 expression in Mφs in the subretinal microenvironment, *Cx3cr1*^*+/+*^ and *Cx3cr1*^*−/−*^ macrophages derived from purified bone marrow monocytes (bMo) were incubated with POS prepared from porcine retina. We show that *Ccl2* mRNA expression (Fig 2J) is comparable in control *Cx3cr1*^*+/+*^ and *Cx3cr1*^*−/−*^ Mφs at 18 h of culture. Interestingly, when the Mφs were derived in the presence of POS, *Ccl2* mRNA induction in *Cx3cr1*^*−/−*^ Mφs was significantly stronger compared to *Cx3cr1*^*+/+*^ Mφs (Fig 2J) suggesting that CX3CR1 signalling helps to repress CCL2 secretion in subretinal wild type Mφs.

Taken together, several lines of evidence suggest that subretinal *Cx3cr1*^*−/−*^ MPs are the main source of CCL2 release in subretinal inflammation observed in *Cx3cr1*^*−/−*^ mice. We show that the absence of CX3CR1/CX3CL1 signalling in the subretinal environment fails to repress CCL2 induction in infiltrating Mos. As a result, CCL2 is overexpressed in subretinal inflammation in *Cx3cr1*^*−/−*^ mice.

### CCL2 deletion protects *Cx3cr1*^*−/−*^ mice from age and light-induced subretinal MP accumulation

**Figure 3:**
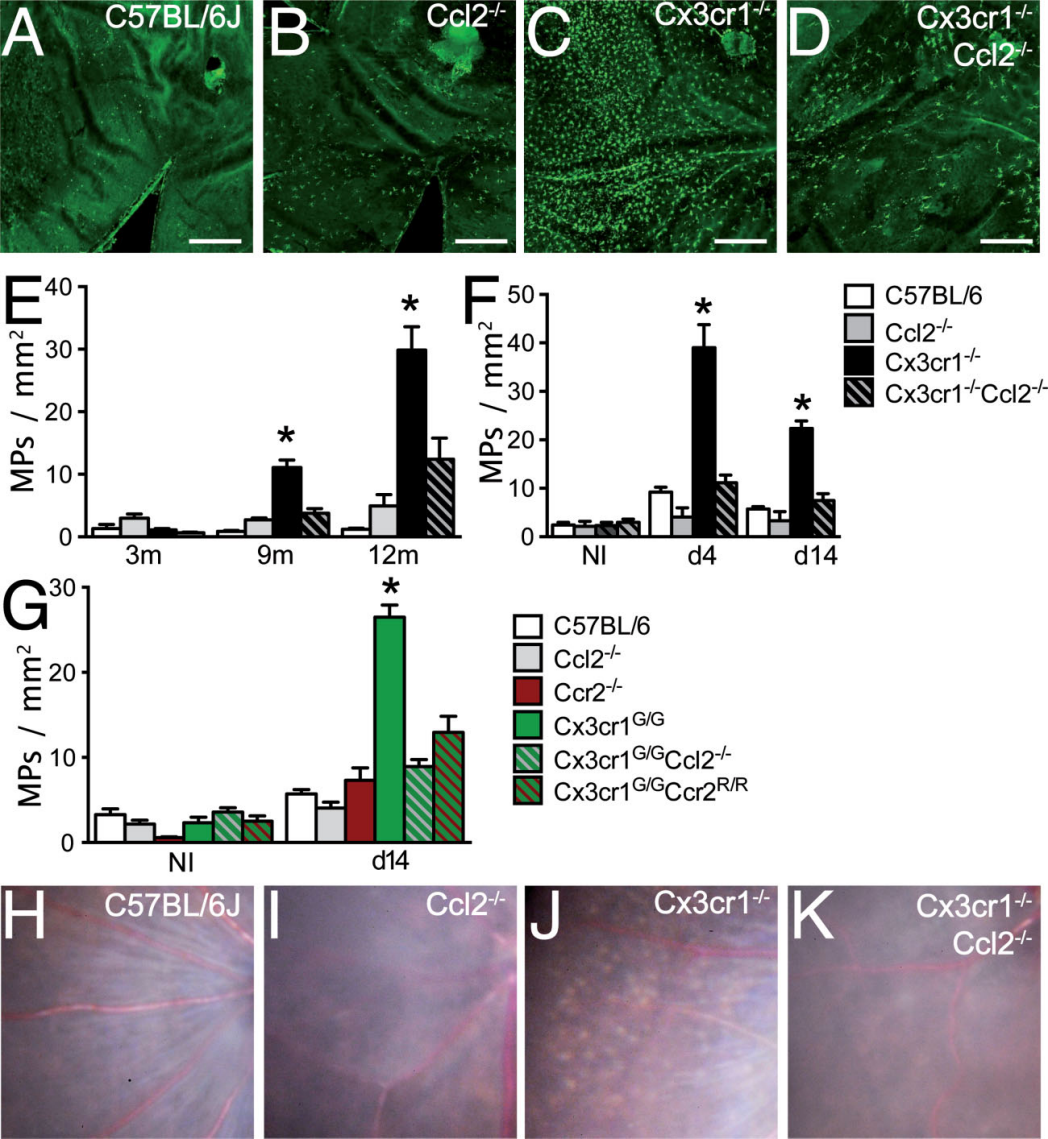
CCL2 mediates subretinal MP accumulation in age and light-challenged *Cx3cr1^−/−^* mice **A–D.** 12 month-old IBA-1 stained RPE-flatmounts of C57BL/6 (A), *Ccl2*^*−/−*^ (B), *Cx3cr1*^*−/−*^ (C) and *Cx3cr1*^*−/−*^*Ccl2*^*−/−*^ (D).**E.** Quantification of subretinal MPs/mm^2^ at 3, 9 and 12 months (*n* = 6 per group, *two-way Anova Bonferroni *p* < 0,001).**F.** Quantification of subretinal MPs/mm^2^ non-injured and at day 4 and 14 of the light-challenge model of 2- to 3-month-old C57BL/6J, *Ccl2*^*−/−*^, *Cx3cr1*^*−/−*^ and *Cx3cr1*^*−/−*^*Ccl2*^*−/−*^ mice (*n* = 6–9 per group, *two-way Anova Bonferroni *p* < 0.001).**G.** Quantification of IBA-1 positive subretinal MPs/mm^2^ in non-injured and at day 14 of the light-challenge model of 2- to 3-month-old C57BL/6J, Ccl2^*−/−*^, *CCR2*^*−/−*^, *Cx3cr1*^*GFP/GFP*^, *Cx3cr1*^*GFP/GFP*^*Ccl2*^*−/−*^ and *Cx3cr1*^*GFP/GFP*^*Ccr2*^*RFP/RFP*^ mice (*n* = 6–9 per group, *two-way Anova Bonferroni *p* < 0.001).**H–K.** Representative fundoscopic photographs of 12-month-old C57BL/6 (H), *Ccl2*^*−/−*^ (I), *Cx3cr1*^*−/−*^ (J) and *Cx3cr1*^*−/−*^*Ccl2*^*−/−*^ (K) (*n* > 8 per group). All values are represented as mean ± SEM. Scale A–D = 100 μm.

We have previously shown that *Cx3cr1*^*−/−*^ mice spontaneously accumulate subretinal MPs with age and light exposure (Combadiere et al, 2007; Raoul et al, 2008b). To analyse the influence of CCL2 on subretinal MP accumulation we generated *Cx3cr1*^*−/−*^*Ccl2*^*−/−*^ mice, *Cx3cr1*^*GFP/GFP*^*Ccl2*^*−/−*^ mice, and *Cx3cr1*^*GFP/GFP*^*Ccr2*^*RFP/RFP*^ knockin mice. First, we evaluated the number of subretinal MPs on 12 month-old IBA-1 stained RPE-flatmounts of C57BL/6 (Fig 3A), *Ccl2*^*−/−*^ (Fig 3B), *Cx3cr1*^*−/−*^ (Fig 3C), and *Cx3cr1*^*−/−*^*Ccl2*^*−/−*^ (Fig 3D). *Cx3cr1*^*−/−*^ mice accumulate numerous subretinal MPs at this age (Fig 3C) compared to C57BL/6 mice (Fig 3A) kept in the same conditions, as previously described (Combadiere et al, 2007). In 12-month-old *Ccl2*^*−/−*^ mice (Fig 3B, 18 month-old in Supporting Information data) a slight increase of subretinal MPs compared to C57BL/6 mice can be observed as previously reported in much older *Ccl2*^*−/−*^ mice (20 months and older) (Luhmann et al, 2009). Importantly, the population of subretinal MP in *Cx3cr1*^*−/−*^*Ccl2*^*−/−*^ mice was strongly reduced compared to *Cx3cr1*^*−/−*^ mice of the same age (Fig 3D). Quantification of subretinal MPs at 3, 9 and 12 months shows a strong increase in numbers in *Cx3cr1*^*−/−*^ mice at 12 months that is significantly reduced in *Cx3cr1*^*−/−*^*Ccl2*^*−/−*^ mice at 9 and 12 months (Fig 3E). In light-challenged *Cx3cr1*^*−/−*^ mice, subretinal MPs strongly increased compared to controls, peaking at day four and the accumulation was significantly reduced in *Cx3cr1*^*−/−*^*Ccl2*^*−/−*^ mice at 4 and 14 days (Fig 3F). Similarly, light-challenged *Cx3cr1*^*GFP/GFP*^ mice significantly accumulated subretinal MPs compared to control mice; the accumulation was significantly inhibited in *Cx3cr1*^*GFP/GFP*^*Ccl2*^*−/−*^ mice, and *Cx3cr1*^*GFP/GFP*^*Ccr2*^*RFP/RFP*^ knockin mice at day 14 (Fig 3G). In summary, subretinal MP accumulation observed in *Cx3cr1* deficient mice was significantly inhibited in three distinct, independently bred, double knockout strains (*Cx3cr1*^*−/−*^*Ccl2*^*−/−*^ mice, *Cx3cr1*^*GFP/GFP*^*Ccl2*^*−/−*^ mice and *Cx3cr1*^*GFP/GFP*^*Ccr2*^*RFP/RFP*^ knockin mice).

We have previously shown that some of the subretinal MPs in aged *Cx3cr1*^*−/−*^ mice engulf lipid-rich POSs, accumulate lipid intracellularly and become visible as pseudodrusen in funduscopy (Combadiere et al, 2007). Eye examination of 12-month-old C57BL/6 (Fig 3H), *Ccl2*^*−/−*^ (Fig 3I), *Cx3cr1*^*−/−*^ (Fig 3J), and *Cx3cr1*^*−/−*^*Ccl2*^*−/−*^ (Fig 3K) revealed these characteristic yellow-white deposits in *Cx3cr1*^−/−^ mice (Fig 3J) but not in the other mouse strains including the *Cx3cr1*^*−/−*^*Ccl2*^*−/−*^ mice, confirming that CCL2 deletion prevents the appearance of subretinal MP-associated pseudodrusen in *Cx3cr1*^−/−^ mice. None of the three generated double knockout mouse strains contained the rd8 mutation or presented signs of the early onset AMD-like features previously described (Tuo et al, 2007).

In summary, using three independently bred double knockout strains, we show that deletion of CCL2/CCR2 signalling strongly inhibits subretinal MP accumulation in *Cx3cr1* deficiency. Consequently, the number of the fundoscopically visible pseudodrusen that constitute lipid bloated subretinal MPs are equally reduced.

### CCL2 deficiency protects *Cx3cr1*^*−/−*^ mice from age- and light-induced photoreceptor degeneration

**Figure 4:**
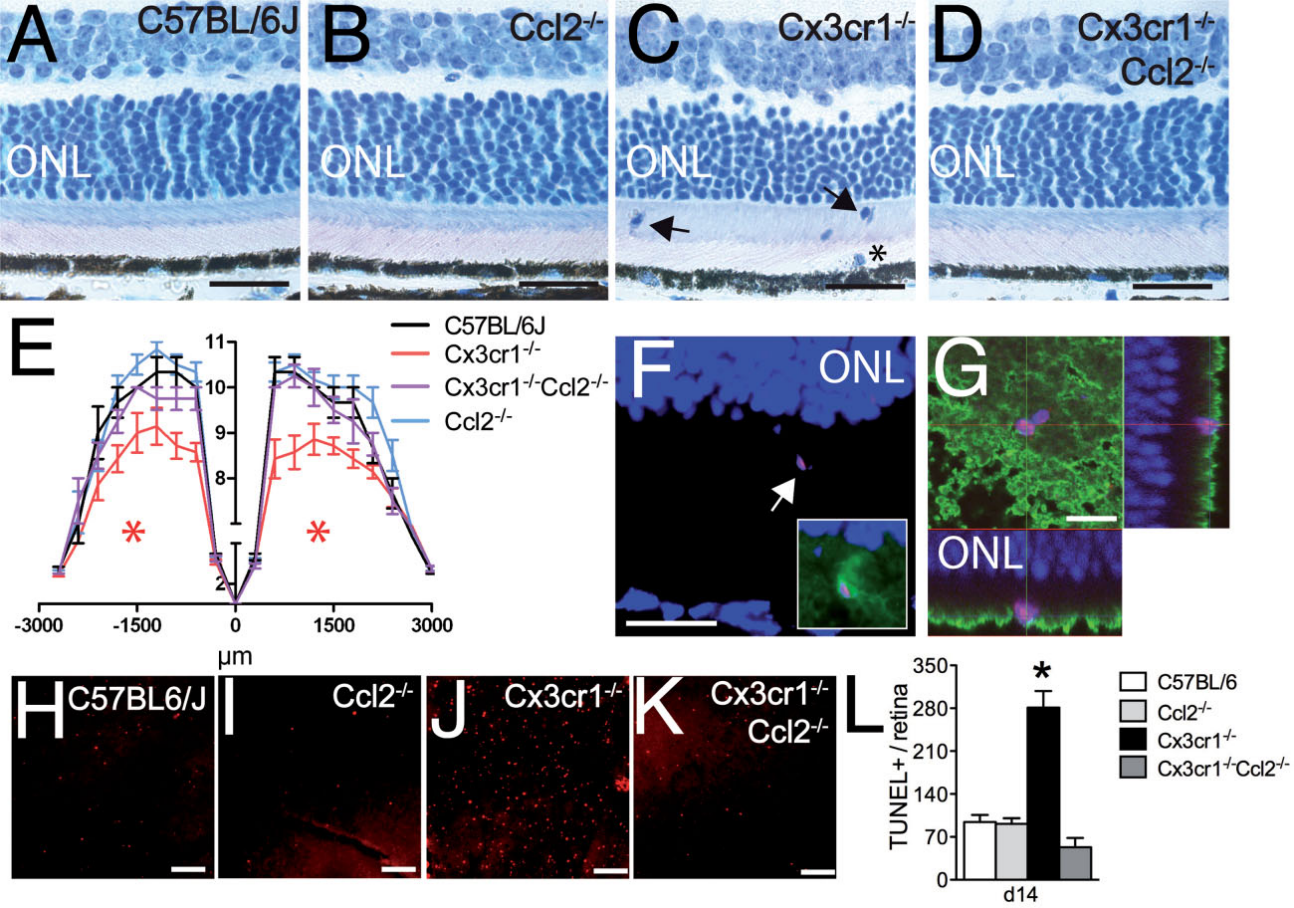
CCL2 deficiency protects *Cx3cr1^−/−^* mice from age and light-induced photoreceptor degeneration **A–D.** Micrographs, taken 1000 μm from the optic nerve of 12-month-old C57BL/6J (A), *Ccl2*^*−/−*^ (B), *Cx3cr1*^*−/−*^ (C, arrows: photoreceptor nuclei; star: nucleus of a subretinal macrophage) and *Cx3cr1*^*−/−*^*Ccl2*^*−/−*^ (D).**E–G.** (E) Photoreceptor nucleus rows at increasing distances (−3000 μm: inferior pole, +3000 μm: superior pole) from the optic nerve (0 μm) in 12-month-old C57BL/6J, *Ccl2*^*−/−*^, *Cx3cr1*^*−/−*^ and *Cx3cr1*^*−/−*^*Ccl2*^*−/−*^ mice (*n* = 4–7, the area under the curve in *Cx3cr1*^*−/−*^ mice tested significantly different from the other strains *one-way Anova Bonferroni *p* < 0.05). Hoechst (blue staining), TUNEL (red staining), rhodopsin (green staining) labelling of *Cx3cr1*^*−/−*^ mice at d14 of the light-challenge model in sections (F inset showing rhodopsin double labelling) and confocal microscopy of flatmounts with z-stack projections (G).**H–K.** TUNEL stained retinal flatmounts of d14 light-challenged C57BL/6 (H), *Ccl2*^*−/−*^ (I), *Cx3cr1*^*−/−*^ (J) and *Cx3cr1*^*−/−*^*Ccl2*^*−/−*^(K) mice.**L.** Quantification of TUNEL positive cells per retina (*n* = 4–6 per group, *one-way Anova Bonferroni *p* < 0.01). All values are represented as mean ± SEM. ONL: outer nuclear layer. Scale bars: A–D and G = 50 μm; F and H–K = 100 μm.

Subretinal accumulation of *Cx3cr1* deficient MPs is associated with retinal degeneration (Chen et al, 2013; Chinnery et al, 2011) and more precisely photoreceptor degeneration (Combadiere et al, 2007). Similarly, in the brain *Cx3cr1* deficient MPs display increased neurotoxicity compared to CX3CR1 expressing MPs (Cardona et al, 2006). To evaluate the influence of *Ccl2* deficiency on photoreceptor degeneration in *Cx3cr1*^*−/−*^ mice, we evaluated the photoreceptor cell layer on histological sections of 12 month-old mice. Micrographs, taken at equal distance from the optic nerve show the regular structure of the outer nuclear layer (ONL, that contains the photoreceptor nuclei) clearly delimited by the outer plexiform layer in C57BL/6 (Fig 4A), *Ccl2*^*−/−*^ (Fig 4B), and *Cx3cr1*^*−/−*^*Ccl2*^*−/−*^ mice (Fig 4D). In contrast, *Cx3cr1*^*−/−*^ mice (Fig 4C) at equal distance from the optic nerve, display a thinned ONL, an irregular border on the outer plexiform layer and displaced photoreceptor nuclei in the inner segments of the photoreceptor (arrow) characteristic for the macrophage-associated photoreceptor cell death described in retinal detachment (Hisatomi et al, 2003). Photoreceptor cell nuclei were quantified in the mouse strains by counting photoreceptor nucleus rows at increasing distances from the optic nerve (0 μm) and calculated as the area under the curve. While 12-month-old *Cx3cr1*^*−/−*^ mice displayed a significant loss of photoreceptors compared to age matched C57BL/6 mice (not observed at 3 months of age, Supporting Information data), 12-month-old *Cx3cr1*^*−/−*^*Ccl2*^*−/−*^ mice were completely protected against *Cx3cr1* deficiency dependent degeneration (Fig 4E).

As shown above, light-challenge triggers MP accumulation in 3-month-old *Cx3cr1*^*−/−*^ mice similar to the spontaneous accumulation observed with age (Fig 3). To evaluate whether acute MP accumulation induces photoreceptor cell death, we performed TUNEL-staining on light-challenged retina. Displaced TUNEL positive nuclei (Fig 4F red staining) that also stain positive for rhodopsin (Fig 4F inset, green staining) can be observed in the photoreceptor segments of light-challenged *Cx3cr1*^*−/−*^ mice at day 14 in sections (Fig 4F) and flatmounts (Fig 4G). Similarly, it has been shown that these displaced photoreceptor nuclei represent dying TUNEL positive photoreceptors in retinal detachment, where subretinal macrophage accumulation and photoreceptor degeneration occurs (Hisatomi et al, 2003). To quantify photoreceptor apoptosis in light-challenged mice we prepared TUNEL stained retinal flatmounts of C57BL/6 (Fig 4H), *Ccl2*^*−/−*^ (Fig 4I), *Cx3cr1*^*−/−*^ (Fig 4J), and *Cx3cr1*^*−/−*^*Ccl2*^*−/−*^ (Fig 4K) mice. Flatmounts from *Cx3cr1*^*−/−*^ mice displayed TUNEL positive cells to a much greater extent than C57BL/6, *Ccl2*^*−/−*^ and *Cx3cr1*^*−/−*^*Ccl2*^*−/−*^ mice. Quantification of TUNEL positive cells per retina shows a significant CCL2 dependent increase in *Cx3cr1*^*−/−*^ mice, as *Cx3cr1*^*−/−*^*Ccl2*^*−/−*^ mice were not different from C57BL/6 and *Ccl2*^*−/−*^ mice (Fig 4L).

Taken together, the photoreceptor degeneration observed in aged and light-challenged *Cx3cr1*^*−/−*^ mice is greatly inhibited in *Cx3cr1*^*−/−*^*Ccl2*^*−/−*^ mice that are significantly protected from subretinal inflammation.

### Subretinal MP accumulation in *Cx3cr1*^*−/−*^ mice is dependent on CCL2 mediated monocyte recruitment

Blood monocytes can be divided into two subsets (Geissmann et al, 2010; Swirski et al, 2007; Tsou et al, 2007): the CX3CR1^low^CCR2^+^ monocytes, the blood-borne precursors of inflammatory macrophages and inflammatory DCs, and the CX3CR1^high^CCR2^−^ monocytes that patrol the vasculature (Geissmann et al, 2003; Tsou et al, 2007). Infiltrating CCR2^+^ monocytes downregulate *Ccr2* mRNA (Wong et al, 1997) and can give rise to classically- and alternatively-activated macrophages (Arnold et al, 2007). Brain MCs and tissue resident Mφs do not express CCR2, but do show high levels of CX3CR1 (Gautier et al, 2012; Geissmann et al, 2003; Saederup et al, 2010).

**Figure 5:**
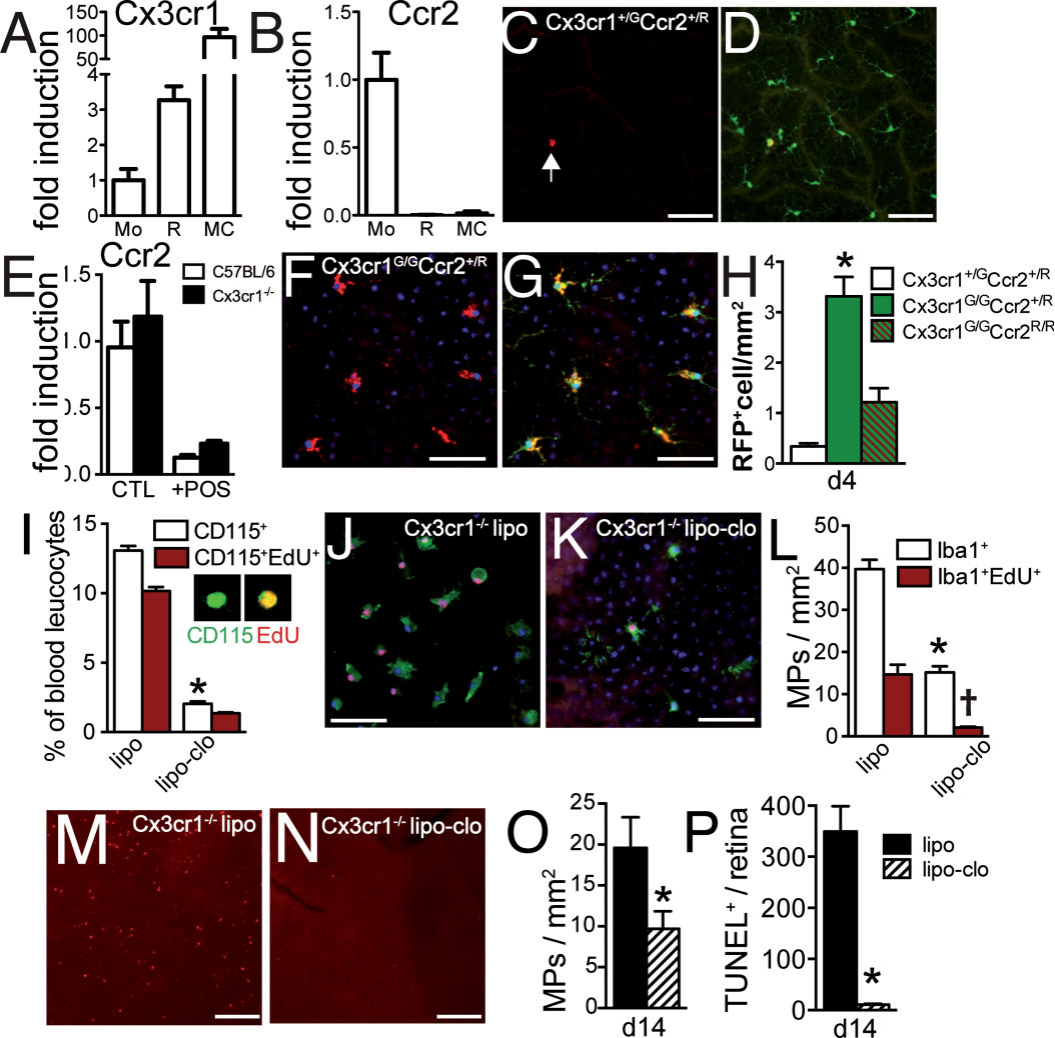
Subretinal MP accumulation and photoreceptor degeneration in *Cx3cr1^−/−^* mice is dependent on CCL2 mediated monocyte recruitment **A, B.** RT-PCR of relative (A) *Cx3cr1* and (B) *Ccr2* mRNA expression normalized with *S26* mRNA in C57BL/6 blood monocytes (Mo), retina (R) and retinal microglial cells (MC) (*n* = 3 independent cell preparations, all groups significantly different from each other for *Cx3cr1* by one-way Anova Bonferoni *p* < 0.05; Mo significantly different from MC and R for *Ccr2* by two-way Anova Bonferoni *p* < 0.001).**C, D.** Confocal microscopy of (C) RFP fluorescence and (D) merged RFP and GFP fluorescence of the outer plexiform layer of non-injured *Cx3cr1*^*+/GFP*^*Ccr2*^*+/RFP*^*mice*.**E.** Quantitative RT-PCR of *Ccr2* mRNA normalized with *S26* mRNA of *Cx3cr1*^*+/+*^ and *Cx3cr1*^*−/−*^ freshly prepared monocytes and monocyte derived Mφs cultivated for 18 h in direct contact with photoreceptor outer segments (POS) (*n* = 4 per group, two-way Anova Bonferroni *p* < 0.001 significant difference in monocytes compared to Mφs; no difference between genotypes).**F, G.** (F) RFP fluorescence and (G) merged RFP and GFP fluorescence of Hoechst stained chroidal/RPE flatmount after 4 days of light-challenge of *Cx3cr1*^*GFP/GFP*^*Ccr2*^*+/RFP*^ mice.**H.** Quantification of RFP positive subretinal cells after 4 days of light-challenge in *Cx3cr1*^*+/GFP*^*Ccr2*^*+/RFP*^, *Cx3cr1*^*GFP/GFP*^*Ccr2*^*+/RFP*^ and *Cx3cr1*^*GFP/GFP*^*Ccr2*^*RFP/RFP*^ mice (*n* = 4 per group, *one-way Anova Bonferroni *p* < 0.01).**I.** Quantification of the percentage of CD115^+^ (inset green staining) and CD115^+^EdU^+^ cells (inset red/green staining) of all Hoechst positive leukocytes of blood smears from day 4 light-challenged *Cx3cr1*^*−/−*^ mice that all received three daily intraperitoneal EdU injections and one daily intravenous injection of either control or clodronate liposome (*n* = 4 per group, two-way Anova Bonferroni *p* < 0.001 significant difference in the number of CD115^+^ and CD115^+^EdU^+^ cells in clodronate liposome treated animals compared to controls).**J, K.** IBA-1 (green) EdU (red) double labelled chroidal/RPE flatmount of intraperitoneally EdU-injected light-challenged *Cx3cr1*^*−/−*^ mice receiving control liposome injections (J) or clodronate liposome injections (K).**L.** Quantification of subretinal IBA-1^*+*^ and IBA-1^+^EdU^+^ cells of control liposome and clodronate liposomes treated 4 days light-challenged *Cx3cr1*^*−/−*^ mice (*n* = 4 mice per group, two-way Anova Bonferroni *p* < 0.001 significant difference in the numbers of subretinal *IBA-1^+^ MPs and †IBA-1^+^EdU^+^ MPs between clodronate liposome and control liposome treated mice).**M, N.** TUNEL stained retinal-flatmounts of control liposome (M) and clodronate liposome (N) treated light-challenged *Cx3cr1*^*−/−*^ at d14.**O, P.** O) Quantification of IBA-1 positive subretinal MPs/mm^2^ (*n* = 4–7 per group, *Mann–Whitney *p* = 0.03) and (P) TUNEL positive photoreceptor nuclei in control liposome and clodronate liposome treated light-challenged *Cx3cr1*^*−/−*^ at d14 (*n* = 4–7 * Mann–Whitney *p* < 0.0001). All values are represented as mean ± SEM. R: Retina; MC: microglial cells; Mo: monocytes; POS: photoreceptor outer segments; lipo: empty control liposomes; lipo-clo: clodronate liposomes. Scale bars = 50 μm.

To evaluate *Cx3cr1* and *Ccr2* expression in the retina and MPs, we compared mRNA expression on whole tissue and isolated cells by RT-PCR. *Cx3cr1* mRNA was expressed by circulating monocytes, its expression was stronger in the retina and greatly concentrated in purified retinal MCs (Fig 5A). In contrast, *Ccr2* mRNA was expressed in circulating monocytes, but not detectable in retinal extracts and barely detectable in purified retinal MCs (Fig 5B) in control adult wildtype mice. Accordingly, retinal flatmounts from non-injured *Cx3cr1*^*GFP/+*^*Ccr2*^*RFP/+*^ mice, expressing the red fluorescent protein under the *Ccr2* promoter and the green fluorescent protein under the *Cx3cr1* promoter (Saederup et al, 2010), confirmed that CCR2 expressing RFP^+^ cells (Fig 5C arrow) are very rare in the healthy retina and morphologically similar to monocytes (small and round). As suggested by the RT-PCR, resting retinal MCs did not express *Ccr2* promoter controlled RFP but expressed high levels of *Cx3cr1* promoter controlled GFP as previously described (Fig 5D) (Combadiere et al, 2007). The RPE, or any other retinal cell, did not express RFP or GFP and the photoreceptor cell layer and subretinal space was devoid of any significant amount of RFP^+^ or GFP^+^ cells. We next compared the level of *Ccr2* mRNA expression in *Cx3cr1*^*+/+*^ and *Cx3cr1*^*−/−*^ freshly prepared monocytes and after *in vitro* differentiation into macrophages in the presence of POSs to simulate macrophage differentiation in the subretinal space (Fig 5E). *Ccr2* mRNA did not differ between genotypes, but decreased with macrophage differentiation as previously described (Wong et al, 1997). Our results suggest that Ccr2 promoter controlled RFP expression, combined with the dendritic morphological feature, allows for the identification of infiltrating Mo-derived MPs as compared to migrating MCs for some time after infiltration as described in the brain (Mizutani et al, 2011).

To evaluate whether CCR2^+^ monocytes are recruited to the subretinal space in *Cx3cr1* deficiency, we evaluated RFP^+^ cells 4 days into a light-challenge. Ramified subretinal RFP^+^ cells (Fig 5F) that were also positive for GFP (Fig 5G) were significantly more numerous in *Cx3cr1*^*GFP/GFP*^*Ccr2*^*+/RFP*^ mice compared to heterozygote control mice (Fig 5H). More importantly, significantly diminished subretinal RFP^+^ cells in *Cx3cr1*^*GFP/GFP*^*Ccr2*^*RFP/RFP*^ mice confirm that recruitment is greatly dependent on the CCL2/CCR2 axis (Fig 5H). Our data suggests that RFP^+^ monocytes, recruited from the bloodstream, differentiate into ramified iMφ in light-challenged *Cx3cr1* deleted mice. However, as infiltrating Mos quickly downregulate CCR2, subretinal RFP^+^ cells in *Cx3cr1*^*GFP/GFP*^*Ccr2*^*+/RFP*^ mice probably only represent a fraction of the monocyte-derived MPs in the subretinal space.

To evaluate the participation of monocyte recruitment from the bloodstream *versus* resident Mφs and MCs in subretinal MP accumulation we injected light-challenged *Cx3cr1*^*−/−*^mice from day one to day four with the traceable nucleotide EdU to permanently mark the quickly dividing monocytes and with daily intravenous injections of either empty control liposomes or clodronate containing liposomes that eliminate circulating monocytes within 24 h (Sunderkotter et al, 2004). We determined in preliminary experiments that three days of three intraperitoneal EdU injections/day marks the majority of circulating monocytes and that subretinal MP recruitment in light-challenged *Cx3cr1*^*−/−*^ mice mainly appears at day three and four. Analysis of EdU CD115 double stained blood smears at day four revealed that three daily EdU injections rendered 76% of circulating CD115^+^ monocytes EdU positive in control liposome injected mice (Fig 5I). Clodronate liposomes significantly lowered circulating monocytes by 84% (Fig 5I). In the eye, IBA-1 (green) and EdU (red) double labelled retinal and RPE-flatmounts of light-challenged control-liposome-treated *Cx3cr1*^*−/−*^ mice (Fig 5J) revealed numerous IBA-1^+^EdU^+^ MPs among the IBA-1^+^ subretinal MPs. In contrast, subretinal IBA-1^+^ cells, and more particularly IBA-1^+^EdU^+^ cells, were much less frequently observed in light-challenged clodronate-liposome-treated *Cx3cr1*^*−/−*^ mice (Fig 5K). Quantification of IBA-1^+^ and IBA-1^+^EdU^+^ cells shows that ∼37% of IBA-1^+^ subretinal MPs in the control liposome-treated group were IBA-1^+^EdU^+^ and that circulating monocyte depletion (clodronate liposomes) significantly inhibited subretinal IBA-1^*+*^MP accumulation by ∼60% (Fig 5L). More importantly, it inhibited IBA-1^+^EdU^+^ accumulation by ∼85% (Fig 5L). IBA-1 EdU double labelled retinal and RPE-flatmounts of light-challenged Cx3cr1^−/−^ mice injected intravitreally at day two with EdU marked dividing corneal epithelial cells (internal positive control) did not reveal IBA-1^+^EdU^+^ MPs (Supporting Information data), which suggests that local MP proliferation does not play a major role in subretinal MP accumulation in light-challenged *Cx3cr1*^*−/−*^ mice. Taken together, the significant amount of subretinal IBA-1^+^EdU^+^ positivity in subretinal MPs in control-liposome treated mice, in conjunction with their strong decrease in monocyte depleted animals and the absence of IBA-1^+^EdU^+^ positive cells after local EdU administration all suggest that a significant proportion of subretinal MPs are derived from infiltrating monocytes. Subretinal IBA-1^+^ EdU^−^MPs likely represent MCs and resident Mϕs that were present before EdU administration and migrated to the subretinal space after the light-challenge.

To evaluate the influence of infiltrating monocytes on photoreceptor apoptosis in light-challenged *Cx3cr1*^*−/−*^ mice we prepared TUNEL stained retinal flatmounts at day 14 of *Cx3cr1*^*−/−*^ mice that had received daily intravenous injections for 4 days during light exposure of either empty control liposomes (Fig 5M) or clodronate containing liposomes (Fig 5N). Flatmounts from control liposome treated *Cx3cr1*^*−/−*^ mice displayed TUNEL positive cells to a much greater extent than clodronate liposome-treated mice. Quantification of IBA-1^+^ subretinal MPs at day 14 confirmed that circulating monocyte depletion significantly inhibited subretinal MP accumulation in light-challenged *Cx3cr1*^*−/−*^ mice, as observed at day four (Fig 5O) and nearly completely prevented photoreceptor apoptosis evaluated on TUNEL stained retinal flatmounts in this model (Fig 5P).

Taken together, our experiments show that subretinal MPs observed in Cx3cr1^−/−^ mice constitute a significant degree of monocyte-derived MPs recruited from the bloodstream and seem to have an important role in the induction of photoreceptor cell death.

### Photoreceptor toxicity of wildtype and *Cx3cr1*^*−/−*^ monocytes and microglial cells on retinal explants

**Figure 6:**
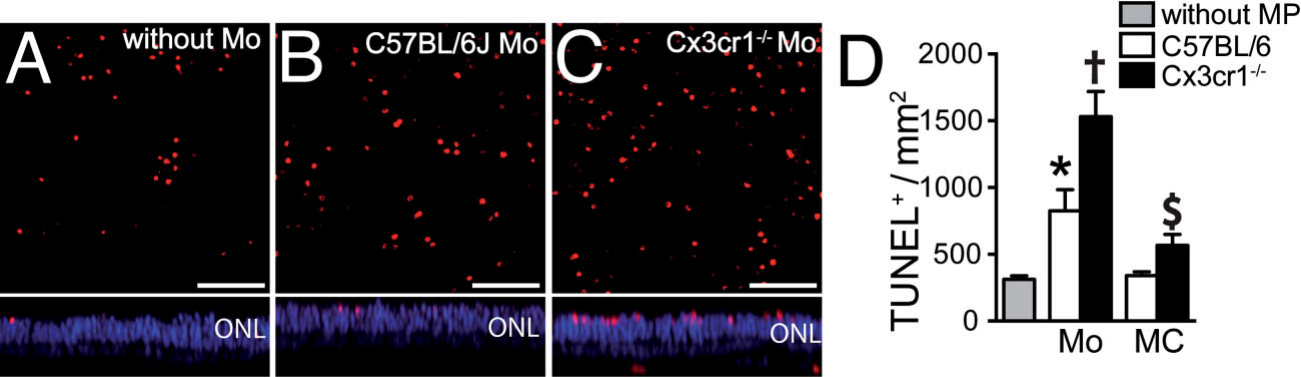
Photoreceptor toxicity of wildtype and *Cx3cr1^−/−^* monocytes and microglial cells on retinal explants **A–C.** Confocal microscopy of flatmounts with z-stack projections (Hoechst in blue) of TUNEL (red) stained retinal-flatmounts of cultured for 18 h without MPs (A), in contact with C57BL/6 monocytes (B) and *Cx3cr1*^*−/−*^ monocytes (C).**D.** Quantification of TUNEL^+^nuclei/mm^2^ in the ONL of the different groups (*n* = 8 per group, one-way Anova Bonferroni *significant difference between explants without MPs and C57BL/6 monocytes *p* < 0.01; † significant difference between explants with C57BL/6 monocytes and *Cx3cr1*^*−/−*^ monocytes *p* < 0.01; $ significant difference between *Cx3cr1*^*−/−*^ monocytes and *Cx3cr1*^*−/−*^ MC *p* < 0.001). All values are represented as mean ± SEM. Mo: monocytes; MC: microglial cells. Scale bar = 50 μm.

MCs are recognized to have neuroprotective functions (Tremblay et al, 2011), while the sustained presence of iMos can be detrimental in neurodegenerative conditions such as multiple sclerosis and stroke (Conductier et al, 2010; Ransohoff, 2009). It is not clear to what extent activated MCs will become neurotoxic in pathological conditions. In our experiments the inhibition of monocyte recruitment to the subretinal space in *Cx3cr1*^*−/−*^ mice (*Ccl2, Ccr2* deletion and monocyte depletion, Figs 3 and 5) resulted in a 50% reduction of subretinal MP accumulation, but in a much stronger reduction of photoreceptor cell death (Figs 4 and 5). These results might suggest that the photoreceptor degeneration observed in *Cx3cr1*^*−/−*^ mice is mainly due to the toxicity of the infiltrating monocytes, and only to a minor extent to the accumulation of resident Mϕ and MCs that still accumulate to some extent in *Ccl2/Ccr2* deficient and monocyte-depleted *Cx3cr1*^*−/−*^ mice. To analyse the respective toxicity of wildtype and *Cx3cr1*^*−/−*^ Mos and MCs on retinal explants, 100,000 C57BL/6 and *Cx3cr1*^*−/−*^ Mos (prepared from bone marrow) and MCs (prepared from brain) adhering to polycarbonate filters floating on DMEM were co-cultured with C57BL/6 retinal explants for 18 hours (with the photoreceptors facing the adherent MPs). Photoreceptor apoptosis was analysed by TUNEL staining of the retinal explants. TUNEL^+^ nuclei in the photoreceptor cell layer of retinal explants cultured without Mos (Fig 6A), with C57BL/6 Mos (Fig 6B), and with *Cx3cr1*^*−/−*^ Mos (Fig 6C) were more numerous in the presence of Mos and particularly in *Cx3cr1*^*−/−*^ Mos. Quantification of TUNEL^+^ nuclei/mm^2^ on 8 explants per group revealed a significant increase in TUNEL^+^ photoreceptors in C57BL/6 Mos and a further significant increase in *Cx3cr1*^*−/−*^ Mos compared to retinal explants cultured without MPs. Interestingly, C57BL/6 and *Cx3cr1*^*−/−*^ MCs induced little supplementary photoreceptor apoptosis compared to retinal explants cultured without MPs and the number of apoptotic photoreceptors was significantly inferior in retinal explants co-cultured with *Cx3cr1*^*−/−*^ MCs compared to *Cx3cr1*^*−/−*^ Mos. These results suggest that Mos and particularly *Cx3cr1*^*−/−*^ Mos display photoreceptor toxicity compared to MCs and might explain the marked neuroprotective effect of monocyte depletion in light-challenged *Cx3cr1*^*−/−*^ mice.

### Blocking CCR2/CCL2 axis protects *Cx3cr1*^*−/−*^ mice from MP accumulation and photoreceptor degeneration

**Figure 7:**
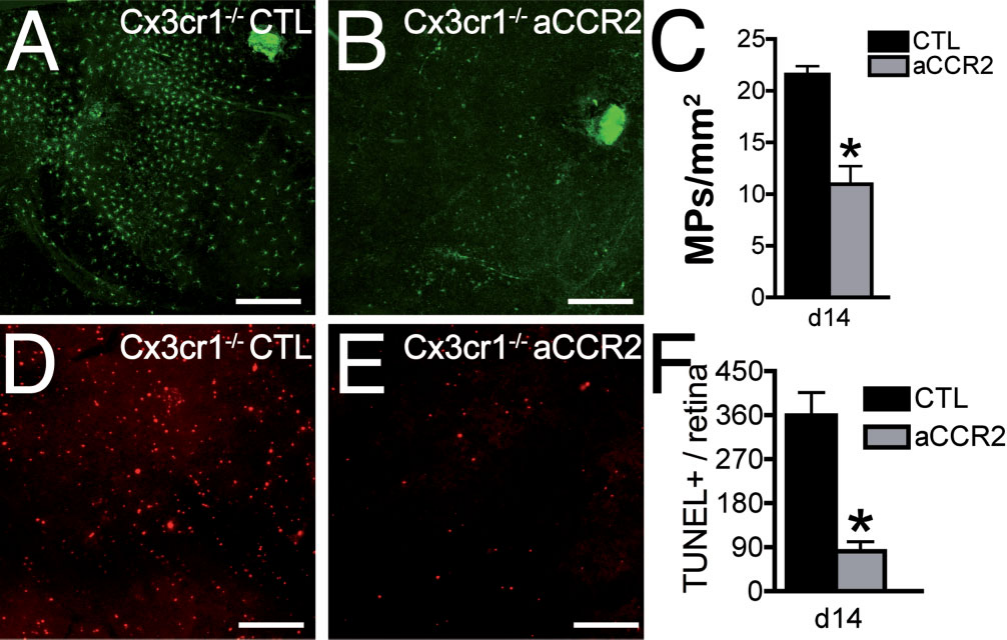
Blocking CCR2/CCL2 axis protects *Cx3cr1^−/−^* mice from MP accumulation and photoreceptor degeneration **A, B.** IBA-1 stained RPE-flatmounts of PBS (A) and CCR2 inhibitor RS 102895 (B) treated light-challenged *Cx3cr1*^*−/−*^ mice at d14.**C–E.** C) Quantification of IBA-1 positive subretinal MPs/mm^2^ (*n* = 6–10 per group, *Mann–Whitney *p* = 0.01). TUNEL stained retinal-flatmounts of PBS (D) and CCR2 inhibitor (E) treated light-challenged *Cx3cr1*^*−/−*^ mice at d14.**F.** Quantification of TUNEL positive photoreceptor nuclei per retina (*n* = 4 per group, *Mann–Whitney *p* = 0.028). All values are represented as mean ± SEM. Scale bar = 100 μm.

We have shown that elevated CCL2 levels and CCR2^+^ monocyte infiltration are associated with GA (Fig 1). To evaluate whether the pharmacological inhibition of CCR2 is able to inhibit CX3CR1-dependent subretinal MP accumulation and photoreceptor degeneration, we treated light-challenged *Cx3cr1*^*−/−*^ mice systemically with a CCR2 inhibitor (RS 102895, Tocris). Control treated light-challenged *Cx3cr1*^*−/−*^ mice revealed numerous subretinal IBA-1^+^ MPs at day 14 (Fig 7A) compared to CCR2 inhibitor treated *Cx3cr1*^*−/−*^ mice (Fig 7B). Quantification of subretinal MP accumulation shows that systemic pharmacological inhibition of CCR2 prevented the accumulation (Fig 7C), similar to genetic deletion of *Ccl2*. Furthermore, the occurrence of photoreceptor apoptosis in control treated light-challenged *Cx3cr1*^*−/−*^ mice (Fig 7D) was significantly inhibited in the mice treated with the CCR2 inhibitor (Fig 7E and quantification in 7F). These results pharmacologically confirm that the inhibition of the CCL2/CCR2 axis attenuates the photoreceptor degeneration observed in *Cx3cr1*^*−/−*^ mice.

## DISCUSSION

Our results show that intraocular CCL2 levels are increased in GA and that an increase of CCL2 is found in the atrophic lesions. Elevated *Ccl2* mRNA and protein levels in atrophic and wet AMD (Jonas et al, 2010; Newman et al, 2012) support the possible involvement of CCL2 in the associated inflammation of both the atrophic and neovascular forms of AMD.

Immunohistochemistry using the pan-MP marker IBA-1 on human controls and early GA donor sections confirmed previous reports that the photoreceptor cell layer is exempt of MPs in healthy subjects, but that MPs accumulate in the photoreceptor cell layer and subretinal space in GA (Combadiere et al, 2007; Gupta et al, 2003). The density of inner retinal MPs was similar in GA lesions and macular sections from healthy donors. To better define the nature of MPs in GA we performed CCR2 immunohistochemistry to distinguish iMos from resident Mϕs and MCs. The CCR2 specific antibody we used selectively recognized monocytes in a human blood smear and intraluminal cells of monocytic morphology in human paraffin donor eye sections (Fig 1). Other than intraluminal cells, none of the healthy control sections revealed CCR2^+^ cells in the retina. In contrast, all the studied GA lesions reproducibly contained CCR2^+^ cells in a similar density and CCR2^+^ cells were observed in laminar deposits and soft drusen of sections of patients with early AMD. All the observed CCR2^+^ cells were also positive for CD18^+^ and displayed morphological features of Mos/Mϕs in terms of nuclear shape and cytoplasm/nuclear ratio rather than natural killer T lymphocytes, most likely identifying them as CCR2^+^ inflammatory monocytes derived MPs. In summary, we provide the first evidence that CCR2^+^ inflammatory monocytes infiltrate the subretinal space in the early atrophic lesions of GA.

Sustained presence of CCR2^+^ monocyte-derived inflammatory MPs have been shown to play an important role in neurodegenerative diseases such as models of multiple sclerosis, experimental autoimmune encephalitis and stroke (Conductier et al, 2010; Ransohoff, 2009). Recent evidence shows that CCR2^+^ monocyte recruitment also plays an important role in photoreceptor degeneration in models of retinitis pigmentosa (Guo et al, 2012), photo-oxidative stress (Rutar et al, 2012; Suzuki et al, 2012), and in the carboxyethylpyrrole-immunization induced model of AMD (Cruz-Guilloty et al, 2013). In GA, the prolonged presence of CCR2^+^ monocytes could participate in photoreceptor degeneration, maintain subretinal inflammation, and promote the expansion of the lesion. Previous reports of subretinal MP accumulation in AMD (Combadiere et al, 2007; Gupta et al, 2003) using nonspecific markers could not distinguish between potentially neurotoxic CCR2^+^ monocyte-derived inflammatory MPs (Ransohoff, 2009) and resident MCs.

*Cx3cr1* polymorphisms have been associated with wet AMD in some studies (Anastasopoulos et al, 2012, 2012 #1778; Combadiere et al, 2007; Tuo et al, 2004; Yang et al, 2010), but their possible involvement in GA is unknown. Unlike CCL2, CX3CL1 is constitutively expressed as a transmembrane protein in retinal neurons (Silverman et al, 2003) and is known to provide a tonic inhibitory signal to CX3CR1 bearing MCs that keeps these cells in a quiescent surveillance mode in the brain (Ransohoff, 2009). CX3CL1 levels in the aqueous humour of controls were very low; very little cleaved CX3CL1 seems to reach the aqueous humour under physiological conditions and no difference was observed in the GA group. However, the loss of CX3CL1/CX3CR1 signalling in the retina, leads to a strong age-dependent increase of subretinal MP accumulation, as observed in *Cx3cr1*-deficient mice compared to wildtype mice. Importantly, the accumulation of subretinal *Cx3cr1*-deficient MPs is associated with photoreceptor degeneration (Combadiere et al, 2007; Ma et al, 2009; Raoul et al, 2008a). Although aged *Cx3cr1*-deficient mice do not mimic all aspects of AMD (drusen formation and RPE atrophy) they do model chronic subretinal MP accumulation and associated photoreceptor degeneration, which are hallmarks of AMD (Combadiere et al, 2007; Gupta et al, 2003). Compared to other models in which subretinal inflammation occurs secondarily to light-injury or genetic defects (Cruz-Guilloty et al, 2013; Guo et al, 2012; Kohno et al, 2013; Rutar et al, 2012; Suzuki et al, 2012), the *Cx3cr1* knockout mouse model presents a primary MP accumulation in the absence of an inherited retinal degeneration (C57BL/6J background) (Combadiere et al, 2007). The absence of confounding factors that lead to photoreceptor cell death in the *Cx3cr1* knockout mouse model makes it a particularly suitable model for the study of subretinal inflammation and its influence on photoreceptor homeostasis. Exaggerated subretinal MP accumulation and photoreceptor degeneration can also be induced acutely by a light-challenge in *Cx3cr1* knockout mice (Raoul et al, 2008a). The intensity of the light-challenge model used herein was developed to induce subretinal inflammation and subsequent photoreceptor degeneration in the *Cx3cr1*^*−/−*^ mice but not in *Cx3cr1*^*+/+*^ mice (see below). The light intensity we used in these experiments is not strong enough to directly induce photoreceptor apoptosis in pigmented wildtype animals, contrary to classically used light-injury models.

Similar to the human disease, we show that retinal CCL2 production is increased in aged and light-challenged *Cx3cr1* deficient mice (Fig 2). Several lines of evidence suggest that subretinal *Cx3cr1* deficient MPs are the main source of CCL2 release in the subretinal inflammation observed in *Cx3cr1* knockout mice: CCL2 localized to subretinal *Cx3cr1* deficient MPs in immunohistochemistry, *Ccl2* induction observed in light-challenged *Cx3cr1*^*GFP/GFP*^ mice is suppressed in *Cx3cr1*^*GFP/GFP*^*Ccr2*^*RFP/RFP*^ mice (that are protected against subretinal MP accumulation), *Ccl2* mRNA is strongly enriched in cell-sorted inflammatory monocytes in the eyes of light-challenged *Cx3cr1*^*GFP/GFP*^ mice and *Ccl2* induction is increased in *Cx3cr1*^*−/−*^ monocyte-derived Mφ *in vitro* differentiated in the presence of POS. Activated Mφs have been described releasing CCL2 in neuro-inflammatory conditions such as multiple sclerosis *in vivo* (Simpson et al, 1998). CX3CR1 is strongly and constitutively expressed by MCs and upregulated in monocyte-derived Mϕs (Gautier et al, 2012; Geissmann et al, 2010). CX3CR1/CX3CL1 signalling physiologically represses the expression of inflammatory mediators in CNS pathology and protects neurons in inflammatory conditions (Cardona et al, 2006; Ransohoff, 2009). We show that the absence of CX3CR1/CX3CL1 signalling in the subretinal environment fails to repress CCL2 induction and that CCL2 is overexpressed in subretinal inflammation in *Cx3cr1* deficient mice (aged and light-challenged) and in monocyte-derived macrophages in the presence of POS *in vitro*. The accumulation of CCL2 overexpressing *Cx3cr1*^*−/−*^ Mos might thereby lead to a positive feedback in aged- and light-challenged *Cx3cr1*^*−/−*^ mice, after an initial recruitment through other chemotactic agents.

Others and we have shown that *Cx3cr1* deficiency leads to accumulation of MPs in the central nervous system in pathological conditions (Cardona et al, 2006; Chinnery et al, 2011; Combadiere et al, 2007; Kezic et al, 2013). It is unclear to what extent the recruitment of retinal MCs or blood born CCR2+ monocytes participates in subretinal MP accumulation. Our experiments of *Cx3cr1* and *Ccr2* mRNA analysis on monocytes and purified retinal MCs, and GFP and RFP localization in *Cx3cr1*^*+/GFP*^*Ccr2*^*+/RFP*^ confirm that CX3CR1, but not CCR2, is strongly expressed in retinal MCs (Mizutani et al, 2011) (Fig 5). As previously described in the brain, the expression of CCR2 can thereby differentiate freshly recruited blood monocyte-derived macrophages in the subretinal space (Saederup et al, 2010). Submitting *Cx3cr1*^*GFP/GFP*^*Ccr2*^*+/RFP*^ mice to the light-induced subretinal accumulation of MPs, we show that CCR2 expressing RFP^+^ cells participate in subretinal inflammation (Fig 5). These results suggest that CCR2^+^ monocytes are recruited to the subretinal space from the blood in light-challenged *Cx3cr1*^*GFP/GFP*^ mice. *Ccr2* mRNA has been shown to be downregulated in monocyte to macrophage differentiation (Wong et al, 1997) and we demonstrate its downregulation in wildtype and *Cx3cr1*^*−/−*^ monocyte differentiated macrophages in contact with POS after 18 hours. To better evaluate the extent of CCR2^+^ Mos in subretinal MP accumulation, we permanently marked the circulating monocytes with repeated EdU injections prior to and during light-induced subretinal recruitment in *Cx3cr1*^*−/−*^ mice and compared the numbers of subretinal EdU^+^ MPs in mice with and without clodronate-liposome-induced circulating monocyte depletion. Local EdU administration failed to mark subretinal MPs, suggesting that ocular MP proliferation does not play a significant role in the light-induced accumulation. The fact that 37% of subretinal MPs were EdU positive in mice receiving systemic EdU injections and control liposomes, seems to suggest that at least one third of subretinal MPs originated from the circulation (Fig 5). Considering that the EdU injections only marked 76% of circulating monocytes, the extent of Mo participation in light-induced subretinal MP accumulation using this method is possibly underestimated. Indeed, monocyte depletion inhibited the subretinal IBA-1^+^ MP accumulation by 50–60% (Fig 5), suggesting that as many as half of the subretinal MP population might originate from blood-borne Mos and half from local resident Mϕs or MCs. Similarly, genetic *Ccl2* or *Ccr2* deletion and CCR2 inhibitors diminished subretinal MP accumulation by 50–60% in *Cx3cr1*^*−/−*^and *Cx3cr1*^*GFP/GFP*^ mice with age- and in the light-challenge-induced model (Figs 2, 3 and 7). Interestingly, although light-induced subretinal MP accumulation represents an acute inflammation and might therefore be quite different from the chronic inflammation observed with age, the inhibition of CCL2/CCR2 prevented subretinal inflammation in the same order of magnitude.

Interestingly, the inhibition of CCR2^+^ monocyte recruitment (*Ccl2* or *Ccr2* deletion, monocyte depletion and pharmacological inhibition Figs 3, 4, 5 and 7) nearly completely inhibited photoreceptor degeneration in *Cx3cr1*^*−/−*^ mice. These results could suggest that (i) the CCR2^+^ monocyte-derived MPs are the main mediators of neurotoxicity and (ii) that the subretinal accumulation of MPs is in part due to CCR2^neg^ cells, possibly retinal MCs that display no or little toxicity. Indeed, our *in vitro* experiments comparing wildtype and *Cx3cr1*^*−/−*^ Mo and MC toxicity on photoreceptors of retinal explants in a co-culture system confirm increased toxicity of Mos compared to MCs and of *Cx3cr1*^*−/−*^ MPs compared to wildtype MPs (Fig 6). The neuroprotective effect of inhibiting monocyte recruitment (via CCL2/CCR2 deletion or circulating monocyte depletion) in models of photoreceptor degeneration such as photo-oxidative stress (Rutar et al, 2012; Suzuki et al, 2012), the *Abca4*^*−/−*^*Rdh8*^*−/−*^ mouse Stargardt/AMD model (Kohno et al, 2013), and in a carboxyethylpyrrole immunization induced AMD model (Cruz-Guilloty et al, 2013), seem to confirm this observation, while the role of monocyte recruitemnt in *rd1* mice remains a point of controversy (Guo et al, 2012; Sasahara et al, 2008).

Aged *Ccl2*^*−/−*^ and *Ccr2*^*−/−*^ mice have also been described to develop the more discrete subretinal MP accumulation that occurs in older mice compared to *Cx3cr1* deficient mice, associated with little to no photoreceptor degeneration (see Supporting Information data) (Chen et al, 2011; Luhmann et al, 2009). It is not clear how *Ccl2* and *Ccr2* deficiency lead to subretinal MP accumulation, but subretinal IBA-1^+^cells in aged *Ccl2*^*−/−*^ and *Ccr2*^*−/−*^ mice are likely not derived from CCR2^+^ monocytes but may at least in part be CCR2^-^CX3CR1^high^ MCs and/or the non-inflammatory CX3CR1^+^ monocytes, which could explain the absence of photoreceptor degeneration.

In summary, contrary to CCR2 and CCL2, CX3CR1 and CX3CL1 are constitutively expressed in the retina similar to the brain (Ransohoff & Cardona, 2010). CX3CR1 signalling suppresses the expression of inflammatory mediators, such as CCL2 in CCR2^+^ monocyte derived inflammatory MPs in the subretinal microenvironment. Contrary to wildtype mice, age- and light-induced stress in *Cx3cr1* deficient animals are sufficient to induce CCL2 expression and CCR2^+^ monocyte recruitment to the subretinal space at a rate exceeding that of their clearance, and subretinal MPs accumulate. In the brain, *Cx3cr1*^*−/−*^ Mϕs clear less efficiently from the injection site compared to wildtype macrophages (Cardona et al, 2006). We reported that *Cx3cr1*^*−/−*^ Mϕs present a similar impaired clearance from the subretinal space compared to wildtype Mϕs (Levy et al, 2011) and are currently investigating the molecular mechanism behind this phenomenon. The combination of increased Mo recruitment and decreased MP clearance possibly explains the pathological MP accumulation observed in *Cx3cr1*^*−/−*^ mice. This pathological accumulation, in combination with the increased neurotoxicity of *Cx3cr1*^*−/−*^ MPs (Cardona et al, 2006) and in particular *Cx3cr1*^*−/−*^ Mos (Fig 6) might explain the subretinal inflammation and photoreceptor degeneration observed in *Cx3cr1*^*−/−*^ mice.

Chronic CCR2^+^ inflammatory monocyte recruitment has recently been shown to mediate photoreceptor degeneration in other models of retinal degeneration and AMD (Cruz-Guilloty et al, 2013; Guo et al, 2012; Kohno et al, 2013; Rutar et al, 2012; Suzuki et al, 2012). Even though *Cx3cr1*^*−/−*^ mice and other animal models of photoreceptor degeneration only incompletely model GA, the observation that, in most models, CCR2^+^ inflammatory monocytes are toxic to photoreceptors might have important implications for AMD. Atrophic AMD is associated with elevated CCL2 levels and CCR2^+^ inflammatory monocyte infiltration into the lesion. The initial lesion formation might be due to local factors and be independent of inflammatory monocyte infiltration, but their presence might subsequently be a driving factor in the lesion expansion that leads to central vision loss. Initial lesions often develop parafoveally (Sarks et al, 1988) and inhibiting their expansion could be a precious tool in preserving central vision. There are currently no efficient therapies to inhibit lesion growth and the central vision destruction in GA. We propose that the continued recruitment of CCR2^+^ inflammatory monocytes and the release of neurotoxic factors contribute to the degeneration of the adjacent retina in atrophic AMD. CCL2/CCR2 blocking agents hold the potential to inhibit devastating chronic inflammation under the retina in wet and atrophic AMD.

## MATERIALS AND METHODS

### Animals

*Cx3cr1*^*−/−*^*/Ccl2*^*−/−*^ mouse strains on C57BL/6 background were generated from *Cx3cr1*^*−/−*^ mice (Combadiere et al, 2003) and *Ccl2*^*−/−*^ mice (from B. Rollins, Department of Medical Oncology, Dana-Farber Cancer Institute, Boston, MA, USA). *Cx3cr1*^*GFP/GFP*^*Ccr2*^*RFP/RFP*^ mice were generated as previously described (Saederup et al, 2010). All mice used in this study were negative for the *rd8* mutation. Mice were maintained at the animal facility under pathogen-free conditions. All animals were housed in a 12/12 h light/dark (100–500 lx) cycle with food and water available *ad libitum*. Animal experiments were approved by the Institutional Animal Care and Use Committee of the Faculté de Médecine Pitié-Salpétrière.

### CCL2 and CCR2 immunohistochemistry

Donor eyes with a known history of AMD and controls were collected through the Minnesota Lions Eye bank (5 control maculae from 5 patients; 4 early AMD donor maculae from 3 individuals; 10 GA donor maculae from 7 patients). Postmortem fundus photographs were taken and the posterior segment was fixed 4 h in 4%PFA, dissected, imbedded in paraffin, and sectioned. Control blood smear was fixed 4 h in 4%PFA and treated as the paraffin sections after rehydration. CCL2 (clone 5d3f7, mouse-anti-human, formic acid antigen retrieval, Alexis Biochemicals), CCR2 (AB32144, rabbit-anti-human, citrate buffer heat antigen retrieval, Abcam), IBA-1 (rabbit-anti-human, formic acid antigen retrieval, Wako Chemicals), and CD18 (MCA503, rat-anti-human, citrate buffer heat antigen retrieval, Abd Serotec) immunohistochemistal analyses were performed and revealed using appropriate fluorescent or horseradish peroxidase coupled secondary antibodies (Molecular Probe) using a peroxidase substrate kit (Vector).

### Blood monocyte and microglial cell preparation

Retinas were collected from PBS perfused 11-week-old mice. Tissues were dissociated by papain in HBSS for 30 min at 37°C under agitation (Neural Tissue Dissociation kit, Miltenyi Biotech). Myeloid cells were isolated using Percoll gradient (Percoll Plus, GE Healthcare). Cell suspension was blocked 15 min on ice in PBS EDTA 2 mM 3% rat serum with anti-CD16/CD32 (SeroBlock, Abd Serotec). Resident MCs were purified by flow cytometry on fluorescence activated cell sorter (BDFACSVantage SE, BD Biosciences) as CD11b^high^ (PE) and CD45^low^ (PE-Cy7) (Abd Serotec) and were recovered directly in lysis buffer. Enriched monocytes were obtained by centrifugation of whole blood on a Ficoll Paque layer (GE Healthcare). Positive selection by adhesion properties on culture dishes was performed 90 min to obtain monocytes. Total mRNA was extracted with Nucleospin RNA XS kit (Macherey Nagel).

### Monocyte culture and photoreceptor outer segment (POS) incubation

POS were prepared from porcine retina as previously described (Molday et al, 1987). C57BL/6 or *Cx3cr1*^*−/−*^ monocytes were sorted with EasySep Mouse Monocyte Enrichment kit (StemCells Technology), seeded in 96 well plates (500,000 well^−1^) and POS (2 × 106) were added in 50 µl of DMEM containing 2.5% sucrose. After 18 h total RNA was isolated from the monocytes.

### Monocytes, microglial cells and retinal explant cocultures

C57BL/6 or *Cx3cr1*^*−/−*^ monocytes from bone marrow were sorted with EasySep Mouse Monocyte Enrichment kit (StemCells Technology). C57BL/6 or *Cx3cr1*^*−/−*^ MCs were prepared from PBS perfused mice. After dissociation of the brain with the Neural Tissue Dissociation Kit and Percoll gradient centrifugation, cells were labelled with anti-CD11b microbeads antibody (Miltenyi Biotech). MCs were purified by selection on MS columns in a magnetic field (OctoMACS magnet, Miltenyi). Monocytes and MCs were then seeded on polycarbonate filters floating on DMEM. C57BL/6 retina were prepared and placed with the photoreceptors facing 100,000 adherent monocytes or MCs while control monocytes and MCs were cultivated without the overlying explant. After 18 h, the explants were carefully removed and TUNEL labelling was performed on the retinal explants as described for retina below.

### ELISA

Human CCL2 and CX3CL1 concentrations were measured in aqueous humours sampled during cataract surgery from 18 patients suffering from geographic AMD and 22 age-matched control donors. All patients were treated at the same institution. An inclusion criterion in the study group was the absence of any retinal or optic nerve disease except GA. The Medical Ethics Committee of the Hôtel Dieu Hospital approved the study protocol. Samples were normalized for protein content and CCL2 was quantified with Quantikine ELISA assay as described by the manufacturer (R&D Systems). Optical Density was determined at 450 nm.

For mice, retina/RPE/choroid complex was resuspended in 200 µl PBS with protease inhibitor cocktail (Calbiochem), homogenized and centrifuged at 14,000*g* for 10 min at 4°C and protein concentration measured using Bradford prior to ELISA.

**The paper explained**

PROBLEM:

Age-related macular degeneration (AMD) is the leading cause of irreversible blindness in the industrialized world. There are two clinical forms of late AMD: the fast developing exudative form defined by choroidal neovascularization (CNV) and the slow-developing atrophic form characterized by an extending lesion of the photoreceptors known as geographic atrophy. While considerable progress has been made in the treatment of wet AMD, there is currently no efficient treatment to halt the slow expansion of geographic atrophy and its pathophysiology is unknown. Inflammatory monocytes strongly express the chemokine receptor CCR2 and are attracted to sites of inflammation that express CCL2. In the central nervous system, excessive monocyte recruitment can lead to collateral damage and participate in neurodegeneration. The role of inflammatory monocytes in geographic atrophy is unknown.

RESULTS:

This is the first study to show that CCL2 levels are elevated in eyes of patients with geographic atrophy and that CCR2^+^ inflammatory monocytes are recruited to the atrophic lesion. CCL2 levels and CCR2^+^ monocytes were also higher in eyes of *Cx3cr1* knockout mice that develop subretinal inflammation and photoreceptor degeneration. Ccl2 and Ccr2 deletion, pharmacological CCR2 inhibition and monocyte depletion greatly diminished subretinal inflammation and photoreceptor degeneration in *Cx3cr1* knockout mice.

IMPACT:

We propose that the continued recruitment of CCR2^+^ inflammatory monocytes and their release of neurotoxic factors contribute to the retinal degeneration and lesion expansion observed in geographic atrophy. CCL2/CCR2 blocking agents hold the potential to inhibit devastating chronic inflammation under the retina in geographic atrophy.

### Reverse transcription and real-time polymerase chain reaction (RT-PCR)

Total RNA was isolated with Nucleospin RNAII (Macherey Nagel). Single-stranded cDNA was synthesized from total RNA (pretreated with DNaseI amplification grade) using oligo-dT as primer and superscript II reverse transcriptase (Life technologies). Subsequent RT-PCR was performed using cDNA, Taqman Gene Expression Master Mix (Life technologies) and primers (0.5 pmol/µl) available upon request. Results were normalized by expression of β-actin or S26. PCR reactions were performed in 45 cycles of 15 s at 95°C, 45 s at 60°C.

### Light-challenge model

Two- to four-month-old mice were adapted to darkness for 6 h and pupils were fully dilated with 1% Atropin (Novartis). Animals were then exposed to green LED light (4500 Lux, JP Vezon équipements) for 4 days and subsequently kept in cyclic 12 h/12 h normal animal facility conditions. MP accumulation and retinal degeneration were assessed respectively at 10 and 17 days after light exposure. For some experiments, mice were treated daily during green-light exposition with daily intravenous injections of the CCR2 inhibitor RS 102895 (45 µg/mouse, Tocris, Biosciences), 200 µl clodronate-liposomes or liposomes (provided by Dr. N. Van Rooijen) in PBS.

### *In vivo* EdU experiments

Mice were treated daily by three intraperitoneal injections of EdU (25 mg/kg, Life Technologies) and once daily with intravenous empty liposomes (control) or chlodronate liposomes (30 mg/kg, FormuMax). Blood smears were performed and eyes were collected. Choroid/RPE and retinal flatmounts were labelled with IBA-1 and revealed for EdU incorporation (Click-iT EdU imaging kit, Life Technologies Corporation) for the quantification of subretinal IBA-1^+^ and EdU^+^ cells. Blood smears, were labelled with anti-MCSF-R antibody (Biolegend) and EdU to calculate the proportion of EdU^+^ monocytes in the circulation.

### Cell sorting

*Cx3cr1*^*GFP/GFP*^ mice were exposed to green light for 4 days. Mice were perfused with cold PBS. Eyes were dissected and tissues were dissociated by Neural Tissue Dissociation Kit 30 min and filtered in HBSS 10% bovine serum with a 70 µm sieve. Cell suspensions were blocked with PBS EDTA 2 mM, 3% rat serum, 2% anti-CD16/CD32 15 min on ice. The tissues were labelled 25 min on ice with anti-CD11b-PerCPCy5.5, Ly6C-PE and Ly6G-APC (BD Biosciences). 10% of the whole cell suspension was directly lysed. The cells were sorted using a *MoFlo Astrios* (Beckman Coulter) and cells were directly sorted in lysis buffer to obtain mRNA from CD11b^pos^ Ly6G^neg^ Ly6C^neg^ GFP^high^ resident cells, and CD11b^pos^ Ly6G^neg^ Ly6C^pos^ GFP^low^ inflammatory Mo/Mϕs.

### Choroidal flatmounts and MP quantification

Eyes were enucleated, fixed in 4% PFA and sectioned at the limbus; the cornea and lens were discarded. The retinas were carefully peeled from the RPE/choroid/sclera. Retinas were fixed for additional 20 min in cold acetone. Retinas and choroids were incubated with anti-IBA-1 (Wako Chemicals) followed by secondary antibody anti-rabbit Alexa 488 (Molecular probes). Choroids and retinas were flatmounted and viewed with a fluorescence microscope DM5500B (Leica). MPs were counted on whole RPE/choroidal flatmounts and on the outer segment side of the retina.

### Histology

Eyes were fixed in 0.5% glutaraldehyde, 4% PFA for 2 h, dehydrated and mounted in Historesin (Leica). 5 µm oriented sections crossing inferior pole, optic nerve and superior pole were cut and stained with toluidin blue. Rows of nuclei in the ONL were counted at different distances from the optic nerve.

### Terminal deoxynucleotidyl transferase dUTP nick end labelling (TUNEL)

4% PFA fixed retinal flatmounts were pre-treated with frozen methanol for 30 min and then frozen methanol/acetic acid (2:1) for another 30 min. After washing with PBS, flatmounts were incubated overnight at 4°C with the reaction mixture as described by manufacturer's protocol (In Situ Cell Death Detection Kit, Roche Diagnostics) and then for 90 min at 37°C. After reaction was stopped by washing with PBS at RT, nuclei were counterstained with Hoechst (Sigma–Aldrich).

### Fundus photography

Mice were anaesthetized by i.p. injection of ketamine (50 mg/kg) and xylazine (10 mg/kg). Pupils were fully dilated with 0.5% tropicamide and 10% neosynephrine. Coverslips positioned on the mouse cornea were used as a contact glass. Fundus photographs were taken with a digital CCD camera (Nikon D3) coupled with an endoscope (Karl Storz) as previously described (Paques et al, 2006).

### Statistics

Graph Pad Prism 5 (GraphPad Software) was used for data analysis and graphic representation. All values are reported as mean ± SEM. Statistical analysis was performed by one-way or two-way Anova analysis of variance followed by Bonferroni post-test, students *t*-tes or Mann–Whitney test for comparison among means depending on the experimental design. The *p*-values are indicated in the figure legends.

## Supplementary material

As a service to our authors and readers, this journal provides supporting information supplied by the authors. Such materials are peer reviewed and may be re-organized for online delivery, but are not copy-edited or typeset. Technical support issues arising from supporting information (other than missing files) should be addressed to the authors.

**Review Process File**

**Table S1.** Demographic data of the study group of patients with geographic atrophy and the control group of patients without retinal disorders and undergoing routine cataract surgery.

**Figure S1.** CCR2 immunohistochemistry on healthy human blood smear.

**Figure S2.** Negative control and examples of CCR2^+^ immunohistochemistry on sections with geographic atrophy and age related maculopathy.

**Figure S3.** Intravitreal EdU injections in light-challenged Cx3cr1^−/−^ mice.

**Figure S4.** Comparaison of subretinal MP accumulation in C57BL/6, Ccl2^−/−^ and Cx3cr1^−/−^ mice at 18 months under the same animal housing conditions.

**Figure S5.** Photoreceptor status in 3-month-old C57BL/6 and Cx3cr1^−/−^ mice.

## References

[b1] Ambati J, Anand A, Fernandez S, Sakurai E, Lynn BC, Kuziel WA, Rollins BJ, Ambati BK (2003). An animal model of age-related macular degeneration in senescent Ccl-2- or Ccr-2-deficient mice. Nat Med.

[b2] Anastasopoulos E, Kakoulidou A, Coleman AL, Sinsheimer JS, Wilson MR, Yu F, Salonikiou A, Koskosas A, Pappas T, Founti P (2012). Association of sequence variation in the CX3CR1 gene with geographic atrophy age-related macular degeneration in a Greek population. Curr Eye Res.

[b3] Arnold L, Henry A, Poron F, Baba-Amer Y, van Rooijen N, Plonquet A, Gherardi RK, Chazaud B (2007). Inflammatory monocytes recruited after skeletal muscle injury switch into antiinflammatory macrophages to support myogenesis. J Exp Med.

[b4] Bazan JF, Bacon KB, Hardiman G, Wang W, Soo K, Rossi D, Greaves DR, Zlotnik A, Schall TJ (1997). A new class of membrane-bound chemokine with a CX3C motif. Nature.

[b5] Cardona AE, Pioro EP, Sasse ME, Kostenko V, Cardona SM, Dijkstra IM, Huang D, Kidd G, Dombrowski S, Dutta R (2006). Control of microglial neurotoxicity by the fractalkine receptor. Nat Neurosci.

[b6] Chen M, Forrester JV, Xu H (2011). Dysregulation in retinal para-inflammation and age-related retinal degeneration in CCL2 or CCR2 deficient mice. PLoS One.

[b7] Chen M, Luo C, Penalva R, Xu H (2013). Paraquat-induced retinal degeneration is exaggerated in CX3CR1-deficient mice and is associated with increased retinal inflammation. Invest Ophthalmol Vis Sci.

[b8] Chen M, Zhao J, Luo C, Pandi SP, Penalva RG, Fitzgerald DC, Xu H (2012). Para-inflammation-mediated retinal recruitment of bone marrow-derived myeloid cells following whole-body irradiation is CCL2 dependent. Glia.

[b9] Chinnery HR, McLenachan S, Humphries T, Kezic JM, Chen X, Ruitenberg MJ, McMenamin PG (2011). Accumulation of murine subretinal macrophages: Effects of age, pigmentation and CX(3)CR1. Neurobiol Aging.

[b10] Chow A, Brown BD, Merad M (2011). Studying the mononuclear phagocyte system in the molecular age. Nat Rev Immunol.

[b11] Combadiere C, Feumi C, Raoul W, Keller N, Rodero M, Pezard A, Lavalette S, Houssier M, Jonet L (2007). CX3CR1-dependent subretinal microglia cell accumulation is associated with cardinal features of age-related macular degeneration. J Clin Invest.

[b12] Combadiere C, Potteaux S, Gao JL, Esposito B, Casanova S, Lee EJ, Debre P, Tedgui A, Murphy PM, Mallat Z (2003). Decreased atherosclerotic lesion formation in CX3CR1/apolipoprotein E double knockout mice. Circulation.

[b13] Combadiere C, Potteaux S, Rodero M, Simon T, Pezard A, Esposito B, Merval R, Proudfoot A, Tedgui A, Mallat Z (2008). Combined inhibition of CCL2, CX3CR1, and CCR5 abrogates Ly6C(hi) and Ly6C(lo) monocytosis and almost abolishes atherosclerosis in hypercholesterolemic mice. Circulation.

[b14] Conductier G, Blondeau N, Guyon A, Nahon JL, Rovere C (2010). The role of monocyte chemoattractant protein MCP1/CCL2 in neuroinflammatory diseases. J Neuroimmunol.

[b15] Cruz-Guilloty F, Saeed AM, Echegaray JJ, Duffort S, Ballmick A, Tan Y, Betancourt M, Viteri E, Ramkhellawan GC, Ewald E (2013). Infiltration of proinflammatory m1 macrophages into the outer retina precedes damage in a mouse model of age-related macular degeneration. Int J Inflam.

[b16] Edwards AO, Ritter R, Abel KJ, Manning A, Panhuysen C, Farrer LA (2005). Complement factor H polymorphism and age-related macular degeneration. Science.

[b17] Fife BT, Huffnagle GB, Kuziel WA, Karpus WJ (2000). CC chemokine receptor 2 is critical for induction of experimental autoimmune encephalomyelitis. J Exp Med.

[b18] Gautier EL, Shay T, Miller J, Greter M, Jakubzick C, Ivanov S, Helft J, Chow A, Elpek KG, Gordonov S (2012). Gene-expression profiles and transcriptional regulatory pathways that underlie the identity and diversity of mouse tissue macrophages. Nat Immunol.

[b19] Geissmann F, Jung S, Littman DR (2003). Blood monocytes consist of two principal subsets with distinct migratory properties. Immunity.

[b20] Geissmann F, Manz MG, Jung S, Sieweke MH, Merad M, Ley K (2010). Development of monocytes, macrophages, and dendritic cells. Science.

[b21] Guo C, Otani A, Oishi A, Kojima H, Makiyama Y, Nakagawa S, Yoshimura N (2012). Knockout of ccr2 alleviates photoreceptor cell death in a model of retinitis pigmentosa. Exp Eye Res.

[b22] Gupta N, Brown KE, Milam AH (2003). Activated microglia in human retinitis pigmentosa, late-onset retinal degeneration, and age-related macular degeneration. Exp Eye Res.

[b23] Guymer RH, Tao LW, Goh JK, Liew D, Ischenko O, Robman LD, Aung K, Cipriani T, Cain M, Richardson AJ (2011). Identification of Urinary Biomarkers for Age-related Macular Degeneration. Invest Ophthalmol Vis Sci.

[b24] Haines JL, Hauser MA, Schmidt S, Scott WK, Olson LM, Gallins P, Spencer KL, Kwan SY, Noureddine M, Gilbert JR (2005). Complement factor H variant increases the risk of age-related macular degeneration. Science.

[b25] Hisatomi T, Sakamoto T, Sonoda KH, Tsutsumi C, Qiao H, Enaida H, Yamanaka I, Kubota T, Ishibashi T, Kura S (2003). Clearance of apoptotic photoreceptors: Elimination of apoptotic debris into the subretinal space and macrophage-mediated phagocytosis via phosphatidylserine receptor and integrin alphavbeta3. Am J Pathol.

[b26] Hong T, Tan AG, Mitchell P, Wang JJ (2011). A Review and meta-analysis of the association between c-reactive protein and age-related macular degeneration. Surv Ophthalmol.

[b27] Huang DR, Wang J, Kivisakk P, Rollins BJ, Ransohoff RM (2001). Absence of monocyte chemoattractant protein 1 in mice leads to decreased local macrophage recruitment and antigen-specific T helper cell type 1 immune response in experimental autoimmune encephalomyelitis. J Exp Med.

[b28] Huynh ML, Fadok VA, Henson PM (2002). Phosphatidylserine-dependent ingestion of apoptotic cells promotes TGF-beta1 secretion and the resolution of inflammation. J Clin Invest.

[b29] Izikson L, Klein RS, Charo IF, Weiner HL, Luster AD (2000). Resistance to experimental autoimmune encephalomyelitis in mice lacking the CC chemokine receptor (CCR)2. J Exp Med.

[b30] Jonas JB, Tao Y, Neumaier M, Findeisen P (2010). Monocyte chemoattractant protein 1, intercellular adhesion molecule 1, and vascular cell adhesion molecule 1 in exudative age-related macular degeneration. Arch Ophthalmol.

[b31] Kezic JM, Chen X, Rakoczy EP, McMenamin PG (2013). The effects of age and Cx3cr1 deficiency on retinal microglia in the Ins2Akita diabetic mouse. Invest Ophthalmol Vis Sci.

[b32] Klein R, Klein BE, Knudtson MD, Meuer SM, Swift M, Gangnon RE (2007). Fifteen-year cumulative incidence of age-related macular degeneration: the Beaver Dam Eye Study. Ophthalmology.

[b33] Klein R, Peto T, Bird A, Vannewkirk MR (2004). The epidemiology of age-related macular degeneration. Am J Ophthalmol.

[b34] Klein RJ, Zeiss C, Chew EY, Tsai JY, Sackler RS, Haynes C, Henning AK, SanGiovanni JP, Mane SM, Mayne ST (2005). Complement factor H polymorphism in age-related macular degeneration. Science.

[b35] Kohno H, Chen Y, Kevany BM, Pearlman E, Miyagi M, Maeda T, Palczewski K, Maeda A (2013). Photoreceptor proteins initiate microglial activation via toll-like receptor 4 in retinal degeneration mediated by all-trans-retinal. J Biol Chem.

[b36] Levy OE, Calippe BE, Raoul W, Camelo S, Lavalette S, Guillonneau X, Combadiere C, Sennlaub F (2011). CX3CR1 deficient macrophages present an impaired clearance from the subretinal space. ARVO Meeting Abstr.

[b37] Luhmann UF, Lange CA, Robbie S, Munro PM, Cowing JA, Armer HE, Luong V, Carvalho LS, MacLaren RE, Fitzke FW (2012). Differential modulation of retinal degeneration by Ccl2 and Cx3cr1 chemokine signalling. PLoS One.

[b38] Luhmann UF, Robbie S, Munro PM, Barker SE, Duran Y, Luong V, Fitzke FW, Bainbridge J, Ali RR, Maclaren R (2009). The drusen-like phenotype in aging Ccl2 knockout mice is caused by an accelerated accumulation of swollen autofluorescent subretinal macrophages. Invest Ophthalmol Vis Sci.

[b39] Ma W, Zhao L, Fontainhas AM, Fariss RN, Wong WT (2009). Microglia in the mouse retina alter the structure and function of retinal pigmented epithelial cells: a potential cellular interaction relevant to AMD. PLoS One.

[b40] Machalinska A, Dziedziejko V, Mozolewska-Piotrowska K, Karczewicz D, Wiszniewska B, Machalinski B (2009). Elevated plasma levels of C3a complement compound in the exudative form of age-related macular degeneration. Ophthalmic Res.

[b41] Maller J, George S, Purcell S, Fagerness J, Altshuler D, Daly MJ, Seddon JM (2006). Common variation in three genes, including a noncoding variant in CFH, strongly influences risk of age-related macular degeneration. Nat Genet.

[b42] Mantovani A, Sica A, Sozzani S, Allavena P, Vecchi A, Locati M (2004). The chemokine system in diverse forms of macrophage activation and polarization. Trends Immunol.

[b43] Mattapallil MJ, Wawrousek EF, Chan CC, Zhao H, Roychoudhury J, Ferguson TA, Caspi RR (2012). The rd8 mutation of the Crb1 gene is present in vendor lines of C57BL/6N mice and embryonic stem cells, and confounds ocular induced mutant phenotypes. Invest Ophthalmol Vis Sci.

[b44] Miljanich GP, Nemes PP, White DL, Dratz EA (1981). The asymmetric transmembrane distribution of phosphatidylethanolamine, phosphatidylserine, and fatty acids of the bovine retinal rod outer segment disk membrane. J Membr Biol.

[b45] Mizutani M, Pino PA, Saederup N, Charo IF, Ransohoff RM, Cardona AE (2011). The fractalkine receptor but not CCR2 is present on microglia from embryonic development throughout adulthood. J Immunol.

[b46] Molday RS, Hicks D, Molday L (1987). Peripherin. A rim-specific membrane protein of rod outer segment discs. Invest Ophthalmol Vis Sci.

[b47] Nakazawa T, Hisatomi T, Nakazawa C, Noda K, Maruyama K, She H, Matsubara A, Miyahara S, Nakao S, Yin Y (2007). Monocyte chemoattractant protein 1 mediates retinal detachment-induced photoreceptor apoptosis. Proc Natl Acad Sci USA.

[b48] Newman AM, Gallo NB, Hancox LS, Miller NJ, Radeke CM, Maloney MA, Cooper JB, Hageman GS, Anderson DH, Johnson LV (2012). Systems-level analysis of age-related macular degeneration reveals global biomarkers and phenotype-specific functional networks. Genome Med.

[b49] Paques M, Simonutti M, Roux MJ, Picaud S, Levavasseur E, Bellman C, Sahel JA (2006). High resolution fundus imaging by confocal scanning laser ophthalmoscopy in the mouse. Vision Res.

[b50] Penfold PL, Liew SC, Madigan MC, Provis JM (1997). Modulation of major histocompatibility complex class II expression in retinas with age-related macular degeneration. Invest Ophthalmol Vis Sci.

[b51] Ransohoff RM (2009). Chemokines and chemokine receptors: Standing at the crossroads of immunobiology and neurobiology. Immunity.

[b52] Ransohoff RM, Cardona AE (2010). The myeloid cells of the central nervous system parenchyma. Nature.

[b53] Raoul W, Feumi C, Keller N, Lavalette S, Houssier M, Behar-Cohen F, Combadiere C, Sennlaub F (2008a). Lipid-bloated subretinal microglial cells are at the origin of drusen appearance in CX3CR1-deficient mice. Ophthalmic Res.

[b54] Raoul W, Keller N, Rodero M, Behar-Cohen F, Sennlaub F, Combadiere C (2008b). Role of the chemokine receptor CX3CR1 in the mobilization of phagocytic retinal microglial cells. J Neuroimmunol.

[b55] Rutar M, Natoli R, Provis JM (2012). Small interfering RNA-mediated suppression of Ccl2 in Muller cells attenuates microglial recruitment and photoreceptor death following retinal degeneration. J Neuroinflammation.

[b56] Saederup N, Cardona AE, Croft K, Mizutani M, Cotleur AC, Tsou CL, Ransohoff RM, Charo IF (2010). Selective chemokine receptor usage by central nervous system myeloid cells in CCR2-red fluorescent protein knock-in mice. PLoS One.

[b57] Sarks JP, Sarks SH, Killingsworth MC (1988). Evolution of geographic atrophy of the retinal pigment epithelium. Eye (Lond).

[b58] Sarks SH (1976). Ageing and degeneration in the macular region: A clinico-pathological study. Br J Ophthalmol.

[b59] Sasahara M, Otani A, Oishi A, Kojima H, Yodoi Y, Kameda T, Nakamura H, Yoshimura N (2008). Activation of bone marrow-derived microglia promotes photoreceptor survival in inherited retinal degeneration. Am J Pathol.

[b60] Silverman MD, Zamora DO, Pan Y, Texeira PV, Baek SH, Planck SR, Rosenbaum JT (2003). Constitutive and inflammatory mediator-regulated fractalkine expression in human ocular tissues and cultured cells. Invest Ophthalmol Vis Sci.

[b61] Simpson JE, Newcombe J, Cuzner ML, Woodroofe MN (1998). Expression of monocyte chemoattractant protein-1 and other beta-chemokines by resident glia and inflammatory cells in multiple sclerosis lesions. J Neuroimmunol.

[b62] Sunderkotter C, Nikolic T, Dillon MJ, Van Rooijen N, Stehling M, Drevets DA, Leenen PJ (2004). Subpopulations of mouse blood monocytes differ in maturation stage and inflammatory response. J Immunol.

[b63] Suzuki M, Tsujikawa M, Itabe H, Du ZJ, Xie P, Matsumura N, Fu X, Zhang R, Sonoda KH, Egashira K (2012). Chronic photo-oxidative stress and subsequent MCP-1 activation as causative factors for age-related macular degeneration. J Cell Sci.

[b64] Swirski FK, Libby P, Aikawa E, Alcaide P, Luscinskas FW, Weissleder R, Pittet MJ (2007). Ly-6Chi monocytes dominate hypercholesterolemia-associated monocytosis and give rise to macrophages in atheromata. J Clin Invest.

[b65] Tremblay ME, Stevens B, Sierra A, Wake H, Bessis A, Nimmerjahn A (2011). The role of microglia in the healthy brain. J Neurosci.

[b66] Tsou CL, Peters W, Si Y, Slaymaker S, Aslanian AM, Weisberg SP, Mack M, Charo IF (2007). Critical roles for CCR2 and MCP-3 in monocyte mobilization from bone marrow and recruitment to inflammatory sites. J Clin Invest.

[b67] Tsutsumi C, Sonoda KH, Egashira K, Qiao H, Hisatomi T, Nakao S, Ishibashi M, Charo IF, Sakamoto T, Murata T (2003). The critical role of ocular-infiltrating macrophages in the development of choroidal neovascularization. J Leukoc Biol.

[b68] Tuo J, Bojanowski CM, Zhou M, Shen D, Ross RJ, Rosenberg KI, Cameron DJ, Yin C, Kowalak JA, Zhuang Z (2007). Murine ccl2/cx3cr1 deficiency results in retinal lesions mimicking human age-related macular degeneration. Invest Ophthalmol Vis Sci.

[b69] Tuo J, Smith BC, Bojanowski CM, Meleth AD, Gery I, Csaky KG, Chew EY, Chan CC (2004). The involvement of sequence variation and expression of CX3CR1 in the pathogenesis of age-related macular degeneration. Faseb J.

[b70] van Leeuwen R, Klaver CC, Vingerling JR, Hofman A, de Jong PT (2003). The risk and natural course of age-related maculopathy: Follow-up at 6 1/2 years in the Rotterdam study. Arch Ophthalmol.

[b71] Vessey KA, Greferath U, Jobling AI, Phipps JA, Ho T, Waugh M, Fletcher EL (2012). Ccl2/Cx3cr1 knockout mice have inner retinal dysfunction but are not an accelerated model of AMD. Invest Ophthalmol Vis Sci.

[b72] Wong LM, Myers SJ, Tsou CL, Gosling J, Arai H, Charo IF (1997). Organization and differential expression of the human monocyte chemoattractant protein 1 receptor gene. Evidence for the role of the carboxyl-terminal tail in receptor trafficking. J Biol Chem.

[b73] Wynn TA, Chawla A, Pollard JW (2013). Macrophage biology in development, homeostasis and disease. Nature.

[b74] Xu H, Chen M, Manivannan A, Lois N, Forrester JV (2008). Age-dependent accumulation of lipofuscin in perivascular and subretinal microglia in experimental mice. Aging Cell.

[b75] Yamada K, Sakurai E, Itaya M, Yamasaki S, Ogura Y (2007). Inhibition of laser-induced choroidal neovascularization by atorvastatin by downregulation of monocyte chemotactic protein-1 synthesis in mice. Invest Ophthalmol Vis Sci.

[b76] Yang X, Hu J, Zhang J, Guan H (2010). Polymorphisms in CFH, HTRA1 and CX3CR1 confer risk to exudative age-related macular degeneration in Han Chinese. Br J Ophthalmol.

