# Supplementary material for: CCR2+ monocytes infiltrate atrophic lesions in age-related macular disease and mediate photoreceptor degeneration in experimental subretinal inflammation in Cx3cr1 deficient mice
Source: EMBO Mol Med. 2013 Oct 21;5(11):1775–93. doi: 10.1002/emmm.201302692 (PMC3840491; doi:10.1002/emmm.201302692)
Supplement: Supplementary file 1 [file emmm0005-1775-SD1.pdf]

# CCR2+ monocytes infiltrate atrophic lesions in age-related macular disease and mediate photoreceptor degeneration in experimental subretinal inflammation in Cx3cr1 deficient mice

Florian Sennlaub, Constance Auvynet, Bertrand Calippe, Sophie Lavalette, Lucie Poupel, Shulong J. Hu, Elisa Dominguez, Serge Camelo, Olivier Levy, Elodie Guyon, Noah Saederup, Israel F. Charo, Nico Van Rooijen, Emeline Nandrot, Jean-Louis Bourges, Francine Behar-Cohen, José-Alain Sahel, Xavier Guillonneau, William Raoul and Christophe Combadiere

*Corresponding authors: Florian Sennlaub, INSERM UMR S 968 and Christophe Combadière, Inserm UMR_945*

**Review timeline:**

| | |
|---|---|
| Submission date: | 28 February 2013 |
| Editorial Decision: | 08 April 2013 |
| Revision received: | 25 July 2013 |
| Editorial Decision: | 14 August 2013 |
| Revision received: | 28 August 2013 |
| Accepted: | 30 August 2013 |

## Transaction Report:

(Note: With the exception of the correction of typographical or spelling errors that could be a source of ambiguity, letters and reports are not edited. The original formatting of letters and referee reports may not be reflected in this compilation.)

*Editor: Roberto Buccione*

1st Editorial Decision 08 April 2013

Thank you for the submission of your manuscript to EMBO Molecular Medicine. We have now received reports from the three Reviewers whom we asked to evaluate your manuscript

You will see that the three Reviewers are generally supportive of your work albeit with a number of concerns that require your action. Altogether these concerns prevent us from considering publication at this time. I will not dwell into much detail, as the evaluations are self-explanatory. I would like, however, to highlight the main points.

Reviewer 1's concerns are mainly on issues of readability, quality of presentation and some lack of precision in phagocyte system definition. I agree that the manuscript in its current form would need some improvement in terms of readability and accuracy (as also noted by Reviewer 2).

Reviewer 2 is concerned that the mouse model of atrophic AMD does not faithfully reflect the human condition and suggests that the differences, especially with respect to the conclusions, should be carefully discussed. S/he would also like to see a better definition and explanation of the subretinal cellular populations that exist in the mouse models and through which signalling elements they interact with each other. Reviewer 2 mentions other issues that require your action and suggests

some improvements in layout and writing.

Reviewer 3, similarly to Reviewer 2, is concerned about the limitations of the mouse model and in this is respect feels that the conclusions are too strong; s/he points to some caveats that need to be taken into consideration. Reviewer 2 also notes other issues that need further work, among which 1) a better definition of the inflammatory markers present in the aqueous, 2) concern that the retinal explant assay leads to artefactual effects on monocyte activation and 3) mention of a possible drusen masking effect (also mentioned by Reviewer 2).

While publication of the paper cannot be considered at this stage, we would be prepared to consider a suitably revised submission, with the understanding that the Reviewers' concerns must be fully addressed with additional experimental data where appropriate and that acceptance of the manuscript will entail a second round of review.

Please note that it is EMBO Molecular Medicine policy to allow a single round of revision only and that, therefore, acceptance or rejection of the manuscript will depend on the completeness of your responses included in the next, final version of the manuscript.

As you know, EMBO Molecular Medicine has a "scooping protection" policy, whereby similar findings that are published by others during review or revision are not a criterion for rejection. However, I do ask you to get in touch with us after three months if you have not completed your revision, to update us on the status. Please also contact us as soon as possible if similar work is published elsewhere.

I look forward to seeing a revised form of your manuscript as soon as possible.

***** Reviewer's comments *****

Referee #1 (Comments on Novelty/Model System):

Sennlaub et al have performed logically constructed study in state-of-the art mouse model system. Infiltration of selected subsets of monocytes is a relevant topic since targeting of monocyte subsets opens perspectives for therapeutic immunomodulation in various inflammatory diosrcers.. The mechanistic aspect is adequately elaborated.

Referee #1 (Remarks):

Sennlaub et al have addressed relevant and medical important question about the role specific monocyte subsets in photoreceptor degeneration in experimental inflammatory animal model. The study is logically constructed and state-of the-art animal models are used. The study has no gaps and clearly demonstrates that interference with CCL2-mediate monocyte recruitment prevents photoreceptor degeneration in vivo. However, modifications for Introduction are commended

Minor points
1. Introduction is overloaded with information. It is strongly recommended to present clearly current gaps in our knowledge, precisely define open questions, and to summarise the reasons why current study was the best approach to answer these questions. It will make paper more understandable for broad scientific auditory. Extensive information about the experimental details of cited studies is better to replace to Discussion.

2. Introduction, page 3, second paragraph. The classification of mononuclear phagocyte system is not optimal here. Microglial cells are also tissue macrophages. In several tissues resident macrophages have historical specific names like Kupffer cells in liver and microglia in the brain. Microglia should be considered as subtype of tissue macrophages.

Referee #2 (Comments on Novelty/Model System):

This study examines a topic of interest in the field of age-related macular degeneration research: how inflammatory changes in the retina contribute to disease progression. Although mononuclear phagocytes (MPs) have been previously detected in AMD lesions, their expression of CCL2 and CCR2, which implicates their origin as infiltrating monocytes, is a novel point. Although the manuscript shows that CCL2/CCR2 signaling is required for monocytic infiltration and photoreceptor degeneration, the technical quality of the paper is impacted from an absence of data showing which cell types are engaged in this signaling, how CCR2 monocytes are recruited into the outer retina, and how they then upon entry induce photoreceptor degeneration. The transgenic mouse model system used in this study shares some features with the human disease but whether atrophy in human disease develops in the same way as that in the mouse model is only indirectly imputed (commented on below). The medical impact of this finding is significant as it indicates that CCL2/CCR2 signaling blockade may prevent monocytic retinal infiltration which in turn may limit photoreceptor degeneration.

Referee #2 (Remarks):

This manuscript explores the signaling mechanisms governing the recruitment of inflammatory cells to the retina and its effect on photoreceptor degeneration in a transgenic mouse model. The mechanisms examined in this model are interesting, as they may be potentially related to those involved in the pathogenesis of atrophic age-related macular degeneration (AMD).
The findings that (a) the recruitment of mononuclear phagocytes (MPs) to the subretinal space in aged and light-injured CX3CR1-/- mice is dependent on CCL2/CCR signaling, (b) that these MPs consist in part of CCR2+ monocytes, and (c) that these CCR2+ monocytes contribute to photoreceptor apoptosis, contribute significant data to an ongoing concern in the field of AMD research and are worthy of publication.
Major comments:
1. CX3CR1-deficient mice as a model for atrophic AMD: The authors have described some similarities in the outer retina of atrophic AMD patients and in aged and light-challenged CX3CR1-/- mice. They have found that in both mice and human retina contain a CCL2- and CCR2-positive inflammatory infiltrate and that ocular levels of CCL2 are increased. However, the causal influence of CCL2+ and CCR2+ MPs in atrophic AMD can only be indirectly extrapolated from the mouse data. Also a number of dissimilarities exist: a) these is no strong evidence for CX3CR1 deficiency/dysfunction in atrophic AMD, b) in the current study, aqueous levels of CX3CL1 are similar between atrophic AMD patients and controls, suggesting a preservation of CX3CR1 signaling in human disease, and c) atrophic AMD is very strongly associated with large drusen and RPE atrophy, features absent in aged or acutely light-challenged CX3CR1-/- mice. The possibility that inflammatory changes in atrophic AMD represent responses to, rather than causes of, photoreceptor degeneration in atrophic AMD remains a real one. Data demonstrating the presence of similar inflammatory changes during the precursor stages of atrophic AMD (i.e. at early and intermediate stages of AMD) would strengthen the notion that CCR2+ monocytes have a causal role in human disease. The title of the manuscript has juxtaposed these observations in AMD and this transgenic mouse model suggestively, however, a judicious critique of the strength/weakness of this imputed connection is welcomein the Discussion (i.e. that the processes observed in mouse and man share similarities but it remains possible that they are distinct in etiology and consequence).
2. Cellular entities and cellular mechanisms underlying MP infiltration and photoreceptor degeneration in aged and light-challenged CX3CR1-/- mice: The authors have invoked a number of cellular entities within the subretinal space that apparently interact with each other in different ways via CCL2/CCR2 and CX3CL1/CX3CR1 signaling. Some of these interactions seem complex, and the identities of the cellular entities involved in these interactions are not well discerned. For example, following light-challenge in CX3CR1-/- mice, the presence of CCL2 appears necessary for the recruitment of CCR2+ monocytes. However, the presence of CCR2 also seems necessary for the up-regulation of CCL2 expression in the retina. In this case, the source of CCL2 that initiates monocyte recruitment is not clear, nor is the way in which different cellular entities interact with each other to drive and perpetuate MP recruitment. Another point is that signals originating from the retina seem on one hand to be important in the regulation of monocyte expression of CCL2 (some

unknown retinal signal/s increases CCL2 expression, while retinal-derived CX3CL1 represses it). Conversely, unknown retinal signals also repress monocyte CCR2 expression in a CX3CR1-independent manner. These interactions are not incorporated into an overall picture/schema which will be helpful for understanding the proposed pathological mechanism. The authors in the Discussion also invoke the presence of a mixed population of MPs in the subretinal space ("The monocyte-depletion experiments confirm that inflammatory CCR2+ monocytes are recruited to the subretinal space and participate in subretinal MP accumulation by at least about 50%."). They also opine that each component of this mixed MP population exerts differing and opposite effects on photoreceptor degeneration. However, none of the data in the manuscript makes reference to the composition of this mixed population. This entire thesis of the manuscript can be better articulated if the authors can define and explain what subretinal cellular entities exist in the mouse models, and describe clearly how they signal to each other using CCL2/CCR2, CX3CL1/CX3CR1, or other channels. This may be done by: (a) defining quantitatively the potentially overlapping subsets of CCR2+, CCL2+, and CCR2-/CCL2- MPs existing in the subretinal space in both aging and light-challenged models and in human atrophic AMD, (b) clarifying whether the source of CCL2 in aging and light-challenged mice and in atrophic AMD is solely from infiltrating MPs or whether other retinal cells can also upregulate CCL2 expression (in Nakazawa et al., PNAS 2007, a retinal detachment model demonstrated that CCL2 was upregulated instead in Muller cells, which induced macrophage/microglia infiltration, that then in turn contribute to photoreceptor degeneration),(c) explaining why subretinal MPs are increased in CCL2-/- and CCR2-/- mice, and establishing how these MPs are similar or different from those in CX3CR1-/- mice, and (d) demonstrating how the absence of CX3CR1 signaling in monocytes results in such different responses compared to WT monocytes.

Minor points:

1. While the data is interesting and the figures are clear, the narrative and quality of writing can be improved. The "minireview" section as supplementary data is an uncommon narrative device, and the central points in the mini-review and their relevance to the main paper is not easily connected with the main points of the paper. The authors should consider either incorporating these points into the Discussion or keep the "minireview" but succinctly listing the consensus and discrepancies in findings described by previous studies on subretinal MPs in transgenic mouse models.

2. There are some published studies where the recruitment of circulating MP to the retina is not described being detrimental but instead thought to be helpful to retinal cell survival. These include Sasahara et al., Am J Pathology, 2008; Otani et al., JCI, 2004; the authors should reference them in the discussion and account for them in their overall depiction of disease mechanisms.

Referee #3 (Remarks):

General comments

This is an interesting study investigating the role od chemokine-ligand pairs CCR2-CCL2 and CX3CR1 in AMD. There are two main issues here: is there a good mouse model of geographic atrophy (dry AMD) and what is the role of inflammation in this disease. While the mouse is not a truly representative model of AMD since it does not have a macula or fovea, the main retinal damage in dry AMD is at the level of the retinal pigment epithelium (RPE) and models of progressive RPE atrophy associated with age (and light damage) do offer a working model for investigating this central defect in dry AMD. This paper uses such a model.

Concerning the second question of the role of inflammation in dry AMD it is less easy to attribute specific molecular evidence of direct pathogenesis in part because the process of inflammation is also revealing new discoveries. For instance, there is unquestionable evidence for a role for the chemokine-ligand pair CCL2-CCR2 in acute inflammatory responses, but in its absence a lower grade of inflammation still occurs suggesting that other chemokines may sustain a lower grade acute phase response while the requirements for perpetuation of chronic inflammation may be different or at least biased towards other chemokine-ligand interactions. This may also be determined by the type of mononuclear phagocyte involved in the tissue damage as outlined by the authors in the introduction to this paper.

Given this background, it is not surprising that there is some confusion in this field since different authors describe different models (eg dry, wet or both models of AMD). The data described in this paper show differences to those of Xu et al, Luhmann et al and Ambati et al, in part because the model here is aging of gene deficient mice in the context of a relatively acute light-damage insult,

which is different from those reported by the other groups. In addition, the specific animal housing conditions will have a bearing on the overall response.
In this context, the experiments reported here have been well performed (see some specific points which require to be addressed) and the data mostly are robust (see below).
The conclusions are however overly strong. Some consideration must be given to macrophage plastiticity; in particular, it is not exclusive that CCL2 is the main stimulator of macrophage damage and even when macrophages are depleted, subretinal accumulation of macrophages is only reduced by 50% (Fig 5k) there expression of CCR2 and response to CCL2 may not be the only means of causing tissue damage by macrophages and this is supported by previous work from others in which retinal damage occurs in the CCL2 and CCR2 KO mice. Despite these caveats, the authors have identified an interesting regulatory /protective role for CX3CR1 against damage by inflammatory macrophages whether they are CCR2+ or not.

Specific comments
The significance of the CCL2 staining and presence in the aqueous is not clear. What other chemokines or inflammatory markers were present?
Cells in Figure 1e reported to be neutrophils and lymphocytes are not definitively marked. Figures 1f and g are convincing, in part because of the number of cells expressing CCR2.
Figure 3f: the grey shading of the bars is not clear and it is difficult to differentiate the subsets of mice.
In Figure 3 the granularity of the fundi in 3h and 3j is less distinct but still visible. What is the retinal thickness in these mice? It si possible that there is some masking of the "pseudodrusen".
The retinal explant assay is questionable: it is well known that there is considerable retinal cell death (necrosis) after a few hours of culture associated with endogenous retinal microglial and perivascular macrophage activation. This is likely to have a considerable effect on monocyte activation.
Macrophage accumulation and tissue damage do not necessarily correlate: it is important to correlate macrophage accumulation with retinal damage quantitatively, for instance in Fig 5Kthere is still considerable macrophage accumulation but virtually no TUNEL positivity.
In Figure 6 it is unlikely that inhibition of CCR2+ macrophages is specific for CxCR1 macrophage damage. Was this agent tried in other forms of retinal damage?

1st Revision - authors' response 25 July 2013

The manuscript has been substantially revised following several series of new experiments corresponding to the reviewers' comments. These changes are detailed below :

• We have included CCR2 immunohistochemistry on sections from patients with age-related maculopathy (early AMD; Fig. 1G and H and supplementary data).

• In addition to the quantification of CCR2+ infiltrating monocytes, we have performed IBA-1 immunohistochemistry using a pan mononuclear phagocyte (MP) marker that also stains microglial cells (MCs)) on all sections from patients with geographic atrophy and quantified MP density in the inner retina and in the subretinal space for control and patient sections (Fig. 1N-P).

• We have also included the numerical results of CX3CL1 ELISA in aqueous humor from controls and patients with GA in the revised manuscript's text.

• To better characterize CCL2 expressing cells in the Cx3cr1$^{-/-}$ model, we cell sorted CCR2 expressing GFP$^{low}$Ly6C$^{high}$ infiltrating monocytes and GFP$^{high}$Ly6C$^{neg}$ resident Mϕs and MCs of PBS perfused Cx3cr1$^{GFP/GFP}$ mice after 4 days of light-challenge and compared Ccl2mRNA levels to whole-eye-lysats (Fig. 2I). Cells were sorted at 4 days of light-challenge, as Ly6C$^{high}$ cell sorting at d14 revealed an insufficient yield of cells. To verify that Ccl2 mRNA was elevated after 4 days of light-challenge we repeated the RT-PCR analysis of Ccl2 mRNA expression in wildtype and Cx3cr1$^{-/-}$mice to include d4 (Fig. 2B) and verified that Ccl2 induction was dependent on CCR2+monocyte recruitment in light-challenged Cx3cr1$^{GFP/GFP}$ mice and Cx3cr1$^{GFP/GFP}$Ccr2$^{RFP/RFP}$ mice at day 4 (Fig. 2H, the RT-PCR was performed at d14 in the first version).

• We have replaced in vitro monocyte Ccl2 and CCR2 expression in the retinal-explant co-culture system with experiments using purified photoreceptor outer segments (POS) to avoid the possible influence of dying cells in the retinal explant, as suggested by reviewer 3 (Fig. 2J and 5E).

• The quantification of subretinal MP accumulation in light-challenged C57BL/6, Ccl2$^{-/-}$, Cx3cr1$^{-/-}$, and Cx3cr1$^{-/-}$Ccl2$^{-/-}$ now also include a day 4 analysis. Fundoscopy photographs have been changed to a higher resolution to better appreciate retinal pseudodrusen.

• To better characterize the participation of monocyte-derived MPs in the light-induced subretinal accumulation we permanently marked circulating Mos using EdU injections and analysed their participation in the subretinal accumulation using clodronate and control liposomes at day 4.

• Photoreceptor toxicity of wildtype and Cx3cr1$^{-/-}$ Mos and MCs was evaluated on retinal explants (Fig. 6) to better define the subpopulations (Mo-deriver MPs versus MCs) toxic potentials.

• We have rewritten and simplified the introduction, incorporating the new results in the revised manuscript.

Finally, we would like to draw your attention to two manuscripts that further confirm the deleterious effect of monocyte recruitment in retinal degeneration in the carboxyethylpyrrole immunization induced AMD model (Cruz-Guilloty et al, 2013) and in the Abca4-/-Rdh8-/-mouse Stargardt/AMD model (Kohno et al, 2013). We believe that these recent publications will fall under EMBO Molecular Medicine's "scooping protection".

**Point-by-point response to the reviewers' comments**

Referee #1 (Comments on Novelty/Model System):

Sennlaub et al have performed logically constructed study in state-of-the art mouse model system. Infiltration of selected subsets of monocytes is a relevant topic since targeting of monocyte subsets opens perspectives for therapeutic immunomodulation in various inflammatory diosrcers.. The mechanistic aspect is adequately elaborated.

*We thank the reviewer for such supportive comments.*

Referee #1 (Remarks):

Sennlaub et al have addressed relevant and medical important question about the role specific monocyte subsets in photoreceptor degeneration in experimental inflammatory animal model. The study is logically constructed and state-of the-art animal models are used. The study has no gaps and clearly demonstrates that interference with CCL2-mediate monocyte recruitment prevents photoreceptor degeneration in vivo. However, modifications for Introduction are commended

Minor points

1. Introduction is overloaded with information. It is strongly recommended to present clearly current gaps in our knowledge, precisely define open questions, and to summarise the reasons why current study was the best approach to answer these questions. It will make paper more understandable for broad scientific auditory. Extensive information about the experimental details of cited studies is better to replace to Discussion.

*We agree with the reviewer that the introduction was overly complicated. It has been simplified as much as possible.*

2. Introduction, page 3, second paragraph. The classification of mononuclear phagocyte system is not optimal here. Microglial cells are also tissue macrophages. In several tissues resident macrophages have historical specific names like Kupffer cells in liver and microglia in the brain. Microglia should be considered as subtype of tissue macrophages.

*In the rewritten introduction, the mononuclear phagocyte (MP) definition was shortened and kept to a strict minimum. We agree with the reviewer that microglial cells (MC) are a type of resident macrophages. However, recent research shows that microglia derive from primitive myeloid progenitors at an earlier stage of development than other resident macrophages (Ginhoux et al, 2010). More importantly, MP transcriptome analysis has recently shown that microglia differ from other macrophage populations (Gautier et al, 2012). It is therefore likely that retinal MCs differ*

*significantly from resident macrophages of the ciliary body and the choroid. We therefore make a distinction between MCs and resident macrophages where it is strictly necessary.*

Referee #2 (Comments on Novelty/Model System):

This study examines a topic of interest in the field of age-related macular degeneration research: how inflammatory changes in the retina contribute to disease progression. Although mononuclear phagocytes (MPs) have been previously detected in AMD lesions, their expression of CCL2 and CCR2, which implicates their origin as infiltrating monocytes, is a novel point. Although the manuscript shows that CCL2/CCR2 signalling is required for monocytic infiltration and photoreceptor degeneration, the technical quality of the paper is impacted from an absence of data showing which cell types are engaged in this signalling, how CCR2 monocytes are recruited into the outer retina, and how they then upon entry induce photoreceptor degeneration. The transgenic mouse model system used in this study shares some features with the human disease but whether atrophy in human disease develops in the same way as that in the mouse model is only indirectly imputed (commented on below). The medical impact of this finding is significant as it indicates that CCL2/CCR2 signalling blockade may prevent monocytic retinal infiltration which in turn may limit photoreceptor degeneration.

Referee #2 (Remarks):

This manuscript explores the signalling mechanisms governing the recruitment of inflammatory cells to the retina and its effect on photoreceptor degeneration in a transgenic mouse model. The mechanisms examined in this model are interesting, as they may be potentially related to those involved in the pathogenesis of atrophic age-related macular degeneration (AMD).

The findings that (a) the recruitment of mononuclear phagocytes (MPs) to the subretinal space in aged and light-injured CX3CR1-/- mice is dependent on CCL2/CCR signalling, (b) that these MPs consist in part of CCR2+ monocytes, and (c) that these CCR2+ monocytes contribute to photoreceptor apoptosis, contribute significant data to an on-going concern in the field of AMD research and are worthy of publication.

Major comments:

1. CX3CR1-deficient mice as a model for atrophic AMD: The authors have described some similarities in the outer retina of atrophic AMD patients and in aged and light-challenged CX3CR1-/- mice. They have found that in both mice and human retina contain a CCL2- and CCR2-positive inflammatory infiltrate and that ocular levels of CCL2 are increased. However, the causal influence of CCL2+ and CCR2+ MPs in atrophic AMD can only be indirectly extrapolated from the mouse data. Also a number of dissimilarities exist: a) these is no strong evidence for CX3CR1 deficiency/dysfunction in atrophic AMD, b) in the current study, aqueous levels of CX3CL1 are similar between atrophic AMD patients and controls, suggesting a preservation of CX3CR1 signalling in human disease, and c) atrophic AMD is very strongly associated with large drusen and RPE atrophy, features absent in aged or acutely light-challenged CX3CR1-/- mice. The possibility that inflammatory changes in atrophic AMD represent responses to, rather than causes of, photoreceptor degeneration in atrophic AMD remains a real one. Data demonstrating the presence of similar inflammatory changes during the precursor stages of atrophic AMD (i.e. at early and intermediate stages of AMD) would strengthen the notion that CCR2+ monocytes have a causal role in human disease.

*We agree with the reviewer that CX3CR1$^{-/-}$ mice are a model that only mimics certain aspects found in GA (CCL2 increase, monocyte infiltration and subretinal MP accumulation and associated photoreceptor degeneration). The CX3CR1$^{-/-}$ mouse model can therefore only be seen as a part of a much wider picture of other models that mimic (also incompletely) different aspects of GA. Over the last two years there has been an increasing interest in monocyte recruitment and its deleterious effect on photoreceptor survival, in photo-oxydative stress models (Rutar et al, 2012; Suzuki et al, 2012), the Abca4-/-Rdh8-/-mouse Stargardt/AMD model (Kohno et al, 2013), in a carboxyethylpyrrole immunization induced AMD model (Cruz-Guilloty et al, 2013), and in rd1 mice (Guo et al, 2012). Our manuscript takes part in the effort to analyse the effect of CCR2+monocyte recruitment in retinal degeneration and is the first to describe CCL2 increase and CCR2+ monocyte-derived MP infiltration in GA.*

*We agree with the reviewer, that there is no evidence for an association of a genetic variation with atrophic AMD and that the manuscript poorly explained why we think that the CX3CR1$^{-/-}$ mouse model is a valuable addition to the models used to decipher the influence of monocyte recruitment in*

*photoreceptor degeneration and in particular GA. To better explain the concept, a new paragraph has been added to the discussion of the new manuscript (see below).*

*Concerning the lack of a difference in CX3CL1 levels in GA patients, it must be kept in mind that, unlike CCL2, CX3CL1 is constitutively expressed as a transmembrane protein in retinal neurons (Silverman et al, 2003). The levels of soluble cleaved CX3CL1 in the aqueous humor of control patients is very low and does not seem to reflect the strong constitutive expression of transmembraneous CX3CL1 in the retina. In this context, it is problematic to conclude from the absence of a difference in GA patients that CX3CR1 signalling is preserved. We do not claim to show that CX3CL1/CX3CR1 signalling is affected in GA. However, we do believe that the Cx3cr1*$^{-/-}$ *model is a valuable addition to other models, for the reasons cited above (CCL2 increase, monocyte infiltration, subretinal MP accumulation, and associated photoreceptor degeneration).*

*Numerical values of CX3CL1 measurements were added to the new manuscript in the result section and discussed in the discussion of the new manuscript (see below).*

*As pointed out by the reviewer, age related maculopathy (ARM) is a strong risk factor for GA and wet AMD. As suggested, we analysed CCR2 immunohistochemistry in 4 eyes from 3 patients with AML (laminar deposits and soft drusen (>160mm in size) in the absence of geographic lesions or neovascularization). CCR2+monocyte infiltration of laminar deposits and soft drusen were found in all eyes examined and these results were included in Fig. 1 (more examples are shown in the supplementary data). This data suggests that CCR2+monocyte infiltration is present in ARM.*

*We would also like to point out that we agree that CCR2+ monocyte infiltration represents a response to lipid accumulation and photoreceptor degeneration in ARM and GA, a point we stress throughout the manuscript. However, we do also think that there is good evidence from our data that the CCR2+ monocyte infiltration can attract other monocytes and that the infiltration of CCR2+ monocytes significantly contributes to the photoreceptor degeneration, at least in aged and light-challenged Cx3cr1*$^{-/-}$*mice. Models of light-, genetically-induced and autoimmune-photoreceptor/RPE degeneration seem to suggest that this is the case in other models (Cruz-Guilloty et al, 2013; Kohno et al, 2013; Rutar et al, 2012; Suzuki et al, 2012)*

The title of the manuscript has juxtaposed these observations in AMD and this transgenic mouse model suggestively, however, a judicious critique of the strength/weakness of this imputed connection is welcome in the Discussion (i.e. that the processes observed in mouse and man share similarities but it remains possible that they are distinct in etiology and consequence).

*A new paragraph was added to the discussion explaining similarities and discrepancies of the CX3CR1*$^{-/-}$ *mouse model and the human disease. We also point out the model's limitations throughout the manuscript.*

*Page 23: "Cx3cr1 polymorphisms have been associated with wet AMD in some studies (Anastasopoulos et al, 2012, 2012 #1778; Combadiere et al, 2007; Tuo et al, 2004; Yang et al, 2010), but their possible involvement in GA is unknown. Unlike CCL2, CX3CL1 is constitutively expressed as a transmembrane protein in retinal neurons (Silverman et al, 2003) and is known to provide a tonic inhibitory signal to CX3CR1 bearing MCs that keeps these cells in a quiescent surveillance mode in the brain (Ransohoff, 2009). CX3CL1 levels in the aqueous humor of controls were very low; very little cleaved CX3CL1 seems to reach the aqueous humor under physiological conditions and no difference was observed in the GA group. However, the loss of CX3CL1/CX3CR1 signalling in the retina, leads to a strong increase of subretinal MP accumulation, as observed in Cx3cr1-deficient mice with age and after a light-challenge compared to wildtype mice. Importantly, the accumulation of subretinal Cx3cr1-deficient MPs is associated with photoreceptor degeneration (Combadiere et al, 2007; Ma et al, 2009; Raoul et al, 2008a). Although they do not mimic all aspects of AMD (drusen formation and RPE atrophy) they do model subretinal MP accumulation and associated photoreceptor degeneration, which are hallmarks of AMD (Combadiere et al, 2007; Gupta et al, 2003). Compared to other models in which subretinal inflammation occurs secondarily to light-injury or genetic defects (Cruz-Guilloty et al, 2013; Guo et al, 2012; Kohno et al, 2013; Rutar et al, 2012; Suzuki et al, 2012), the Cx3cr1 knockout mouse model presents a primary MP accumulation in the absence of an inherited retinal degeneration (C57BL/6J background) (Combadiere et al, 2007). The absence of confounding factors that lead to photoreceptor cell death in the Cx3cr1 knockout mouse model makes it a particularly suitable model for the study of subretinal inflammation and its influence on photoreceptor homeostasis (note that the intensity of the light-challenge model used herein was developed to induce subretinal inflammation and subsequent photoreceptor degeneration in the Cx3cr1*$^{-/-}$ *mice but not in Cx3cr1*$^{+/+}$ *mice (see below)).*

*The light intensity used in these experiments is not strong enough to directly induce photoreceptor apoptosis in pigmented wildtype animals, contrary to classically used light-injury models)."*

2. Cellular entities and cellular mechanisms underlying MP infiltration and photoreceptor degeneration in aged and light-challenged CX3CR1-/- mice: The authors have invoked a number of cellular entities within the subretinal space that apparently interact with each other in different ways via CCL2/CCR2 and CX3CL1/CX3CR1 signalling. Some of these interactions seem complex, and the identities of the cellular entities involved in these interactions are not well discerned. For example, following light-challenge in CX3CR1-/- mice, the presence of CCL2 appears necessary for the recruitment of CCR2+ monocytes. However, the presence of CCR2 also seems necessary for the up-regulation of CCL2 expression in the retina. In this case, the source of CCL2 that initiates monocyte recruitment is not clear, nor is the way in which different cellular entities interact with each other to drive and perpetuate MP recruitment. Another point is that signals originating from the retina seem on one hand to be important in the regulation of monocyte expression of CCL2 (some unknown retinal signal/s increases CCL2 expression, while retinal-derived CX3CL1 represses it). Conversely, unknown retinal signals also repress monocyte CCR2 expression in a CX3CR1-independent manner. These interactions are not incorporated into an overall picture/schema which will be helpful for understanding the proposed pathological mechanism.

The authors in the Discussion also invoke the presence of a mixed population of MPs in the subretinal space ("The monocyte-depletion experiments confirm that inflammatory CCR2+ monocytes are recruited to the subretinal space and participate in subretinal MP accumulation by at least about 50%."). They also opine that each component of this mixed MP population exerts differing and opposite effects on photoreceptor degeneration. However, none of the data in the manuscript makes reference to the composition of this mixed population.

This entire thesis of the manuscript can be better articulated if the authors can define and explain what subretinal cellular entities exist in the mouse models, and describe clearly how they signal to each other using CCL2/CCR2, CX3CL1/CX3CR1, or other channels.

*To better evaluate the extent of $CCR2^{+}$ Mos in subretinal MP accumulation we have conducted a new series of experiments. We permanently marked the circulating monocytes with repeated EdU injections prior to and during light-induced subretinal recruitment in $Cx3cr1^{-/-}$ mice and compared the numbers of subretinal $EdU^{+}$ MPs in mice with and without clodronate-liposome-induced circulating monocyte depletion. Local EdU administration failed to mark subretinal MPs, suggesting that ocular MP proliferation does not play a significant role in the light-induced accumulation. In these experiments 37% of subretinal MPs were EdU positive in mice receiving systemic EdU injections and control liposomes, which seems to suggest that at least one third of subretinal MPs are derived from monocytes (Fig. 5). Considering that the EdU injections only marked 76% of circulating monocytes, the extent of Mo participation in light-induced subretinal MP accumulation using this method is possibly underestimated. Indeed, monocyte depletion inhibited the subretinal $IBA\text{-}1^{+}$ MP accumulation by 60 to 50% (Fig. 5), suggesting that as much as half of the subretinal MP population might originate from blood-borne Mo. (The other half being local resident Mfs or MCs.) Similarly, genetic Ccl2 or Ccr2 deletion and CCR2 inhibitors diminished subretinal MP accumulation by 50-60% in age- and light-challenge-induced in $Cx3cr1^{-/-}$and $Cx3cr1^{GFP/GFP}$ (Fig. 2, 3 and 7). The new results are presented in (Fig. 5I-L).*

*Interestingly, the inhibition of $CCR2^{+}$ monocyte recruitment (Ccl2 or Ccr2 deletion, monocyte depletion and pharmacological inhibition Fig. 3, 4, 5 and 7) nearly completely inhibited photoreceptor degeneration in $Cx3cr1^{-/-}$ mice. To better define the level of photoreceptor toxicity of Mo-derived MPs and MCs, we performed a new series of in vitro co-culture experiments. The photoreceptor toxicity of wildtype and $Cx3cr1^{-/-}$ Mos and MCs was evaluated on retinal explants (Fig. 6). Our results show that Mos and particularly $Cx3cr1^{-/-}$ Mos display important photoreceptor toxicity when compared to MCs. This might explain the marked neuroprotective effect of monocyte depletion in light-challenged $Cx3cr1^{-/-}$ mice.*

*To evaluate whether CX3CR1 influences the level of CCL2 expression in Mϕs in the subretinal microenvironment, we incubated $Cx3cr1^{+/+}$ and $Cx3cr1^{-/-}$ macrophages derived from purified bone marrow monocytes (bMo) with photoreceptor outer segments (POS) prepared from pig retina. (The previous manuscript contained similar results using retinal explants that were discarded because reviewer 3 pointed out that dying cells of the explants might influence the results). We show that Ccl2 mRNA expression (Fig. 2J) is comparable in control $Cx3cr1^{+/+}$ and $Cx3cr1^{-/-}$ Mϕs at 18h of*

*culture. When the Mϕs were derived in the presence of POS, Ccl2 mRNA induction in Cx3cr1$^{-/-}$ Mϕs was significantly stronger compared to Cx3cr1$^{+/+}$ Mϕs (Fig. 2J) suggesting that CX3CR1 signalling helps repress CCL2 secretion in subretinal wild type Mϕs.*

*As a whole, the revised manuscript provides further evidence that CCL2 produced by subretinal Cx3cr1$^{-/-}$ Mϕs participates in the recruitment of CCR2+ monocytes that, in turn, participate in subretinal accumulation and photoreceptor degeneration in Cx3cr1$^{-/-}$ mice.*

This may be done by:

(a) defining quantitatively the potentially overlapping subsets of CCR2+, CCL2+, and CCR2-/CCL2- MPs existing in the subretinal space in both aging and light-challenged models and in human atrophic AMD,

*In the original manuscript, we showed that CCL2 immunohistochemically labelled subretinal MPs but not intraretinal MCs at day 14 of light challenged Cx3cr1$^{-/-}$ mice and that the significant Ccl2 mRNA induction observed in Cx3cr1$^{GFP/GFP}$ mice (similarly to Cx3cr1$^{-/-}$ mice) was completely prevented in Cx3cr1$^{GFP/GFP}$Ccr2$^{RFP/RFP}$ mice in which the accumulation is inhibited.*

*iMos express high levels of CCR2 and Ly6C and low levels of CX3CR1, while MCs and resident macrophages express low levels of CCR2 and Ly6C and high levels of CX3CR1 (Ransohoff & Cardona, 2010; Wynn et al, 2013). To better characterize CCL2 expressing MPs in the Cx3cr1$^{-/-}$ model, we cell sorted CCR2$^{+}$GFP$^{low}$Ly6C$^{high}$ infiltrating monocytes and GFP$^{high}$Ly6C$^{neg}$ resident Mϕs and MCs of PBS perfused Cx3cr1$^{GFP/GFP}$ mice after 4 days of light-challenge and compared Ccl2mRNA levels to whole-eye-lysats. Our results show that Ccl2 mRNA is strongly enriched in cell-sorted iMos in eyes of light-challenged Cx3cr1$^{-/-}$ mice (Fig. 2I). (Cells were sorted at 4 days of light-challenge, as Ly6C$^{high}$ cell sorting at d14 revealed an insufficient yield of cells. Similarly, cell sorting in aged Cx3cr1$^{-/-}$ mice, in which the subretinal MP accumulation occurs over months, resulted in insufficient cell numbers to perform RT-PCR.)*

*To verify that Ccl2 mRNA was elevated after 4 days of light-challenge we repeated the RT-PCR analysis of Ccl2 mRNA expression in wildtype and Cx3cr1$^{-/-}$mice to include d4 (Fig. 2B) and verified that Ccl2 induction was dependent on CCR2+monocyte recruitment in light-challenged Cx3cr1$^{GFP/GFP}$ mice and Cx3cr1$^{GFP/GFP}$Ccr2$^{RFP/RFP}$ mice at day 4 (Fig. 2H, the RT-PCR was performed at d14 in the previous version). We also show that Ccl2 induction is increased in Cx3cr1$^{-/-}$ monocyte-derived Mf in vitro differentiated in the presence of photoreceptor outer segments.*

*CX3CR1/CX3CL1 signalling has been shown to physiologically repress the expression of inflammatory mediators in CNS pathology and to protect neurons in inflammatory conditions (Cardona et al, 2006; Ransohoff, 2009). Our results show that CCL2 is overexpressed in subretinal MPs in Cx3cr1$^{-/-}$ mice and in monocyte-derived macrophages in the presence of POS in vitro. The accumulation of CCL2 overexpressing Cx3cr1$^{-/-}$ Mos might thereby lead to a positive feedback in aged- and light-challenged Cx3cr1$^{-/-}$ mice, after an initial recruitment through other chemotactic agents.*

(b) clarifying whether the source of CCL2 in aging and light-challenged mice and in atrophic AMD is solely from infiltrating MPs or whether other retinal cells can also upregulate CCL2 expression (in Nakazawa et al., PNAS 2007, a retinal detachment model demonstrated that CCL2 was upregulated instead in Muller cells, which induced macrophage/microglia infiltration, that then in turn contribute to photoreceptor degeneration),

*The results of our new experiments show that Ccl2 mRNA is strongly enriched in cell-sorted iMos in eyes of light-challenged Cx3cr1$^{-/-}$. We show that Ccl2 induction in light-challenged Cx3cr1$^{GFP/GFP}$ is inhibited in Cx3cr1$^{GFP/GFP}$Ccr2$^{RFP/RFP}$ mice, that Cx3cr1$^{-/-}$ Mos overexpress Ccl2 mRNA compared to wildtype Mos in the presence of POS (that are abundant in the subretinal space), and that CCL2 localizes to subretinal MPs in aged and light-challenged Cx3cr1$^{-/-}$ mice (where infiltrating Mos are located in light-challenged mice). CCL2 and activated Mϕs have been described to release CCL2 in neuro-inflammatory conditions such as multiple sclerosis in vivo (Simpson et al, 1998). In our immunohistochemical experiments in light-challenged and aged Cx3cr1$^{-/-}$ mice (or in GA), the CCL2 signal was not distributed in a Müller cell-like distribution or in the RPE. We do not question that CCL2 can be induced in Müller cells in models of retinal detachment or in light-injury (Nakazawa et al, 2007; Rutar et al, 2011). However, these are very different models from our aging- or light-challenged Cx3cr1$^{-/-}$ mouse models. The stimulus in retinal detachment and light-injury models is much stronger and induces a phenotype in wildtype animals. As mentioned before, in our model, the*

*light-challenge is not sufficient to induce retinal degeneration in wildtype mice, which might explain the differences observed in the CCL2 expressing cell populations.*

*We hope that our results presented in the new Fig. 2 will convince the reviewer that subretinal MPs are the main producers of CCL2 cells in the $Cx3cr1^{-/-}$mice.*

(c) explaining why subretinal MPs are increased in CCL2-/- and CCR2-/- mice, and establishing how these MPs are similar or different from those in CX3CR1-/- mice, and

*For the Cx3cr1 model we here show that CCL2 is over-expressed in $Cx3cr1^{-/-}$ mice, which leads to the influx of CCR2+ monocyte recruitment. We reported that $Cx3cr1^{-/-}$ Mfs present a clearance from the subretinal space compared to wildtype Mfs {Levy, 2011 #2067} We believe that the observed pathological MP accumulation in $Cx3cr1^{-/-}$ mice is the result of a combination of increased Mo recruitment and decreased MP clearance (see discussion of the manuscript)*

*For Ccl2 and Ccr2 the question is more difficult and none of the previously published articles that describe subretinal MP accumulation in Ccl2 or Ccr2 deficient mice have attempted to answer it. $Ccr2^{-/-}$ mice have been described to have perturbations of other chemokine signalling pathways {Cardona, 2008 #2060} and a similar mechanism might be implicated in subretinal MP accumulation described in these mice.*

*In our laboratory, the accumulation of subretinal MPs in 18 month-old $Ccl2^{-/-}$ mice was much closer to that observed in wildtype mice than to the accumulation observed in $Cx3cr1^{-/-}$kept in the same animal housing conditions (to illustrate this we present this data in the supplementary data with a detailed literature analysis that shows that subretinal MP accumulation is generally much lower than that observed in Cx3cr1 deficiency in the different laboratories).*

*Furthermore, as we describe in this manuscript, the inhibition of CCR2+ monocyte recruitment significantly inhibits the subretinal MP accumulation in $Cx3cr1^{-/-}$ mice. In our laboratory the accumulation of subretinal MPs in $Ccl2^{-/-}$mice are is minor when compared to $Cx3cr1^{-/-}$ mice. Clinical and experimental data suggest that elevated CCL2 expression (and not its deficiency) contributes to AMD pathogenesis. Deciphering the molecular mechanisms of the discrete subretinal MP accumulation in Ccl2 or Ccr2 deficient mice is not within the scope of this manuscript.*

*To summarize the current state of knowledge without becoming too hypothetical, we have added the following paragraph to the discussion:*

*Page 26: "Aged $Ccl2^{-/-}$ and $Ccr2^{-/-}$ mice have also been described to develop the more discrete subretinal MP accumulation that occurs in older mice compared to Cx3cr1 deficient mice, associated with little to no photoreceptor degeneration (see supplementary data) (Chen et al, 2011; Luhmann et al, 2009). It is not clear how Ccl2 and Ccr2 deficiency lead to subretinal MP accumulation, but subretinal $IBA\text{-}1^{+}$cells in aged $Ccl2^{-/-}$ and $Ccr2^{-/-}$ mice are likely not derived from $CCR2^{+}$ monocytes but may at least in part be $CCR2^{-}CX3CR1^{high}$ MCs and/or the non-inflammatory $CX3CR1^{+}$ monocytes, which could explain the absence of photoreceptor degeneration.*

*In summary, contrary to CCR2 and CCL2, CX3CR1 and CX3CL1 are constitutively expressed in the retina similar to the brain (Ransohoff & Cardona, 2010). CX3CR1 signalling suppresses the expression of inflammatory mediators, such as CCL2 in $CCR2^{+}$ monocyte derived inflammatory MPs in the subretinal microenvironment. Contrary to wildtype mice, age- and light-induced stress in Cx3cr1 deficient animals are sufficient to induce CCL2 expression and $CCR2^{+}$ monocyte recruitment to the subretinal space at a rate exceeding that of their clearance, and subretinal MPs accumulate. In the brain, $Cx3cr1^{-/-}$ Mfs clear less efficiently from the injection site compared to wildtype macrophages (Cardona et al, 2006). We reported that $Cx3cr1^{-/-}$ Mfs present a similar impaired clearance from the subretinal space compared to wildtype Mfs (Levy et al, 2011) and are currently investigating the molecular mechanism behind this phenomenon. The combination of increased Mo recruitment and decreased MP clearance possibly explains the pathological MP accumulation observed in $Cx3cr1^{-/-}$ mice. This pathological accumulation, in combination with the increased neurotoxicity of $Cx3cr1^{-/-}$ MPs (Cardona et al, 2006) and in particular $Cx3cr1^{-/-}$ Mos (Fig. 6) might explain the subretinal inflammation and photoreceptor degeneration observed in $Cx3cr1^{-/-}$ mice."*

(d) demonstrating how the absence of CX3CR1 signalling in monocytes results in such different responses compared to WT monocytes.

*To better define how CX3CR1 signalling on MCs and monocyte-derived Mfs affects photoreceptor toxicity we performed a new series of in vitro co-culture experiments on retinal explants, as*

*described above (Fig. 6). The nature of the phototoxic MP-derived factor is currently unknown and we (among others) are actively pursuing this research. Research papers in the field often present differences in a selection of "M1" markers, to illustrate their toxic potential. Macrophage polarization is considerably diverse and is highly dependent on its environment (Gautier et al, 2012; Wynn et al, 2013). As long as the Mf-derived photoreceptor toxic factor remains unidentified, we believe that our in vitro evaluation of photoreceptor-toxicity is more telling than the expression of classically described M1 markers.*

Minor points:

1. While the data is interesting and the figures are clear, the narrative and quality of writing can be improved. The "minireview" section as supplementary data is an uncommon narrative device, and the central points in the mini-review and their relevance to the main paper is not easily connected with the main points of the paper. The authors should consider either incorporating these points into the Discussion or keep the "minireview" but succinctly listing the consensus and discrepancies in findings described by previous studies on subretinal MPs in transgenic mouse models.

*As suggested by the reviewer we deleted the mini review from the supplementary data. Our comparative data of subretinal MP accumulation in 18 month-old Cx3cr1-/- and CCL2-/-mice is now presented under the heading: "Comparison of subretinal MP accumulation in C57BL/6, Ccl2-/- and Cx3cr1-/- mice at 18 months under the same animal housing conditions." (supplementary data) and accompanied by a detailed literature analysis for the interested reader.*

2. There are some published studies where the recruitment of circulating MP to the retina is not described being detrimental but instead thought to be helpful to retinal cell survival. These include Sasahara et al., Am J Pathology, 2008;

*We thank the reviewer for this suggestion and have included this reference in the discussion.*

Otani et al., JCI, 2004; the authors should reference them in the discussion and account for them in their overall depiction of disease mechanisms.

*Otani et al. describes the effect of lineage-negative hematopoietic stem cells containing endothelial precursors on vascular and retinal degeneration in rd1 mice. It does not investigate the role of Mos or MPs in retinal degeneration. We felt that a discussion of the influence of other bone-marrow derived cells on photoreceptor degeneration would be beyond the scope of our discussion.*

Referee #3 (Remarks):

General comments

This is an interesting study investigating the role od chemokine-ligand pairs CCR2-CCL2 and CX3CR1 in AMD. There are two main issues here: is there a good mouse model of geographic atrophy (dry AMD) and what is the role of inflammation in this disease. While the mouse is not a truly representative model of AMD since it does not have a macula or fovea, the main retinal damage in dry AMD is at the level of the retinal pigment epithelium (RPE) and models of progressive RPE atrophy associated with age (and light damage) do offer a working model for investigating this central defect in dry AMD. This paper uses such a model.

Concerning the second question of the role of inflammation in dry AMD it is less easy to attribute specific molecular evidence of direct pathogenesis in part because the process of inflammation is also revealing new discoveries. For instance, there is unquestionable evidence for a role for the chemokine-ligand pair CCL2-CCR2 in acute inflammatory responses, but in its absence a lower grade of inflammation still occurs suggesting that other chemokines may sustain a lower grade acute phase response while the requirements for perpetuation of chronic inflammation may be different or at least biased towards other chemokine-ligand interactions. This may also be determined by the type of mononuclear phagocyte involved in the tissue damage as outlined by the authors in the introduction to this paper.

Given this background, it is not surprising that there is some confusion in this field since different authors describe different models (e.g. dry, wet or both models of AMD). The data described in this paper show differences to those of Xu et al, Luhmann et al and Ambati et al, in part because the model here is aging of gene deficient mice in the context of a relatively acute light-damage insult,

which is different from those reported by the other groups. In addition, the specific animal housing conditions will have a bearing on the overall response.

In this context, the experiments reported here have been well performed (see some specific points which require to be addressed) and the data mostly are robust (see below).

The conclusions are however overly strong. Some consideration must be given to macrophage plasticity; in particular, it is not exclusive that CCL2 is the main stimulator of macrophage damage and even when macrophages are depleted, subretinal accumulation of macrophages is only reduced by 50% (Fig 5k) there expression of CCR2 and response to CCL2 may not be the only means of causing tissue damage by macrophages and this is supported by previous work from others in which retinal damage occurs in the CCL2 and CCR2 KO mice. Despite these caveats, the authors have identified an interesting regulatory /protective role for CX3CR1 against damage by inflammatory macrophages whether they are CCR2+ or not.

*We thank the reviewer for such supportive comments.*

Specific comments

The significance of the CCL2 staining and presence in the aqueous is not clear. What other chemokines or inflammatory markers were present?

*The chemokines we studied in the aqueous humour (AH) were CCL2 and CX3CL1. They were both measured using specific ELISAs that necessitated 50 ml of undiluted AH (cataract surgery AH specimens rarely exceeded 100ml per patient). Using ELISAs we were only able to measure the two chemokines (CCL2 and CX3CL1) that are of direct interest in this study. In the new manuscript, we have included the results from the CX3CL1 measurements that were very low and similar in both groups (see above) (Controls: 0,08 ng/ml +/-0,004 SEM; GA: 0,085 ng/ml +/-0,003 SEM). We therefore do not have measurements of other chemokines to present at this point. However, to better characterize the MP populations in GA we performed IBA1 staining on the paraffin sections of GA donors and controls that quantitatively confirm the accumulation of subretinal MPs in the subretinal space and show that there is no difference in numbers in terms of intraretinal MPs between the two groups (Fig. 1 M-O).*

Cells in Figure 1e reported to be neutrophils and lymphocytes are not definitively marked.

*We agree with the reviewer that the inset of the former Fig. 1E to illustrate CCR2 staining on blood smears was too small to allow for the morphological appreciation of polynuclear neutrophils and small round lymphocytes. Two micrographs depicting CCR2 positive monocytes alongside morphologically recognizable neutrophils (small polymorphnuclear) and lymphocytes (small and little cytoplasm) are therefore shown in the supplementary data to assure a better understanding.*

Figures 1f and g are convincing, in part because of the number of cells expressing CCR2.

*We agree with the reviewer that the former Fig. 1F contained more CCR2+ cells than counted on average in the GA lesions. The CCR2+ cells are not always evenly distributed in the GA lesion. We exchanged the picture Fig. 1F and also present 3 more CCR2 stained GA lesions in a figure in the supplementary data to illustrate different aspects of GA lesion presentation.*

Figure 3f: the grey shading of the bars is not clear and it is difficult to differentiate the subsets of mice.

*The new Figure 3 now contains a separate quantification of subretinal MP accumulation in light-challenged C57BL/6, $Ccl2^{-/-}$, $Cx3cr1^{-/-}$, and $Cx3cr1^{-/-}Ccl2^{-/-}$, that also includes a day4 analysis (Fig. 3F) and a separate graph to present the results for light-challenged C57BL/6J, $Ccl2^{-/-}$, $CCR2^{-/-}$, $Cx3cr1^{GFP/GFP}$, $Cx3cr1^{GFP/GFP}Ccl2^{-/-}$, and $Cx3cr1^{GFP/GFP}Ccr2^{RFP/RFP}$ mice (Fig. 3G). We have introduced colour coding to Fig. 3G to facilitate the differentiation of the subsets.*

In Figure 3 the granularity of the fundi in 3h and 3j is less distinct but still visible. What is the retinal thickness in these mice? It is possible that there is some masking of the "pseudodrusen".

*We agree with the referee that the wide-angle view of the fundus photographs does not facilitate the visualization of "pseudodrusen" in the figure. We changed all photographs of fundus pictures to a higher magnification in the new manuscript (in a previous submission we were asked to show wide-angle pictures because a reviewer suspected we wanted to hide the retinal lesions described in $Cx3cr1^{-/-}Ccl2^{-/-}$mice, that we now know are rd8 related.) We would also like to point out that although it is difficult to take fundus pictures that are sharp throughout the fundus, the*

*"pseudodrusen" are quite easy to see upon clinical observation. We are not trying to hide a phenotype in Ccl2-/- or Cx3cr1$^{-/-}$Ccl2$^{-/-}$mice. Total retinal thicknesses (that could lead to a masking effect) are not increased in Ccl2-/- or Cx3cr1$^{-/-}$Ccl2$^{-/-}$mice compared to wildtype animals. Please note that the "pseudodrusen" in these models correspond to "lipid bloated" subretinal MPs (Combadiere et al, 2007; Luhmann et al, 2009). Quantification of subretinal MPs (that are composed of "lipid bloated" and "lean" MPs) are certainly a more reliable way to evaluate the number of subretinal MPs.*

The retinal explant assay is questionable: it is well known that there is considerable retinal cell death (necrosis) after a few hours of culture associated with endogenous retinal microglial and perivascular macrophage activation. This is likely to have a considerable effect on monocyte activation.

*We agree with the referee that the retinal explant could influence gene expression in monocytes by means other than the presence of photoreceptor outer segments (POS). The in vitro monocyte Ccl2 and CCR2 expression in the retinal-explant co-culture system were replaced by experiments using purified photoreceptor outer segments (POS) (Fig. 2J and 5E).*

Macrophage accumulation and tissue damage do not necessarily correlate: it is important to correlate macrophage accumulation with retinal damage quantitatively, for instance in Fig 5Kthere is still considerable macrophage accumulation but virtually no TUNEL positivity.

*We agree with the reviewer that the inhibition of CCR2$^{+}$ monocyte recruitment unproportionally inhibited photoreceptor degeneration in Cx3cr1$^{-/-}$ mice (Ccl2 or Ccr2 deletion, monocyte depletion, and pharmacological inhibition new Fig. 3, 4, 5 and 7). The experiments presented in the former version of the manuscript (monocyte depletion experiments) only indirectly allowed the estimation of the participation of blood-derived monocytes in subretinal MP accumulation. As CCR2 expression is quickly downregulated in infiltrating Mos the Cx3cr1$^{GFP/GFP}$Ccr2$^{RFP/+}$ mice do not accurately reflect the Mo-derived MP population.*

*In the new manuscript we conducted a series of experiments to better evaluate the extent of CCR2$^{+}$ Mos in subretinal MP accumulation (Fig. 5 I-L) and their potential toxicity (Fig. 6). We permanently marked the circulating monocytes with repeated EdU injections prior to and during light-induced subretinal recruitment in Cx3cr1$^{-/-}$ mice and compared the numbers of subretinal EdU$^{+}$ MPs in mice with and without clodronate-liposome-induced circulating monocyte depletion. Local EdU administration failed to mark subretinal MPs, suggesting that ocular MP proliferation does not play a significant role in light-induced accumulation. Using this experimental setup, 37% of subretinal MPs were EdU positive in mice receiving systemic EdU injections and control liposomes. This seems to suggest that at least one third of subretinal MPs originated from circulation. Considering that the EdU injections only marked 76% of circulating monocytes, the extent of Mo participation in light-induced subretinal MP accumulation using this method is possibly underestimated. Indeed, monocyte depletion inhibited the subretinal IBA-1$^{+}$ MP accumulation by 50 to 60% (Fig. 5), suggesting that as many as half of the subretinal MP population might originate from blood-borne Mo and half from local resident Mfs or MCs. Similarly, genetic Ccl2 or Ccr2 deletion and CCR2 inhibitors diminished subretinal MP accumulation by 50-60% in age- and light-challenge-induced in Cx3cr1$^{-/-}$and Cx3cr1$^{GFP/GFP}$ (Fig. 2, 3 and 7). The new results are presented in (Fig. 5I-L).*

*To better define the subpopulation's (Mo-derived MPs versus MCs) influence on photoreceptor degeneration, we performed a series of in vitro co-culture experiments. The photoreceptor toxicity of wildtype and Cx3cr1$^{-/-}$ Mos and MCs was evaluated on retinal explants (Fig. 6). Our results show that Mos and particularly Cx3cr1$^{-/-}$ Mos display photoreceptor toxicity compared to MCs and might explain the marked neuroprotective effect of monocyte depletion in light-challenged Cx3cr1$^{-/-}$ mice.*

In Figure 6 it is unlikely that inhibition of CCR2+ macrophages is specific for CxCR1 macrophage damage. Was this agent tried in other forms of retinal damage? As it is the CCR2 dependently recruited monocytes in the CX3CR1KO that confer the neurotoxic property

*Using several double knockout mouse strains and monocyte depletion experiments, we showed that CCL2 dependent CCR2+ monocte recruitment is a critical component in the subretinal MP accumulation of Cx3cr1deficient mice. To evaluate whether the pharmacological inhibition of CCR2 is able to also inhibit CX3CR1-dependent subretinal MP accumulation and photoreceptor degeneration, we treated light-challenged Cx3cr1$^{-/-}$ mice systemically with a CCR2 inhibitor (RS 102895, Tocris). This inhibitor is described as blocking CCR2 mediated signalling*

*(http://www.tocris.com/dispprod.php?ItemId=104317#.Ue7AAxZTPUY). The inhibition of subretinal MP accumulation and photoreceptor degeneration by pharmacological means thereby reproduces the results obtained by genetic deletion and monocyte depletion.*

***References:***

Anastasopoulos E, Kakoulidou A, Coleman AL, Sinsheimer JS, Wilson MR, Yu F, Salonikiou A, Koskosas A, Pappas T, Founti P, Lambropoulos A, Topouzis F (2012) Association of sequence variation in the CX3CR1 gene with geographic atrophy age-related macular degeneration in a Greek population. *Curr Eye Res* **37**(12)**:** 1148-1155

Cardona AE, Pioro EP, Sasse ME, Kostenko V, Cardona SM, Dijkstra IM, Huang D, Kidd G, Dombrowski S, Dutta R, Lee JC, Cook DN, Jung S, Lira SA, Littman DR, Ransohoff RM (2006) Control of microglial neurotoxicity by the fractalkine receptor. *Nat Neurosci* **9**(7)**:** 917-924

Chen M, Forrester JV, Xu H (2011) Dysregulation in retinal para-inflammation and age-related retinal degeneration in CCL2 or CCR2 deficient mice. *PLoS One* **6**(8)**:** e22818

Combadiere C, Feumi C, Raoul W, Keller N, Rodero M, Pezard A, Lavalette S, Houssier M, Jonet L, Picard E, Debre P, Sirinyan M, Deterre P, Ferroukhi T, Cohen SY, Chauvaud D, Jeanny JC, Chemtob S, Behar-Cohen F, Sennlaub F (2007) CX3CR1-dependent subretinal microglia cell accumulation is associated with cardinal features of age-related macular degeneration. *J Clin Invest* **117**(10)**:** 2920-2928

Cruz-Guilloty F, Saeed AM, Echegaray JJ, Duffort S, Ballmick A, Tan Y, Betancourt M, Viteri E, Ramkhellawan GC, Ewald E, Feuer W, Huang D, Wen R, Hong L, Wang H, Laird JM, Sene A, Apte RS, Salomon RG, Hollyfield JG, Perez VL (2013) Infiltration of proinflammatory m1 macrophages into the outer retina precedes damage in a mouse model of age-related macular degeneration. *Int J Inflam* **2013:** 503725

Gautier EL, Shay T, Miller J, Greter M, Jakubzick C, Ivanov S, Helft J, Chow A, Elpek KG, Gordonov S, Mazloom AR, Ma'ayan A, Chua WJ, Hansen TH, Turley SJ, Merad M, Randolph GJ (2012) Gene-expression profiles and transcriptional regulatory pathways that underlie the identity and diversity of mouse tissue macrophages. *Nat Immunol* **13**(11)**:** 1118-1128

Ginhoux F, Greter M, Leboeuf M, Nandi S, See P, Gokhan S, Mehler MF, Conway SJ, Ng LG, Stanley ER, Samokhvalov IM, Merad M (2010) Fate mapping analysis reveals that adult microglia derive from primitive macrophages. *Science* **330**(6005)**:** 841-845

Guo C, Otani A, Oishi A, Kojima H, Makiyama Y, Nakagawa S, Yoshimura N (2012) Knockout of ccr2 alleviates photoreceptor cell death in a model of retinitis pigmentosa. *Exp Eye Res* **104:** 39-47

Gupta N, Brown KE, Milam AH (2003) Activated microglia in human retinitis pigmentosa, late-onset retinal degeneration, and age-related macular degeneration. *Exp Eye Res* **76**(4)**:** 463-471

Kohno H, Chen Y, Kevany BM, Pearlman E, Miyagi M, Maeda T, Palczewski K, Maeda A (2013) Photoreceptor Proteins Initiate Microglial Activation via Toll-like Receptor 4 in Retinal Degeneration Mediated by All-trans-retinal. *J Biol Chem* **288**(21)**:** 15326-15341

© EMBO

Levy OE, Calippe BE, Raoul W, Camelo S, Lavalette S, Guillonneau X, Combadiere C, Sennlaub F (2011) CX3CR1 Deficient Macrophages Present An Impaired Clearance From The Subretinal Space. *ARVO Meeting Abstracts* **52**(6)**:** 968

Luhmann UF, Robbie S, Munro PM, Barker SE, Duran Y, Luong V, Fitzke FW, Bainbridge J, Ali RR, Maclaren R (2009) The drusen-like phenotype in aging Ccl2 knockout mice is caused by an accelerated accumulation of swollen autofluorescent subretinal macrophages. *Invest Ophthalmol Vis Sci*

Ma W, Zhao L, Fontainhas AM, Fariss RN, Wong WT (2009) Microglia in the mouse retina alter the structure and function of retinal pigmented epithelial cells: a potential cellular interaction relevant to AMD. *PLoS One* **4**(11)**:** e7945

Nakazawa T, Hisatomi T, Nakazawa C, Noda K, Maruyama K, She H, Matsubara A, Miyahara S, Nakao S, Yin Y, Benowitz L, Hafezi-Moghadam A, Miller JW (2007) Monocyte chemoattractant protein 1 mediates retinal detachment-induced photoreceptor apoptosis. *Proc Natl Acad Sci U S A* **104**(7)**:** 2425-2430

Ransohoff RM (2009) Chemokines and chemokine receptors: standing at the crossroads of immunobiology and neurobiology. *Immunity* **31**(5)**:** 711-721

Ransohoff RM, Cardona AE (2010) The myeloid cells of the central nervous system parenchyma. *Nature* **468**(7321)**:** 253-262

Raoul W, Feumi C, Keller N, Lavalette S, Houssier M, Behar-Cohen F, Combadiere C, Sennlaub F (2008) Lipid-bloated subretinal microglial cells are at the origin of drusen appearance in CX3CR1-deficient mice. *Ophthalmic Res* **40**(3-4)**:** 115-119

Rutar M, Natoli R, Provis JM (2012) Small interfering RNA-mediated suppression of Ccl2 in Muller cells attenuates microglial recruitment and photoreceptor death following retinal degeneration. *J Neuroinflammation* **9:** 221

Rutar M, Natoli R, Valter K, Provis JM (2011) Early focal expression of the chemokine Ccl2 by Muller cells during exposure to damage-inducing bright continuous light. *Invest Ophthalmol Vis Sci* **52**(5)**:** 2379-2388

Silverman MD, Zamora DO, Pan Y, Texeira PV, Baek SH, Planck SR, Rosenbaum JT (2003) Constitutive and inflammatory mediator-regulated fractalkine expression in human ocular tissues and cultured cells. *Invest Ophthalmol Vis Sci* **44**(4)**:** 1608-1615

Simpson JE, Newcombe J, Cuzner ML, Woodroofe MN (1998) Expression of monocyte chemoattractant protein-1 and other beta-chemokines by resident glia and inflammatory cells in multiple sclerosis lesions. *J Neuroimmunol* **84**(2)**:** 238-249

Suzuki M, Tsujikawa M, Itabe H, Du ZJ, Xie P, Matsumura N, Fu X, Zhang R, Sonoda KH, Egashira K, Hazen SL, Kamei M (2012) Chronic photo-oxidative stress and subsequent MCP-1 activation as causative factors for age-related macular degeneration. *J Cell Sci* **125**(Pt 10)**:** 2407-2415

Tuo J, Smith BC, Bojanowski CM, Meleth AD, Gery I, Csaky KG, Chew EY, Chan CC (2004) The involvement of sequence variation and expression of CX3CR1 in the pathogenesis of age-related macular degeneration. *Faseb J* **18**(11)**:** 1297-1299

Wynn TA, Chawla A, Pollard JW (2013) Macrophage biology in development, homeostasis and disease. *Nature* **496**(7446)**:** 445-455

Yang X, Hu J, Zhang J, Guan H (2010) Polymorphisms in CFH, HTRA1 and CX3CR1 confer risk to exudative age-related macular degeneration in Han Chinese. *Br J Ophthalmol*

2nd Editorial Decision 14 August 2013

Thank you for the submission of your revised manuscript to EMBO Molecular Medicine. We have now received the enclosed reports from the two Reviewers that were asked to re-assess it. As you will see below, the Reviewers are now globally supportive with some final requests, and I am pleased to inform you that we will be able to accept your manuscript pending the following final amendments (which will be dealt with editorially):

1) Reviewer 2 feels that your manuscript would benefit from some extensive editing. I suggest that you make an effort in this sense. Our own copy-editing process will take care of the typographical errors and obvious grammatical errors but cannot significantly improve overall readability. Reviewer 3 had also expressed concerns in this respect. I must say that I agree that overall, the manuscript could be improved and to increase impact and delivery of the message.

2) Reviewer 3 would like you to recognise the weakness of the acute light damage model clearly. Also, s/he notes that reciprocal control of macrophage recruitment and activity via CX3CR1 and CCR2 in the model should be commented. I would like to add that this (important) point perhaps could emerge more clearly in the Abstract.

3) As per our Author Guidelines, the description of all reported data that includes statistical testing must state the name of the statistical test used to generate error bars and P values, the number (n) of independent experiments underlying each data point (not replicate measures of one sample), and the actual P value for each test (not merely 'significant' or 'P < 0.05').

Please also provide a list of changes with the indication of where they were applied when you submit your revised manuscript.

I look forward to reading a new revised version of your manuscript as soon as possible and in any case, within two weeks.

***** Reviewer's comments *****

Referee #2 (Remarks):

The authors are to be congratulated with making multiple improvements to the manuscript that strengthen their conclusions. In particular, they have provided data showing a) CCR2 is expressed in MPs in human AMD samples, b) the involvement of monocytes that have been labeled using an alternative technique to CCR2+ IHC, and c) demonstrating the direct toxicity of monocytes to photoreceptors. The manuscript is also improved in the quality of its writing, particularly in the Introduction and Discussion. However, another round of editing to remove typographical errors and some awkwardness of expression in parts of the manuscript can still help the manuscript read a little better.

© EMBO 

Referee #3 (Remarks):

The weakness of the acute light damage model should be recognised clearly.
The reciprocal control of macrophage recruitment and activity via CX3CR1 and CCR2 in this model is interesting and may reflect acute versus chronic inflammation. A comment to this effect might be important.

2nd Revision - authors' response 28 August 2013

*The manuscript has been revised according to your suggestions:*

1) Reviewer 2 feels that your manuscript would benefit from some extensive editing. I suggest that you make an effort in this sense. Our own copy-editing process will take care of the typographical errors and obvious grammatical errors but cannot significantly improve overall readability. Reviewer 3 had also expressed concerns in this respect. I must say that I agree that overall, the manuscript could be improved and to increase impact and delivery of the message.

*The revised manuscript has been edited in that sense. In particular parts of the result section of "CCL2 deficiency protects Cx3cr1$^{-/-}$ mice from age- and light-induced photoreceptor degeneration" and "Subretinal MP accumulation in Cx3cr1$^{-/-}$ mice is dependent on CCL2 mediated monocyte recruitment" have been rephrased to facilitate the understanding. A pdf file highlighting all the changes of the revised manuscript was submitted separately.*

2) Reviewer 3 would like you to recognise the weakness of the acute light damage model clearly. Also, s/he notes that reciprocal control of macrophage recruitment and activity via CX3CR1 and CCR2 in the model should be commented. I would like to add that this (important) point perhaps could emerge more clearly in the Abstract.

*To further recognize the differences of acute versus chronic subretinal mononuclear phagocyte accumulation we emphasized these differences in the discussion:*

*"Although **aged** Cx3cr1-deficient mice do not mimic all aspects of AMD (drusen formation and RPE atrophy) they do model **chronic** subretinal MP accumulation and associated photoreceptor degeneration, which are hallmarks of AMD" (page 23)*

*"Exaggerated subretinal MP accumulation and photoreceptor degeneration can also be induced **acutely** by a **light-challenge** in Cx3cr1 knockout mice (Raoul et al, 2008a)." (page 23)*

*"Interestingly, **although light-induced subretinal MP accumulation represents an acute inflammation and might therefore be quite different from the chronic inflammation observed with age**, the inhibition of CCL2/CCR2 prevented subretinal inflammation in the same order of magnitude." (page 26).*

*To further emphasize the differences in the roles of Cx3cr1 and Ccl2/Ccr2 (which is the subject of the whole experimental part of this manuscript) we specified in the title the use of the Cx3cr1 knockout mouse as the model of subretinal inflammation (experimental subretinal inflammation **in Cx3cr1 deficient mice**). As you suggested we also added this important aspect to the abstract:*

*"Our study shows that contrary to CCR2 and CCL2, CX3CR1 is constitutively expressed in the retina where it represses the expression of CCL2 and the recruitment of neurotoxic inflammatory CCR2$^{+}$ monocytes. "*

3) As per our Author Guidelines, the description of all reported data that includes statistical testing must state the name of the statistical test used to generate error bars and P values, the number (n) of independent experiments underlying each data point (not replicate measures of one sample), and the actual P value for each test (not merely 'significant' or 'P < 0.05').

© EMBO

*As mentioned in the Material and method section, statistical analysis was performed using Graph Pad Prism 5 by one-way or two-way Anova analysis of variance followed by Bonferroni post-test, students t-test, or Mann–Whitney test for comparison among means depending on the experimental design. The number of independent experiments (n), exact p values are indicated in the figure legends for students and Mann-Whitney tests and significance levels, as calculated by Prism 5, are indicated for Anova tests (http://www.graphpad.com/support/faqid/189/).*
