# Supplementary material for: CCR2+ monocytes infiltrate atrophic lesions in age-related macular disease and mediate photoreceptor degeneration in experimental subretinal inflammation in Cx3cr1 deficient mice
Source: EMBO Mol Med. 2013 Oct 21;5(11):1775–93. doi: 10.1002/emmm.201302692 (PMC3840491; doi:10.1002/emmm.201302692)
Supplement: Supplementary file 2 [file emmm0005-1775-SD2.pdf]

Supplementary data Sennlaub et al:

Table: Demographic data of the study group of patients with geographic atrophy and the control group of patients without retinal disorders and undergoing routine cataract surgery 2

CCR2 immunohistochemistry on healthy human blood smear 3

Negative control and examples of CCR2+immunohistochemistry on sections with geographic atrophy and age related maculopathy 4

Intravitreal EdU injections in light-challenged Cx3cr1$^{-/-}$mice 5

Comparaison of subretinal MP accumulation in C57BL/6, Ccl2$^{-/-}$ and Cx3cr1$^{-/-}$ mice at 18 months under the same animal housing conditions. 6

Photoreceptor status in 3-month-old C57BL/6 and Cx3cr1$^{-/-}$ mice 8

References: 9

Table: Demographic data of the study group of patients with geographic atrophy and the control group of patients without retinal disorders and undergoing routine cataract surgery

Characteristic	study group (n=18)	control group (n=22)	p (value)
Age, mean (SD)	80,1 (8,9)	76,2 (7,8)	0,14 (student)
Women, n (%)	13 (72,2)	13 (59,1)	0,38 (chi2)

CCR2 immunohistochemistry on healthy human blood smear

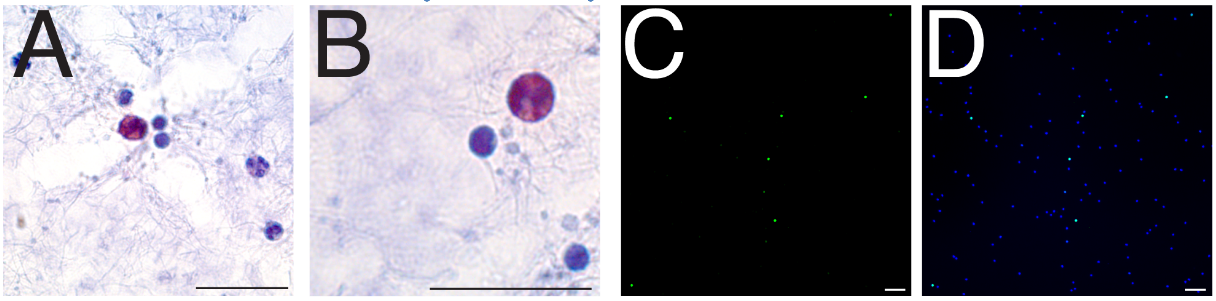

A-D: CCR2 immunohistochemistry using citrate buffer heat antigen retrieval as for the paraffin section experiments, on human blood smear preparations. A and B red staining, C and D green staining, D merged with Hoechst nuclear stain in blue. Scale bar c = 50μm.

Negative control and examples of $CCR2^{+}$immunohistochemistry on sections with geographic atrophy and age related maculopathy

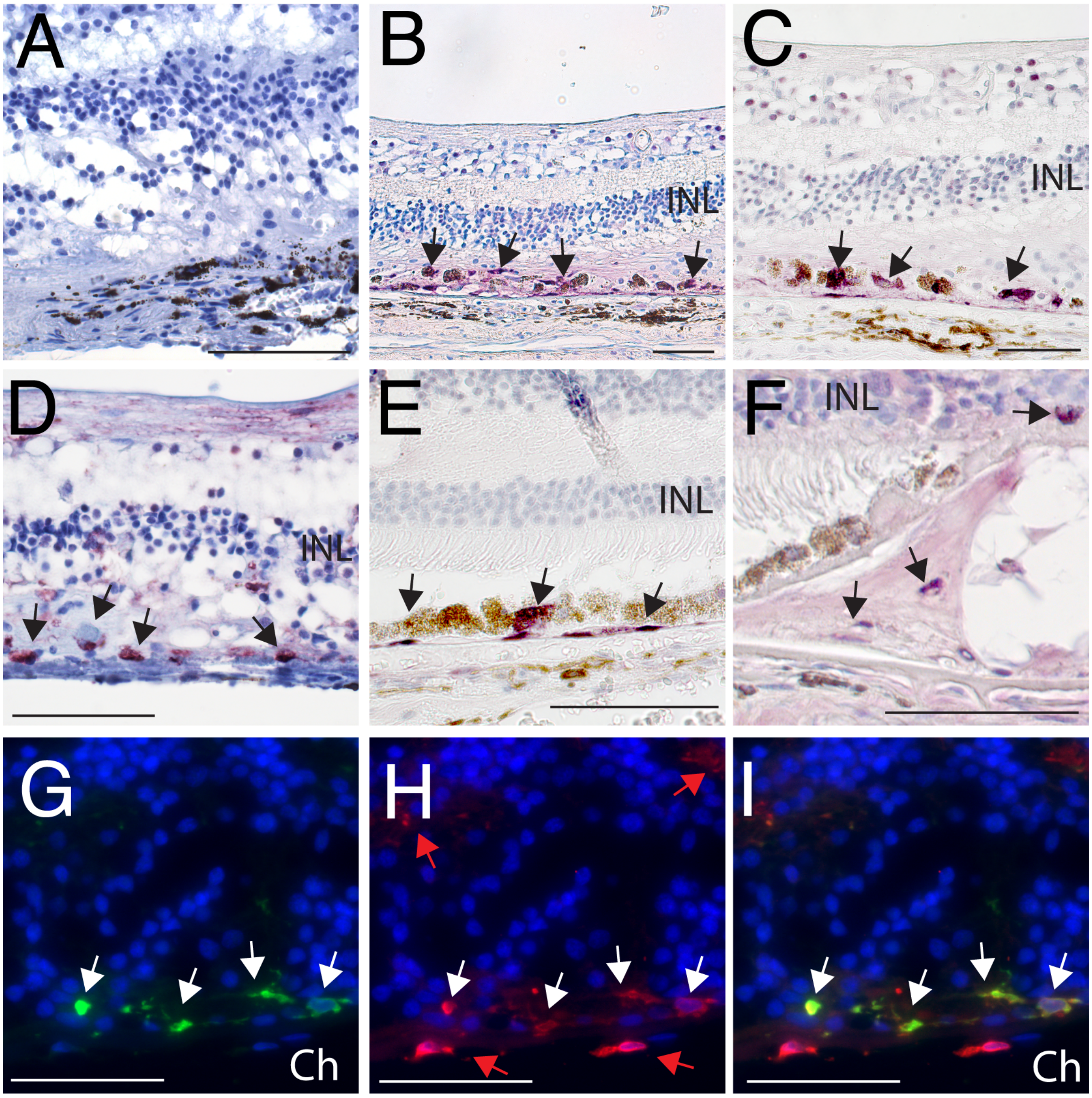

A) Control staining omitting the primary anti-CCL2/CCR2 antibody of a section containing the GA lesion. B-F) CCR2 staining (red) on macular GA lesion (B and C), extramacular GA lesion (D), laminar deposit (E) and soft drusen (F). G) CCR2 (green staining), H) CD18 (red staing), I) merge double labeling of a GA lesion. INL: inner nuclear layer. INL: inner nuclear layer; Ch: choroid. Scale bar a and b = 100μm; Scale bar c = 50μm.

Intravitreal EdU injections in light-challenged Cx3cr1$^{-/-}$mice

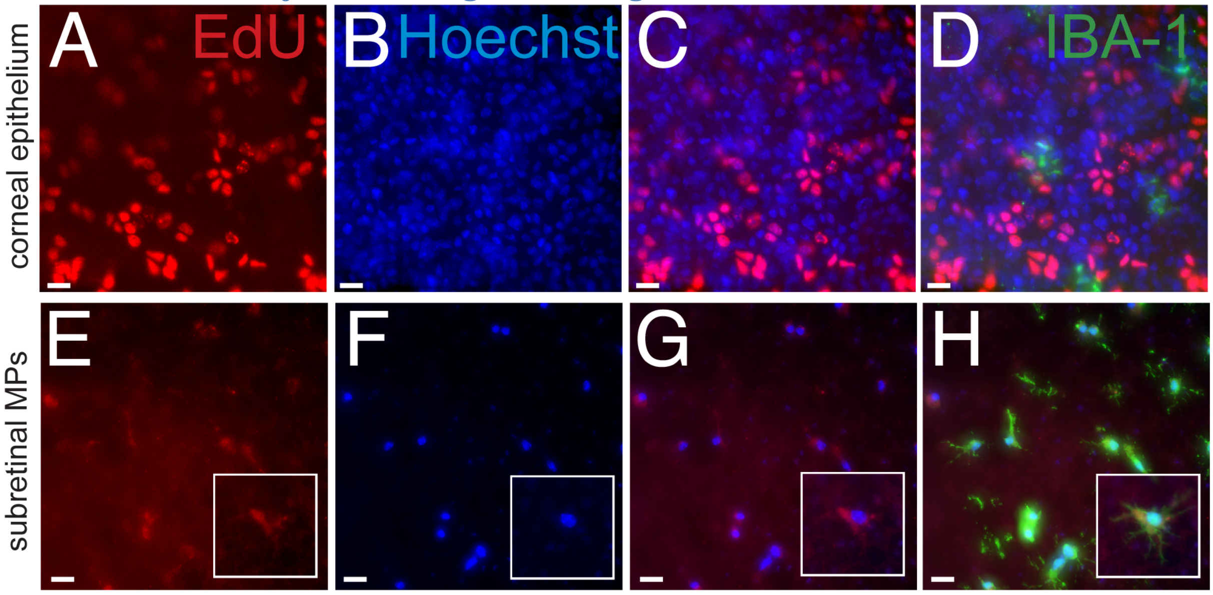

To evaluate the participation of local proliferation of MPs in the accumulation of subretinal MPs, 2µl of 30mg/ml EdU was injected intravitreally (n=4) at d2 (before significant subretinal MP accumulation occurs) and EdU (A and E, red), Hoechst (B and F, blue; merged with EdU C and G), IBA-1 labelling (D and H, green labelling, merged with EdU and Hoechst) were performed on corneal (A-D) and RPE/choroidal (E-F) flatmounts. Numerous EdU positive, Hoechst positive basal epithelial cells were observed in the corneal epithelium (A-C) indicating that physiologically proliferating cells were marked with this protocol. On the other hand subretina IBA^{+}MPs revealed some degree of extranuclear autofluorescence, but the nuclei had not incorporated the locally injected tracable nucleotide EdU, suggesting that subretinal MPs existed prior to EdU injection or proliferated outside the eye. Scale bar = 10µm.

Comparaison of subretinal MP accumulation in C57BL/6, Ccl2$^{-/-}$ and Cx3cr1$^{-/-}$ mice at 18 months under the same animal housing conditions.

Subretinal mononuclear phagocytes (MPs) accumulate with age in C57BL/6 mice (Xu et al, 2008) and light dependently in albino strains (Ng & Streilein, 2001). Deletion of Cx3cr1 leads to increased MP accumulation, quantified on IBA1 stained RPE/choroidal- and retinal-flatmounts, on a pigmented C57BL/6 (100% to 500% increase) (Chinnery et al, 2011; Combadiere et al, 2007) and albino BALB background (300% increase at 2 months) (Chinnery et al, 2011; Combadiere et al, 2007); in Cx3cr1$^{-/-}$ knockout (Combadiere et al, 2007) and Cx3cr1$^{GFP/GFP}$ knockin mice (Chinnery et al, 2011; Combadiere et al, 2007). In some animal housing conditions MP accumulation can reache a massif 180 MPs/mm^2 in both genotypes at 20-months (Chinnery et al, 2011) (which is far above any described subretinal MPs density under normal light conditions, even in albino animals (Ng & Streilein, 2001)) and in these conditions a CX3CR1 dependent difference is no longer observed.

On the other hand, Ccl2 and Ccr2 knockout animals have also been described to present a non-significant increase of subretinal MPs of about 30% in Ccr2$^{-/-}$, and a significant increase of 50% in Ccl2$^{-/-}$ mice compared to wiltype mice at 20 months only, quantified on IBA-1 stained flatmounts (Chen et al, 2011). This increase has been confirmed by quantifications on histological sections, aged 20 months and older (Luhmann et al, 2009).

It is difficult to compare subretinal MP accumulations between different laboratories as they vary greatly with the housing conditions (ambiant light, diet etc). To get a comparative idea of the influence of CCL2 and CX3CR1 deficiency on age-related MP accumulation, we compared subretinal MP in 3 and 18 month old C57BL/6, Ccl2$^{-/-}$ and Cx3cr1$^{-/-}$ mice kept under the same conditions. We observe a significant 2fold increase in subretinal IBA-1 positive MPs in Ccl2$^{-/-}$ mice (quantified on RPE/choroidal flatmounts) at 18 months compared to C57BL/6 mice raised under the same conditions (a). However, the

accumulation in 18 months-old Cx3cr1$^{-/-}$ mice was 7fold that of age-matched C57BL/6 mice and very significantly increased compared to both C57BL/6 and Ccl2$^{-/-}$ mice (b), as observed at 9 and 12 months (Fig. 3).

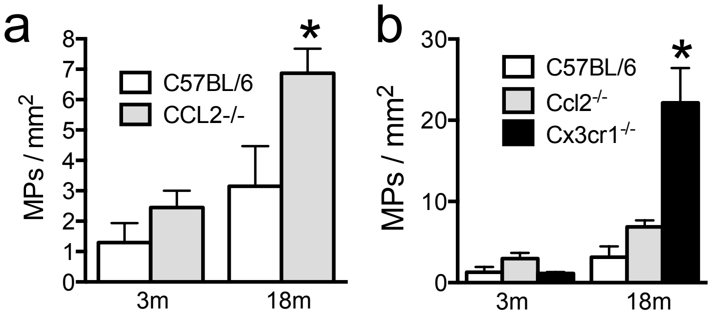

Photoreceptor status in 3-month-old C57BL/6 and Cx3cr1$^{-/-}$ mice

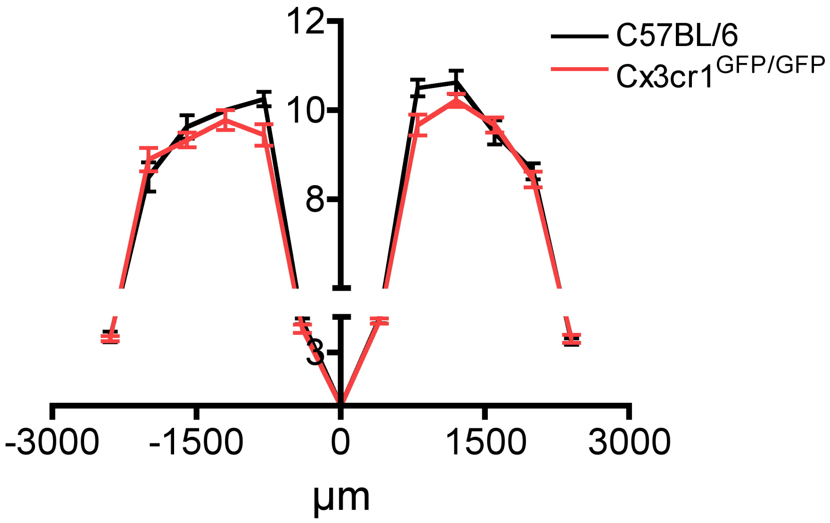

Photoreceptor cell nuclei were quantified in 3-month old C57BL/6 and Cx3cr1$^{-/-}$ mice by counting photoreceptor nuclei rows at increasing distances from the optic nerf and calculated as the area under the curve. There was no difference in photoreceptor cell numbers between the strains at this age.

References:

Chen M, Forrester JV, Xu H (2011) Dysregulation in retinal para-inflammation and age-related retinal degeneration in CCL2 or CCR2 deficient mice. *PLoS One* **6**(8)**:** e22818

Chinnery HR, McLenachan S, Humphries T, Kezic JM, Chen X, Ruitenberg MJ, McMenamin PG (2011) Accumulation of murine subretinal macrophages: effects of age, pigmentation and CX(3)CR1. *Neurobiol Aging*

Combadiere C, Feumi C, Raoul W, Keller N, Rodero M, Pezard A, Lavalette S, Houssier M, Jonet L, Picard E, Debre P, Sirinyan M, Deterre P, Ferroukhi T, Cohen SY, Chauvaud D, Jeanny JC, Chemtob S, Behar-Cohen F, Sennlaub F (2007) CX3CR1-dependent subretinal microglia cell accumulation is associated with cardinal features of age-related macular degeneration. *J Clin Invest* **117**(10)**:** 2920-2928

Luhmann UF, Robbie S, Munro PM, Barker SE, Duran Y, Luong V, Fitzke FW, Bainbridge J, Ali RR, Maclaren R (2009) The drusen-like phenotype in aging Ccl2 knockout mice is caused by an accelerated accumulation of swollen autofluorescent subretinal macrophages. *Invest Ophthalmol Vis Sci*

Ng TF, Streilein JW (2001) Light-induced migration of retinal microglia into the subretinal space. *Invest Ophthalmol Vis Sci* **42**(13)**:** 3301-3310

Xu H, Chen M, Manivannan A, Lois N, Forrester JV (2008) Age-dependent accumulation of lipofuscin in perivascular and subretinal microglia in experimental mice. *Aging Cell* **7**(1)**:** 58-68
